# Important Role of NH-Carbazole in Aryl Amination Reactions Catalyzed by 2-Aminobiphenyl Palladacycles

**DOI:** 10.1021/acscatal.3c00075

**Published:** 2023-03-07

**Authors:** Raquel J. Rama, Celia Maya, Francisco Molina, Ainara Nova, M. Carmen Nicasio

**Affiliations:** † Departamento de Química Inorgánica, Universidad de Sevilla, Aptdo 1203, 41071 Sevilla, Spain; ‡ Department of Chemistry, Hylleraas Centre for Quantum Molecular Sciences and Centre for Materials Science and Nanotechnology, University of Oslo, N-0315 Oslo, Norway; § Instituto de Investigaciones Químicas (IIQ), Departamento de Química Inorgánica and Centro de Innovación en Química Avanzada (ORFEO-CINQA), Consejo Superior de Investigaciones Científicas (CSIC) and Universidad de Sevilla, Avenida Américo Vespucio 49, 41092 Sevilla, Spain; ∥ Laboratorio de Catálisis Homogénea, Unidad Asociada al CSIC, CIQSO-Centro de Investigación en Química Sostenible and Departamento de Química, Universidad de Huelva, 21071 Huelva, Spain

**Keywords:** amination, palladacycle, phosphine, DFT calculations, microkinetic modeling, reaction mechanism

## Abstract

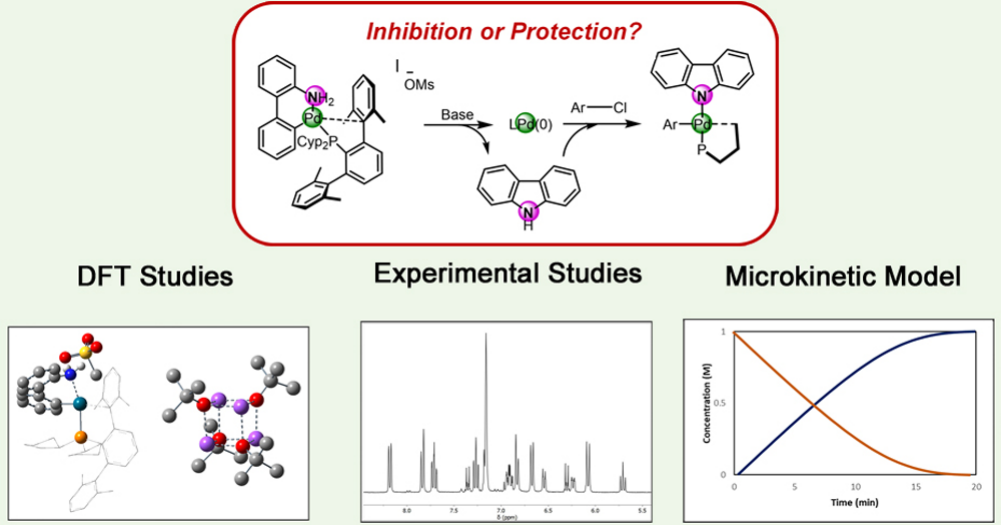

2-Aminobiphenyl palladacycles are among the most successful precatalysts for Pd-catalyzed cross-coupling reactions, including aryl amination. However, the role of NH-carbazole, a byproduct of precatalyst activation, remains poorly understood. Herein, the mechanism of the aryl amination reactions catalyzed by a cationic 2-aminobiphenyl palladacycle supported by a terphenyl phosphine ligand, PCyp_2_Ar^Xyl2^ (Cyp = cyclopentyl; Ar^Xyl2^ = 2,6-bis(2,6-dimethylphenyl)phenyl), **P1**, has been thoroughly investigated. Combining computational and experimental studies, we found that the Pd(II) oxidative addition intermediate reacts with NH-carbazole in the presence of the base (NaO^*t*^Bu) to yield a stable aryl carbazolyl Pd(II) complex. This species functions as the catalyst resting state, providing the amount of monoligated LPd(0) species required for catalysis and minimizing Pd decomposition. In the case of a reaction with aniline, an equilibrium between the carbazolyl complex and the on-cycle anilido analogue is established, which allows for a fast reaction at room temperature. In contrast, heating is required in a reaction with alkylamines, whose deprotonation involves coordination to the Pd center. A microkinetic model was built combining computational and experimental data to validate the mechanistic proposals. In conclusion, our study shows that despite the rate reduction observed in some reactions by the formation of the aryl carbazolyl Pd(II) complex, this species reduces catalyst decomposition and could be considered an alternative precatalyst in cross-coupling reactions.

## Introduction

**Scheme 1:**
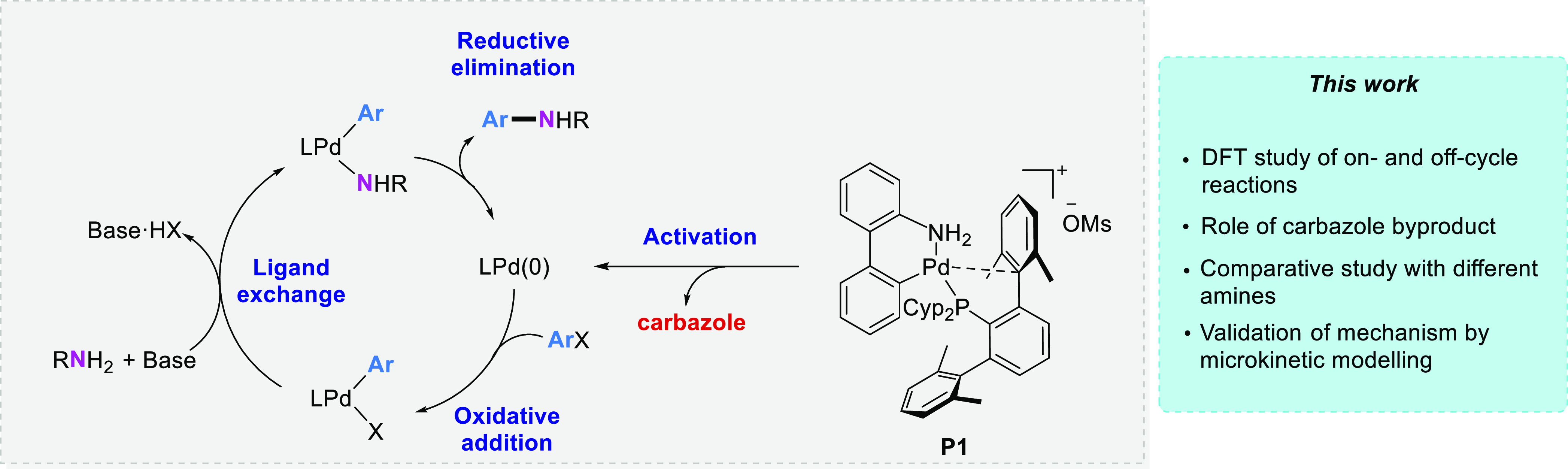
General Catalytic Cycle for the Buchwald–Hartwig Amination

The Pd-catalyzed aryl amination, known as the Buchwald–Hartwig reaction,^1,2^ is the most direct route for the synthesis of aromatic amines,^3−5^ useful intermediates for the chemical and pharmaceutical industry.^6−9^ Over the past 2 decades, a valuable collection of Pd-based catalyst systems has emerged that are very active for the coupling of (hetero)aryl chlorides with a wide range of challenging *N*-nucleophiles (*i.e.*, primary alkylamines, amides, *N*-heterocycles, and ammonia).^10−17^ Its common feature is that they are supported by sterically demanding and electron-rich ancillary phosphines and, to a lesser extent, *N*-heterocyclic carbene^18−20^ (NHCs) ligands. Prime examples of phosphine ligands used include bis-phosphines such as Josiphos^21,22^ and monophosphines like biaryl phosphines,^23−25^ CataCXium P,^26,27^ or Mor-DalPhos.^28^ In parallel with the ligand design, experimental^29−33^ and computational^33−39^ studies have been performed to understand the influence of the ligand, reactants, the base, and the solvent in the productive part of the catalytic cycle, namely, the oxidative addition, the ligand exchange, and the reductive elimination steps (Scheme 1). Furthermore, the key role of monoligated LPd(0)^40−42^ as the catalyst active species^34,35^ has also been rationalized.

On the contrary, off-cycle reactions such as catalyst activation are often ignored.^43^ The activation step usually involves the formal reduction of the stable Pd(II) precursor into the active monoligated LPd(0) species, affecting the overall rate and selectivity of the cross-coupling reaction.^44^ The analysis of the reduction to Pd(0) becomes more complex when the catalyst is produced *in situ* by mixing a Pd(II) salt with a large excess ligand.^45^ In recent years, the use of well-defined Pd(II) precatalysts with an optimal L/Pd ratio of 1:1 has led to considerable improvement in the effectiveness and applicability of cross-coupling reactions.^46,47^ This approach is more cost-effective than *in situ* protocols when sophisticated ligands are employed. However, the use of Pd(II) complexes as precatalysts introduces new players in the catalytic scenario, the spectator ligands. Such ligands are actively involved in the activation step, but they can also participate in additional off-cycle reactions, resulting in a decrease in catalytic activity. Evidence of this is the catalyst deactivation pathway found for [Pd(L)Cl(allyl)] (L = NHC, phosphine) precatalysts by formation of a Pd(I) dimer stabilized by a bridging allyl ligand.^48−51^

Palladacycles derived from 2-aminobiphenyl, supported by biaryl phosphines, are another family of Pd precatalysts widely used in cross-coupling reactions^52−54^ due to their easy activation under basic conditions.^53^ During their activation, NH-carbazole, arising from the 2-aminobiphenyl scaffold (*vide infra*), is released as a byproduct in the reaction media. An inhibiting effect of NH-carbazole has been documented in some cross-coupling reactions catalyzed by 2-aminobiphenyl-based palladacycles.^55−58^ Moreover, Colacot and co-workers have postulated, based on kinetic experiments, that the formation of a stable [Pd(L)(Ar)(carbazolyl)] complex resulting from the reaction of NH-carbazole and the Pd(II)-oxidative addition intermediate, which was characterized by X-ray diffraction, is responsible for the reduction in the catalytic activity.^57^ However, no computational studies of the complete catalytic cycle, including precatalyst activation, have been undertaken.

Recently, we examined the behavior of a family of dialkylterphenyl phosphines^59−61^ in Pd-catalyzed aryl amination reactions using 2-aminobiphenyl-derived palladacycles as precatalysts.^62,63^ We found that the cationic palladacycle with the sterically demanding phosphine PCyp_2_Ar^Xyl2^, **P1** (Scheme 1), displayed excellent performance and provided a broader substrate scope in the amination of deactivated aryl chlorides with *N*-nucleophiles, including primary and secondary alkyl and arylamines and *N*-heterocycles such as indoles (Table 1). To understand the reasons for the high activity and broad applicability of palladacycle **P1**, we decided to investigate the mechanism of aryl amination reactions catalyzed by **P1**, including the precatalyst activation step, by experiments and calculations. In this paper, we discuss the role of [Pd(L)(Ph)(carbazolyl)] species as the catalyst resting state, modulating the concentration of the Pd(0) active species, which enters the catalytic cycle. Furthermore, the comparative study using two different *N*-nucleophiles, anilines, and primary alkylamines helped identify two distinct pathways for the ligand exchange step. Finally, the proposed mechanism is validated and further analyzed using microkinetic modeling.

**Table 1:**
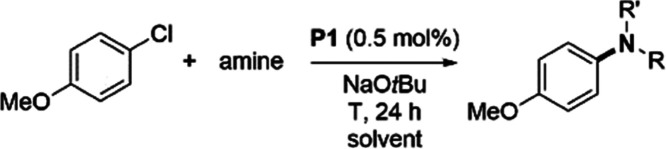

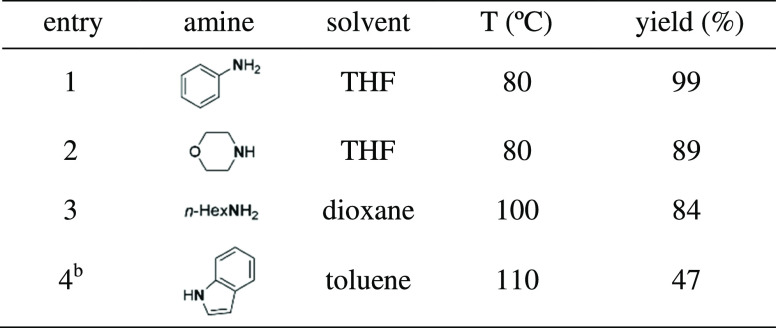
*N*-Arylation of Various *N*-Nucleophiles with the Precatalyst P1^a^

a Reaction conditions: aryl chloride (1 mmol), amine (1.2 mmol), [Pd] (0.5 mol %), NaO^*t*^Bu (1.2 mmol), THF (1 mL), 19 h (unoptimized). Yields of isolated products.

b Aryl chloride (0.5 mmol), indole (0.53 mmol), [Pd] (1 mol %), 18 h (unoptimized).

## Results and Discussion

The computational study was performed on the reaction of chlorobenzene with two amines, aniline and methylamine, as simplified models for the substrates presented in Table 1. For this study, we used density functional (DFT) methods (M06/def2SVP/SMD//M06/def2TZVP/SMD). The influence of the electronic properties of the substrates was also evaluated in both the oxidative addition and the reductive elimination steps. The base NaO*^t^*Bu was modeled in its tetrameric form considering the nonpolar solvent environment. Results using this model were found to be more consistent with experimental data than using the ^*t*^BuO^–^ anion (see the Supporting Information for details).

### Precatalyst Activation Step

**Scheme 2:**
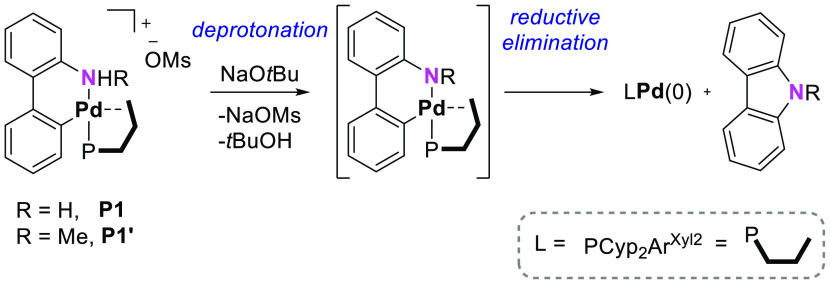
Activation of 2-Aminobiphenyl Palladacycles

It has been proposed^55,64,65^ that the reduction of 2-aminobiphenyl palladacycles to the monoligated LPd(0) species takes place by the deprotonation of the amino group in the presence of the base, followed by reductive elimination of NH-carbazole (Scheme 2).

**Figure 1:**
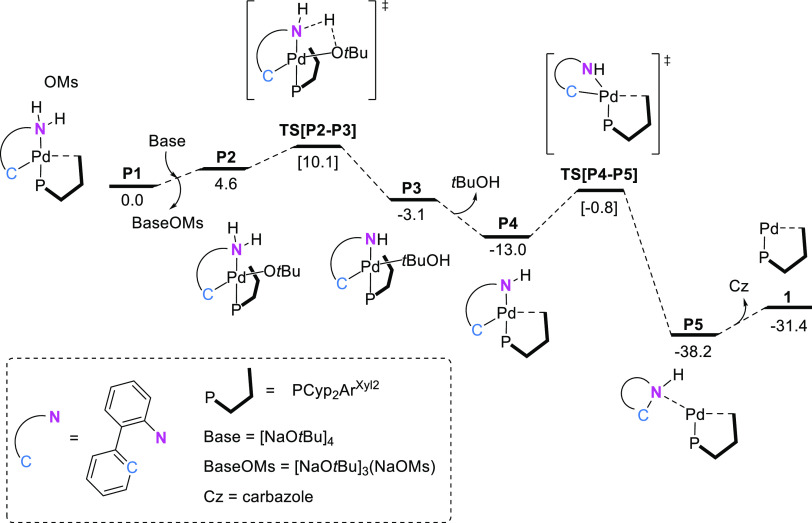
Gibbs energy profile for the precatalyst activation step. Energies in kcal mol^–1^.

An intramolecular pathway starting by the coordination of the base to the metal center followed by the deprotonation of the coordinated amino group in palladacycle **P1** is shown in Figure 1. In this pathway, the alkoxide group plays a dual role as a ligand and a base. This process involved the binding of ^*t*^BuO^–^ to the Pd(II) center through rotation of the terphenyl ring of the phosphine to form intermediate **P2**. The deprotonation and subsequent dissociation of *tert*-butanol produced intermediate **P4**, which underwent reductive elimination to give the (PCyp_2_Ar^Xyl2^)Pd(0) active species, **1**, and NH-carbazole as a byproduct. The overall process was highly exergonic, and both the deprotonation and the reductive elimination steps had activation barriers of only 10.1 and 11.3 kcal mol^–1^, respectively. An intermolecular pathway mediated by an external base was also considered unsuccessfully.^66^

**Scheme 3:**
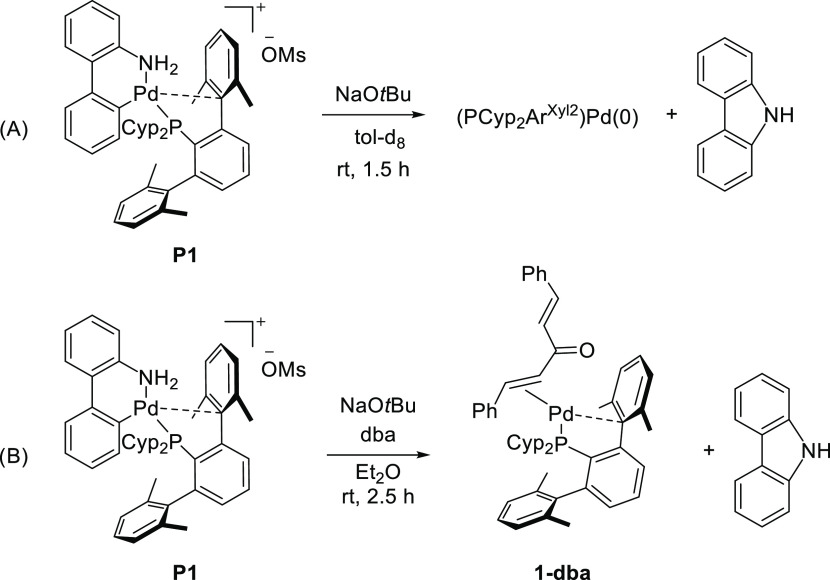
Reaction of **P1** with NaO^*t*^Bu under Various Conditions

Calculated energy barriers suggest that the activation of **P1** is fast under the catalytic conditions (*T* > 80 °C) and that they should be overcome at ambient temperature.^67^ To demonstrate the feasibility of **P1** activation at milder conditions, we carried out the reaction of **P1** with an excess of the base NaO^*t*^Bu (2 equiv) in toluene-*d*_8_ at room temperature (Scheme 3A). After 1.5 h, we observed the development of a black precipitate as a result of the decomposition of the purported monoligated LPd(0) species into metallic palladium. ^1^H NMR analysis of the reaction crude confirmed the formation of carbazole and ^*t*^BuOH in *ca*. 1:1 ratio (see the Supporting Information, Figure S14).

To trap the monoligated (PCyp_2_Ar^Xyl2^)Pd(0) species, we performed the reaction of the palladacycle with the base in the presence of dibenzylideneacetone, dba (5-fold excess), at room temperature (Scheme 3B). The olefin adduct, **1-dba**, was obtained as dark-orange crystals by storing a saturated diethyl ether solution at low temperature. However, its purification proved difficult as it was not possible to remove dba from the product even after several recrystallizations. To circumvent this problem, the complex **1-dba** was directly prepared from the reaction of Pd(CH_2_SiMe_3_)_2_(cod) with equimolar amounts of dba and phosphine in diethyl ether at room temperature (see the Supporting Information for details).

**Figure 2:**
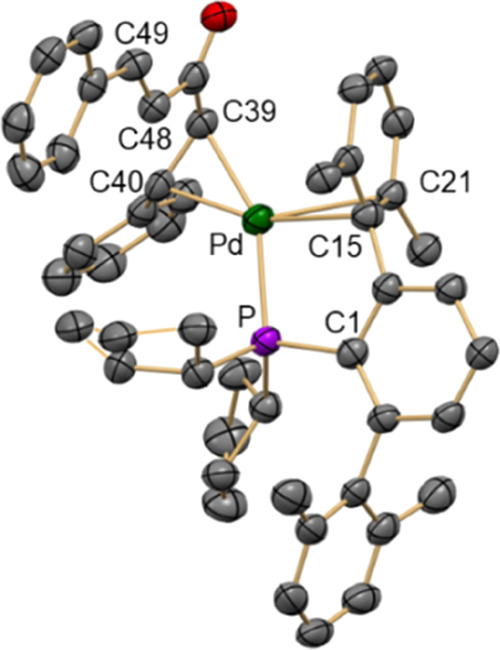
Molecular structure of **1-dba**. Hydrogen atoms are omitted for clarity and thermal ellipsoids are set at the 50% level probability. Selected distances [Å] and angles [°]: Pd–P 2.313(2), Pd–C39 2.162(8), Pd–C40 2.114(7), Pd–C15 2.329(8), Pd–C21 2.533(8), C39–C40 1.423(11), C48–C49 1.325(12).

The zero-valent complex **1-dba** is remarkably stable in solution and can be kept intact for longer periods under a nitrogen atmosphere. ^1^H and ^31^P NMR spectra of **1-dba** exhibited very broad signals at room temperature, which, upon cooling at −40 °C, resolved into two singlets at 55.6 and 54.0 ppm in the ^31^P NMR, in an approximate ratio of 1:8 (Figure S15). Since dba can adopt diverse conformations, different isomers are frequently observed for dba adducts in solution.^29−31,68−70^ The molecular structure of **1-dba** was elucidated by single-crystal X-ray diffraction (Figure 2). The Pd center is bonded to one of the alkene groups of the dba and exhibits a nonsymmetric η^2^–C_*ipso*,_–C*_ortho_* interaction with a closer flanking aryl ring of the phosphine. The shorter Pd–C*_ipso_* (C15) distance of 2.329(8) Å compares well to those found in analogous Pd(0) dba adducts supported by biaryl phosphine ligands (2.298–2.374 Å).^71−73^ However, the Pd lies at a longer distance from the C*_ortho_* (C21) atom (2.533(8) Å). The bond distances Pd–P (2.313(2) Å) and Pd–C_olefin_ (2.114(7) and 2.162(8) Å) are in the range found in the literature for similar complexes.^16,71−78^

### Oxidative Addition

**Figure 3:**
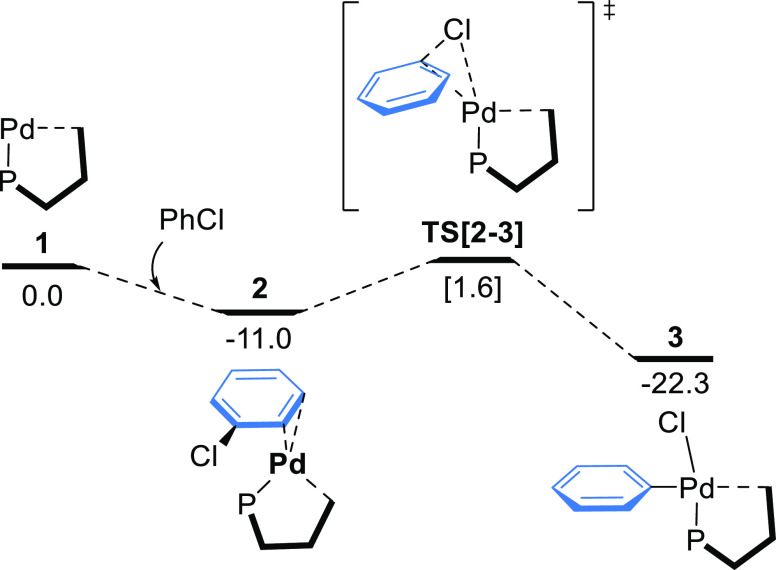
Gibbs energy profile for the oxidative addition step. Energies are in kcal mol^–1^.

It has been established that prior to oxidative addition, chlorobenzene interacts with monoligated Pd(0) species **1** through the aromatic ring forming an arene complex **2**(34,35,79) (Figure 3). We found that the lowest energy isomer (−11.0 kcal mol^–1^) shows η^2^-coordination with C*_ortho_* and C*_meta_* atoms of the PhCl ring (Figure S7). Intermediate **2** underwent oxidative addition rendering complex **3**. This step was a downhill process with a calculated barrier of 12.6 kcal mol^–1^ relative to **2**, in line with those reported for the reaction of chlorobenzene with a monoligated Pd(0) complex.^37,78,80^ The oxidative addition product **3** showed a *trans* orientation of the chloride and phosphine ligands. The low activation barrier found for the formation of **3** suggests that the oxidative addition of PhCl to **1** can also proceed at ambient temperature.

We found it interesting to evaluate the electronic effect of ArCl bearing electron-donating and -withdrawing groups at the *p*-position of the aryl ring in the oxidative addition step. For all of them, a conformational study of the possible isomers for intermediate **2** was also performed (Figures S7 and S8). As expected, the highest activation barrier for the **2** → **3** step was obtained for electron-rich 4-chloroanisole (16.0 kcal mol^–1^) and the lowest for electron-deficient 4-chlorobenzaldehyde (11.1 kcal mol^–1^).^81^ These energy barriers suggest that the oxidative addition should be feasible at room temperature, even for the less reactive substrates.

**Scheme 4:**
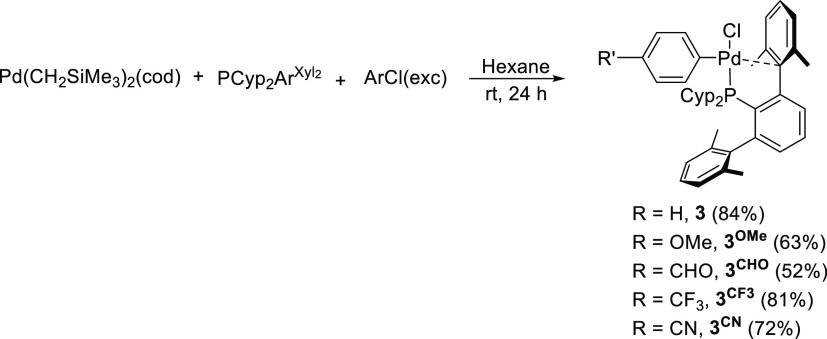
Synthesis of Oxidative Addition Products **3**

We isolated and structurally characterized a variety of oxidative addition complexes **3** following the procedure depicted in Scheme 4.^82,83^ These complexes were obtained as air-stable solids in moderate to good yields.^84^

**Figure 4:**
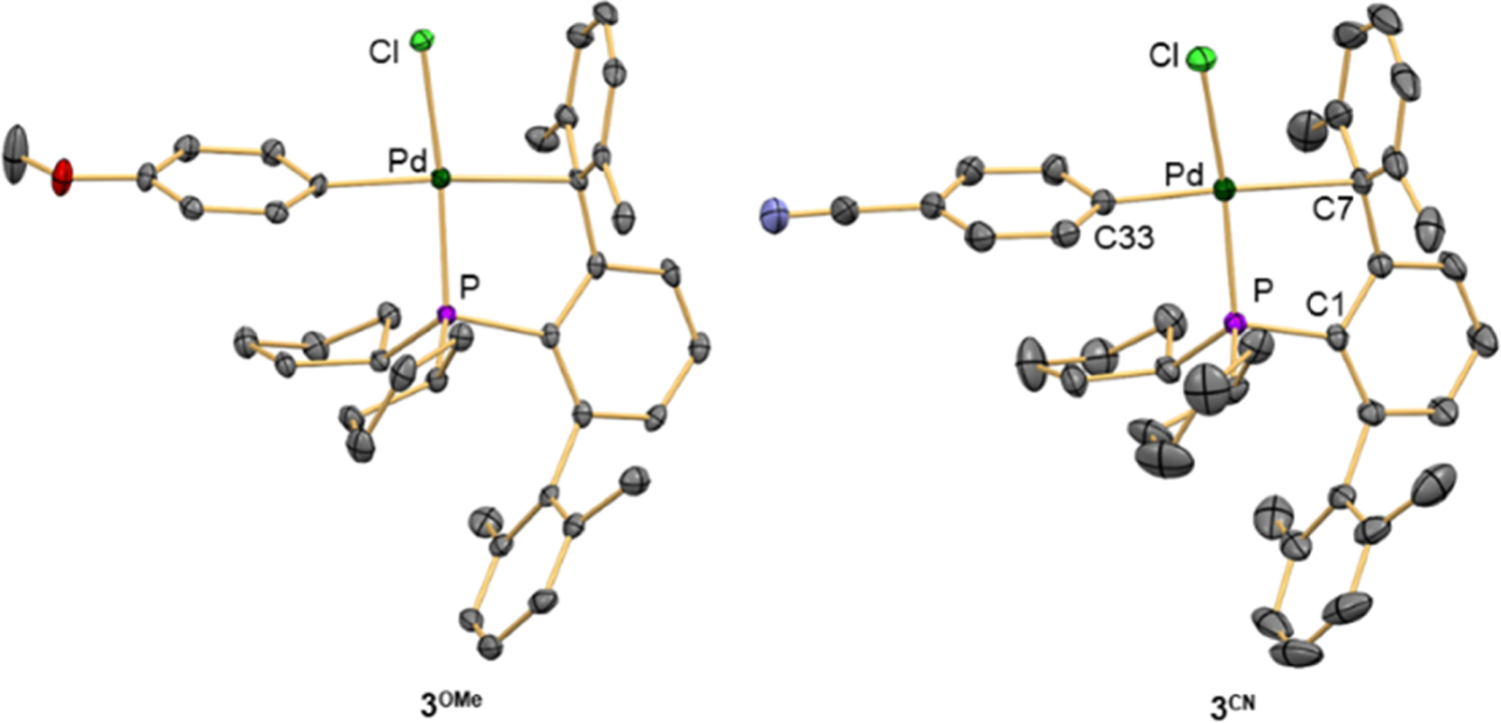
Molecular structures of oxidative addition complexes **3**^**OMe**^ and **3**^**CN**^. Hydrogen atoms are omitted for clarity, and thermal ellipsoids are set at the 50% level probability. Selected distances [Å] and angles [°] for **3**^**CN**^: Pd–P 2.2617(7), Pd–Cl 2.3471(7), Pd–C7 2.457(2), Pd–C33 1.992(3), P–C1 1.851(2); Cl–P–dC33 83.68(7), P–Pd–C7 83.06(7), P–Pd–Cl 169.32(3), C7–Pd–C33 162.17(10).

The ^31^P{^1^H} spectra of complexes **3** consisted of a single resonance at *ca*. 46 ppm (Δδ of *ca*. 40 ppm at a higher frequency with respect to the free ligand). This difference in ^31^P chemical shift,^60^ together with the observation of slow rotation of the phosphine ligand around the P–C*_ipso_* bond in their ^1^H NMR spectra, pointed toward a bidentate coordination mode of the terphenyl phosphine (k^2^-P, η^1^-C*_ipso_*). Structures of complexes **3** were established by X-ray diffraction studies carried out with **3**^**OMe**^(84) and **3**^**CN**^ (Figure 4).

Complexes **3** are mononuclear in the solid state. Both P and Cl atoms display a *trans*-arrangement, and the Pd(II) center features an η^1^ interaction with the *ipso*–carbon bond of the nearby side aryl ring of the phosphine. The Pd–C*_ipso_* contacts are rather long (2.426(2) Å for **3**^**OMe**^ and 2.457(2) Å for **3**^**CN**^) but fit within the range 2.22–2.45 Å found for the η^1^ coordinate arene to a d^8^-ML_3_ fragment^[Bibr ref]^ and compare well to those reported for biaryl phosphine analogues.^83^ The length of the Pd–aryl bond in both complexes is nearly identical (2.000(2) and 1.992(3) Å for **3**^**OMe**^ and **3**^**CN**^, respectively) despite the significant difference in the electron-donating ability of the aryl ring.

**Scheme 5:**
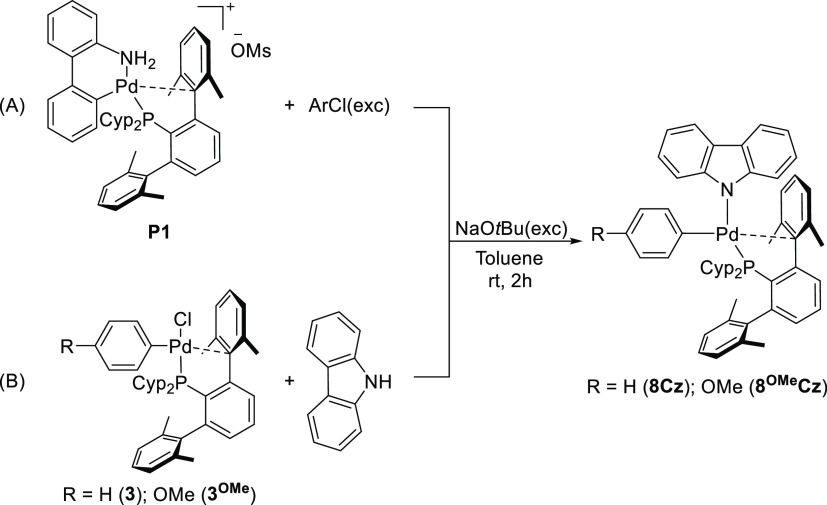
Synthesis of Carbazole-Containing Products **8Cz** and **8^OMe^Cz**

According to the calculations, the formation of intermediate **3** should take place rapidly at room temperature. To corroborate the computational finding, we studied the reaction of palladacycle **P1** with chlorobenzene in the presence of an excess of the base, NaO^*t*^Bu, at room temperature (Figure S18). To our surprise, the reaction did not produce the expected oxidative addition product but a Pd(II) complex containing a carbazolyl ligand, **8**^**OMe**^**Cz** (Scheme 5a). As suggested by Colacot, this compound may result from the reaction of the oxidative addition product **3** with a carbazolyl anion, coming from the deprotonation of the NH-carbazole byproduct (released during **P1** activation) in the presence of the base. To validate this hypothesis, we performed the synthesis of **8Cz** and **8**^**OMe**^**Cz** by reacting **3** and **3**^**OMe**^ with carbazole under basic conditions (Scheme 5b).

**Figure 5:**
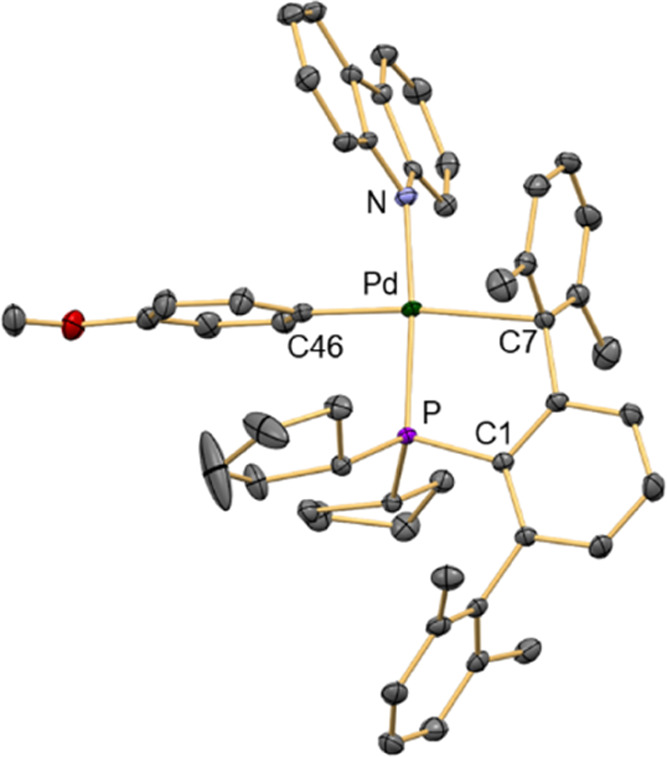
Molecular structure of **8**^**OMe**^**Cz**. Hydrogen atoms are omitted for clarity and thermal ellipsoids are set at the 50% level probability. Selected distances [Å] and angles [°]: Pd–P 2.2755(7), Pd–N 2.054(2), Pd–C7 2.456(3), Pd–C46 2.004(3), P–C1 1.853(3); N–Pd–C46 83.62(10), P–Pd–C7 82.92(6), C7–Pd–C33 162.17(10).

The complexes **8Cz** and **8**^**OMe**^**Cz** were isolated in high yields as air-stable orange crystalline solids. They were fully characterized by elemental analysis and NMR spectroscopy, and the structure of **8**^**OMe**^**Cz** was confirmed by X-ray crystallography. As shown in Figure 5, the carbazolyl ligand occupies the *trans*-position to the phosphine in the square planar coordination geometry. The Pd–C_*ipso*_ distance (2.456(3) Å) is similar to that found in oxidative addition products **3**^**OMe**^ and **3**^**CN**^. Furthermore, the Pd–N distance of 2.054(2) Å is similar to those of other tricoordinate monoligated Pd–amido complexes.^86^

X-ray structures of carbazolyl-containing complexes of late transition metals are scarce. We are aware of only one example of a Pd(II)–carbazolyl complex supported by the biaryl phosphine RuPhos described by Colacot and co-workers,^57^ whose structure is closely related to that of **8**^**OMe**^**Cz**. The role of the carbazolyl complex **8Cz** in the catalytic cycle will be discussed in detail below.

**Scheme 6:**
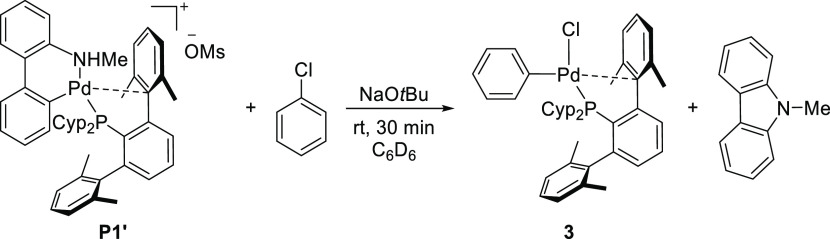
Formation of the Oxidative Addition Product 3 from **P1′**

To avoid the formation of the carbazolyl complex **8Cz**, palladacycle **P1′**, bearing the *N*-methyl-2-aminobiphenyl ligand, was employed in the reaction with chlorobenzene and the base (Scheme 6). The activation of **P1′** would render the formation of monoligated species **1** and *N*-methyl-carbazole as a byproduct, which could not be further deprotonated. NMR monitoring the reaction in C_6_D_6_ confirmed the formation of the expected oxidative addition product **3** along with the *N*-methylcarbazole byproduct (Figure S16).

### Ligand Exchange

**Figure 6:**
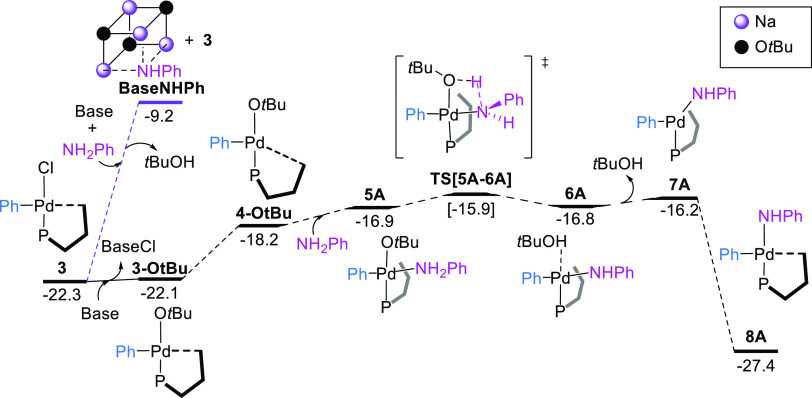
Gibbs energy profile for the reaction of **3** with aniline and the base. Gibbs energies are in kcal mol^–1^.

After the oxidative addition, the amine and the base (^*t*^BuO^–^) may compete for coordinating the metal center in **3**. We analyzed three different pathways for chloride replacement and amine deprotonation (see the Supporting Information for details). For aniline, we found that the most favorable route involved the substitution of the chloride ligand in **3** by ^*t*^BuO^–^ leading to the intermediate **3-OtBu** (Figure 6). Dissociation of the Pd–C_*ipso*_ interaction and aniline coordination produced the intermediate **5A** (A for aniline), which, after intramolecular deprotonation, dissociation of ^*t*^BuOH, and restoration of the Pd–C_*ipso*_ interaction, resulted in the formation of the amido complex **8A**, located at −27.4 kcal mol^–1^. The overall barrier for this pathway was 6.4 kcal mol^–1^, consistent with a rapid process. It should be noted that the alkoxide anion was acting both as a nucleophile^87^ and as a base, in contrast to the common role as a deprotonation agent found for ^*t*^BuO^–^ in reported computational studies.^34−39^ To confirm the computational prediction of a facile substitution of the chloride by the base in **3**, the reaction of complex **3** with NaO^*t*^Bu (10 equiv) at room temperature was monitored by ^31^P NMR spectroscopy. After 30 min of reaction, we observed a mixture of two species in *ca.* 1:3.7 ratio (Figure S17). The minor component corresponded to unreacted **3** and the major one to a new complex, which originated a signal at 37.6 ppm that was tentatively assigned to the alkoxide adduct. However, repeated attempts at isolating such species proved fruitless.

As aniline is a weak base (p*K*_a_ = 30.6 in DMSO) and a weak nucleophile, we also considered the possibility of the nonmetal-assisted deprotonation of aniline by the alkoxide base.^37^ Despite the higher energy of formation of the anilide anion (13.1 kcal mol^–1^, Figure 6), the nonmetal-assisted pathway should be accessible at room temperature.

**Figure 7:**
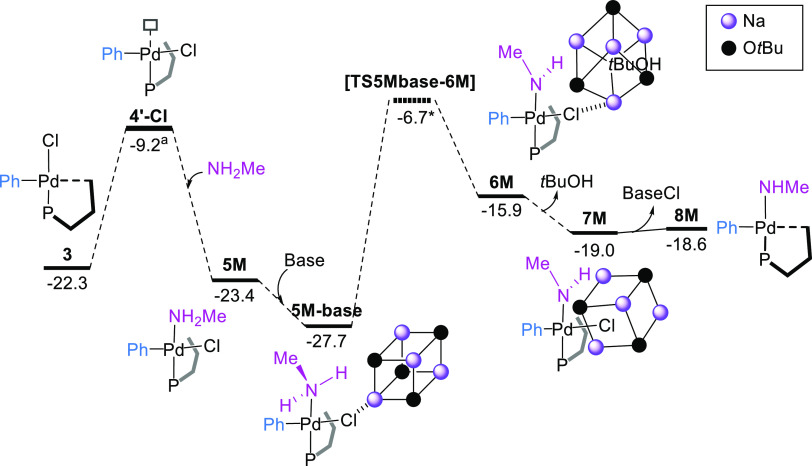
Gibbs energy profile for the reaction of complex **3** with methylamine and the base. Gibbs energies are in kcal mol^–1^. ^a^Structure optimized with a fixed Ph–Pd–Cl angle to estimate the TS energy between **3** and **5M**. *This value has been fitted using the microkinetic model.

A different scenario was found for a primary alkylamine. Using methylamine as the model substrate, the more feasible route agreed with those calculated previously^34−39^ and involves the direct coordination of the amine to an empty site *trans* to the P atom to give the intermediate **5M** (M for methylamine),^78,88,89^ which is 1.1 kcal mol^–1^ lower in energy than **3** (Figure 7 and Scheme S1). From **5M**, intermolecular deprotonation and chloride extraction take place, leading to the intermediate **8M** at −18.6 kcal mol^–1^. The transition state for the proton transfer could not be located, but this value was fitted to reproduce the experimental results using the microkinetic model (*vide infra*). Unlike aniline, the coordination/deprotonation of methylamine is an endergonic process.

**Scheme 7:**
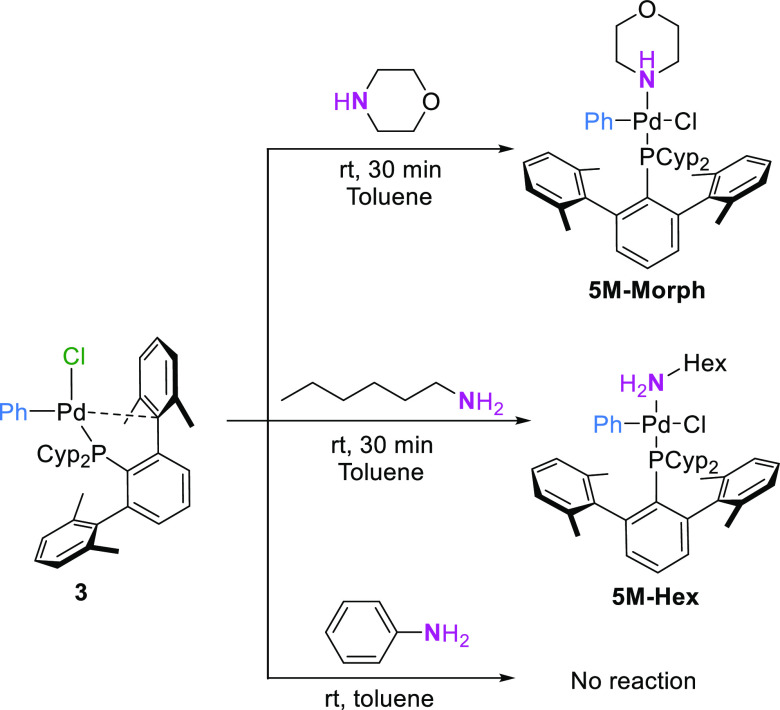
Synthesis of Amine Adducts

To support these results, we tested the reactivity of complex **3** with a large excess of hexylamine or morpholine in toluene, at room temperature (Scheme 7). Complexes **5M** were fully characterized by analytical and spectroscopic methods. In solution, both complexes dissociated the amine ligand, leading to mixtures of amino adducts and the oxidative addition product **3**. Efforts to grow crystals of any of the amino adducts suitable for X-ray diffraction studies proved unsuccessful. Conversely, no reaction was observed when **3** was treated with an excess of aniline, confirming the differences in the ligand exchange step for the two types of amines.

### Reductive Elimination

**Figure 8:**
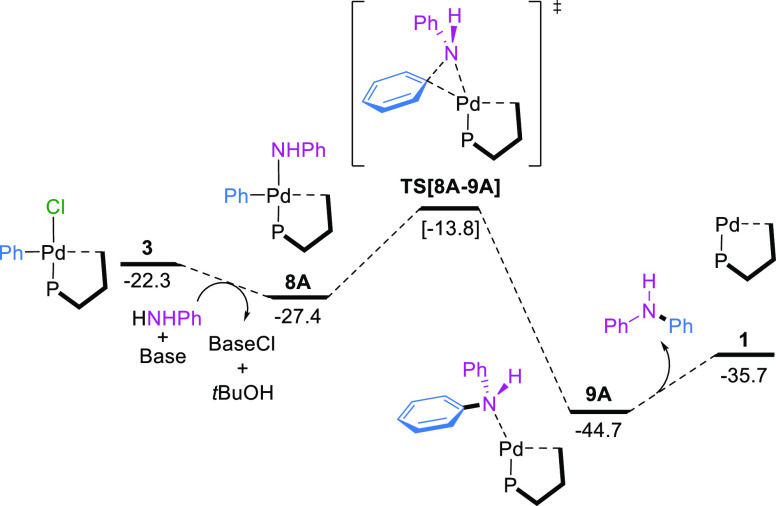
Gibbs energy profile for the reductive elimination step. Gibbs energies are in kcal mol^–1^.

In the last step of the catalytic cycle, the reductive elimination from the anilido complex **8A** proceeds with a barrier of only 13.6 kcal mol^–1^ to form the amine Pd(0) complex **9A** (−44.7 kcal mol^–1^). Substitution of diphenylamine by the chlorobenzene initiates a new catalytic cycle (Figure 8).

The influence of the electronic properties of both the aryl and amido groups on the reductive elimination step was taken into consideration. We calculated the reductive elimination barriers of anilido complexes bearing *p*-substituted aryl groups with different electronic properties (Table 2 and Figure S12). In agreement with previous experimental data,^90,91^ electron-rich aryl groups hampered the C–N reductive elimination, whereas electron-deficient aryl rings facilitated the process. The difference in the activation barrier between the least (4-MeO–C_6_H_4_) and most reactive (4-OHC–C_6_H_4_) anilido complexes was found to be 4.8 kcal mol^–1^. Moreover, barriers found for the C–N reductive elimination of amido ligands derived from primary alkylamine (methylamine), secondary amines (dimethyl amine and *N*-methylaniline), and *N*-heterocycle (carbazole) showed that the more electron-rich the amido group, the faster the reaction^90,91^ (Table 2 and Figure S13).

**Table 2:** Energies of Aryl Amido Complexes [(PCyp_2_Ar^Xyl2^)Pd(Ar)(amido)] and Reductive Elimination Transition States in kcal mol^–1^

aryl group	aryl anilido complex	transition state	Δ*G*^‡^
4-OHC-C_6_H_4_	–29.8	–20.3	9.5
4-CF_3_-C_6_H_4_	–28.3	–16.9	11.4
C_6_H_5_	–27.4	–13.8	13.6
4-OMe-C_6_H_4_	–26.7	–12.4	14.3

amido group	phenyl amido complex	transition state	Δ*G*^‡^
MeNH_2_	–18.6	–10.5	8.1
Me_2_NH	–20.5	–10.8	9.7
PhNH	–27.4	–13.8	13.6
PhMeN	–29.2	–13.5	15.7
carbazolyl	–39.3	–16.9	22.4

Interestingly, not only is the carbazolyl complex the most stable amido intermediate, but it is also 17.0 kcal mol^–1^ lower in energy than the oxidative addition product **3**. Such pronounced stability along with the substantial barrier of 22.4 kcal mol^–1^ for the reductive elimination facilitated the isolation of the carbazolyl intermediate **8Cz** from reactions outlined in Scheme 5. Efforts to prepare other alkyl or aryl amido complexes resulted in the formation of the C–N coupling product, supporting a facile reductive elimination step in these cases.

### Role of the Pd–Carbazolyl Complex

As described above, Colacot and co-workers detected the formation of a Pd–carbazolyl complex in aryl amination reactions catalyzed by a RuPhos-supported palladacycle and studied the reductive elimination of *N*-phenylcarbazole from Pd(C_6_H_5_)(carbazolyl)(RuPhos), independently prepared.^55−58^

**Figure 9:**
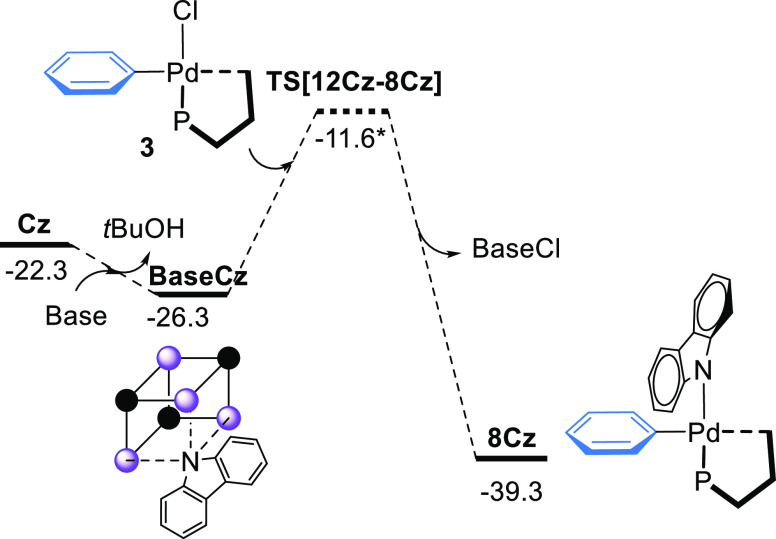
Gibbs energy profile for the formation of the **8Cz** complex. Gibbs energies are in kcal mol^–1^. *This value has been fitted using the microkinetic model.

The formation of the compound **8Cz** from **3**, carbazole, and NaO^*t*^Bu was studied by DFT calculations (Figure 9). Carbazole is deprotonated by the base forming species **BaseCz**, which is energetically favored by 4.0 kcal mol^–1^. This result is consistent with the acidic character of carbazole (p*K*_a_ = 19.9 in DMSO).^92^ The replacement of the chloride by the carbazolyl anion in **3** produces the intermediate **8Cz**, located at −39.3 kcal mol^–1^. The transition state of the chloride replacement has been set using the microkinetic model (*vide infra*).

**Scheme 8:**
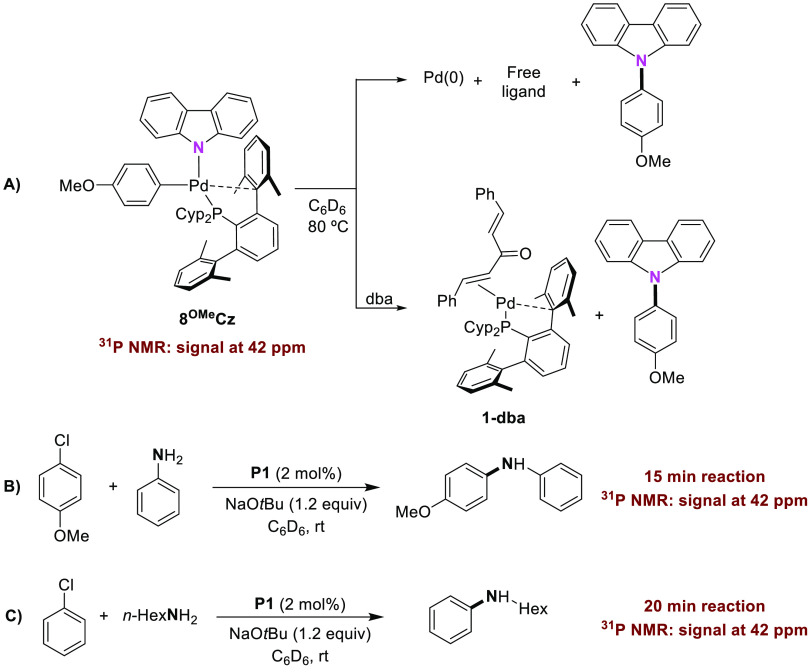
(A) Thermal Reductive Elimination of *N*-Arylcarbazole from **8^OMe^Cz**, (B) Cross-Coupling of 4-Chloroanisole with Aniline Using P1, and (C) Cross-Coupling of Chlorobenzene and *n*-Hexylamine Using **P1**

To shed light on the role of the aryl carbazolyl complex [(PCyp_2_Ar^Xyl2^)Pd(Ar)(carbazolyl)], **8Cz**, under catalytic conditions, a set of experiments was carried out. First, we studied the reductive elimination of *N*-arylcarbazole from the complex **8**^**OMe**^**Cz**. As summarized in Scheme 8A, the compound **8**^**OMe**^**Cz** underwent reductive elimination upon heating in C_6_D_6_ at 80 °C in 2 h, affording the corresponding C–N coupling product, free phosphine (observed by ^31^P NMR spectroscopy), and a black precipitate of Pd(0) (Figure S20). When accomplishing the reaction in the presence of 1.5 equiv of dba, the (PCyp_2_Ar^Xyl2^)Pd(0) species could be efficiently trapped as the dba adduct, **1-dba** (Figure S21). We found that the complex **8**^**OMe**^**Cz** experienced a faster reductive elimination compared to that of the analogous RuPhos complex, for which a half-life time of 91 min has been reported.^53^ Presumably, the bulkiness of the terphenyl phosphine ligand may account for such a difference.

Next, we monitored by ^31^P NMR spectroscopy the coupling between 4-chloroanisole and aniline in C_6_D_6_ at ambient temperature using 2.0 mol % of the precatalyst **P1** (Scheme 8B). Within 15 min of reaction, we observed a species whose ^31^P chemical shift (*ca.* 42 ppm) matched that of the Pd–carbazolyl complex, in addition to the free ligand and phosphine oxide (see Figure S22 for NMR details). The concentration of this species decreased as the reaction proceeded. A similar experiment carried out with *n*-hexylamine and chlorobenzene, at room temperature, revealed the presence of the Pd–carbazolyl complex as the major phosphorus-containing species after 20 min of reaction, which disappeared when the reaction reached completion after 5 days (Scheme 8C and Figure S23).

Collectively, these experiments suggest that the complex [(PCyp_2_Ar^Xyl2^)Pd(Ar)(carbazolyl)], **8Cz**, is the catalyst resting state. In support of this, using **8Cz** as the precatalyst for the coupling of chlorobenzene with either aniline or *n*-hexylamine gave the same outcome as the precatalyst **P1** under the same conditions (see Table 3 below).

**Table 3:**
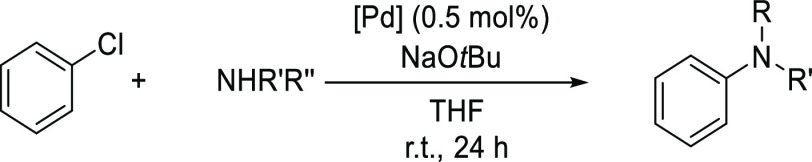

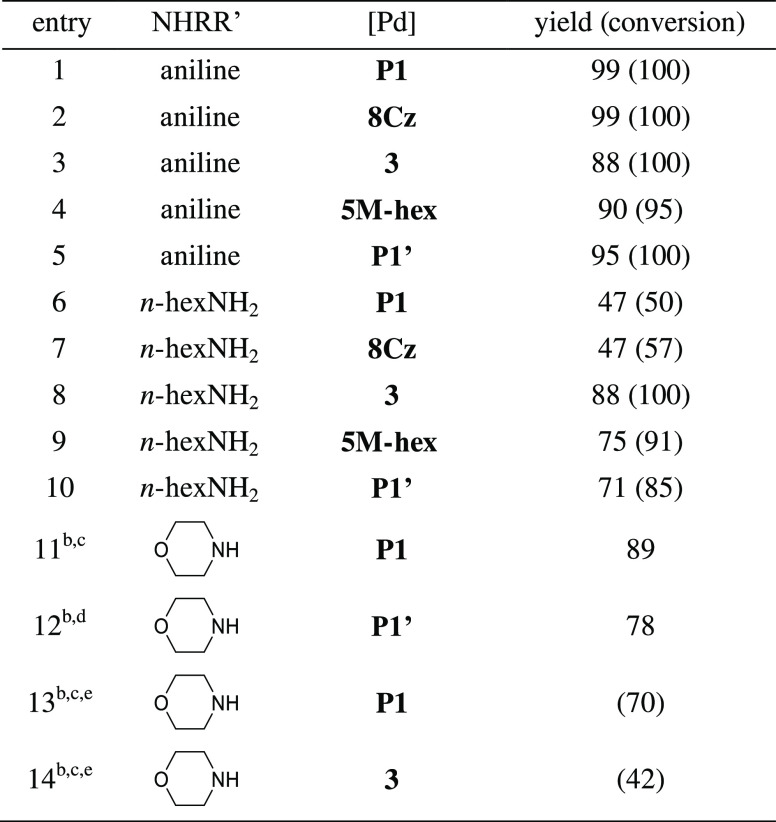
Catalytic Performance of Isolated Intermediates in the C–N Coupling of Chlorobenzene with Amines at Room Temperature^a^

a Reaction conditions: chlorobenzene (1 mmol), amine (1.2 mmol), [Pd] (0.5 mol%), NaO^*t*^Bu (1.2 mmol), THF (1 mL), 24 h (unoptimized); yields of isolated products (average of two runs). GC conversion in parenthesis.

b 4-chloroanisole (1 mmol) as aryl chloride.

c *T* = 80 °C.

d *T* = 110 °C.

e Reaction time: 4 h.

We also investigated the behavior of the carbazolyl complex **8Cz** toward the amine in the presence of the base at room temperature. Gas chromatography (GC) analysis of the reaction with aniline confirmed the presence of *N*-phenylcarbazole and diphenylamine. Conversely, when the experiment was carried out with *n*-hexylamine, only *N*-phenylcarbazole was detected by GC. These findings suggest that the carbazolyl ligand in **8Cz** could exchange with the anilide anion, establishing an equilibrium between **8Cz** and the anilide complex **8A**, favoring the former. Since *n*-hexylamine could not be deprotonated without the assistance of the Pd center, a similar equilibrium cannot be established.

### Mechanistic Proposal and Microkinetic Modeling

**Figure 10:**
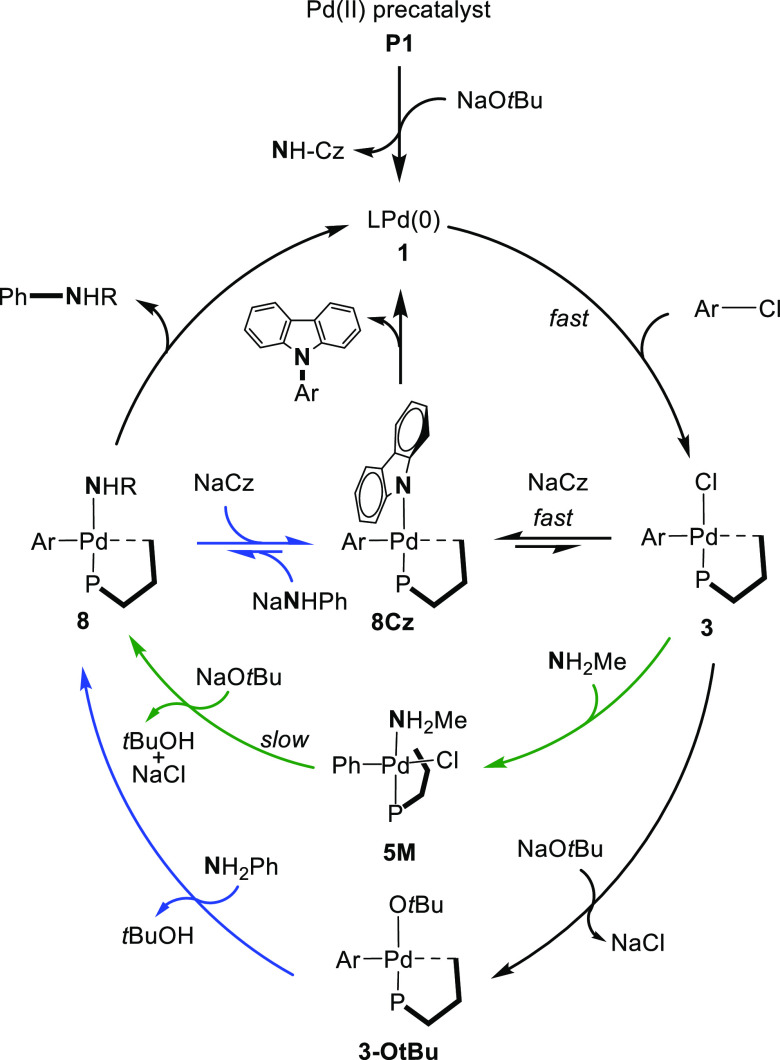
Proposed catalytic cycle for aniline (blue pathway) and methylamine (green pathway).

On the basis of computational and experimental data, the proposed mechanisms for the amination of the aryl chloride reaction catalyzed by palladacycle **P1** are shown in Figure 10.

For aromatic and aliphatic amines, precatalyst activation, oxidative addition, and reductive elimination are common steps. The activation of the palladacycle in the presence of the base is a highly exergonic and low-barrier step (Δ*G*_Act_ = −31.4 and Δ*G*_RE_^‡^ = 11.3 kcal mol^–1^). The activation generates a noninnocent byproduct, NH-carbazole, that, after deprotonation, coordinates to the oxidative addition product **3** producing a very stable aryl carbazolyl complex **8Cz**. The barrier for the reductive elimination of *N*-arylcarbazole (22.4 kcal mol^–1^) is the highest of all of the barriers computed for the different steps of the catalytic cycle. This species serves as the catalyst resting state, as inferred from NMR experiments as well as microkinetic analysis (*vide infra*). The existence of an equilibrium between the carbazolyl complex **8Cz** and the anilido analogue **8A** provides a faster route for the C–N coupling even at room temperature since the reductive elimination barrier from the latter is much smaller. However, such an equilibrium is not feasible for a more basic amine, such as methylamine (p*K*_a_*ca.* 42 in DMSO), which requires temperatures higher than the ambient temperature to facilitate the reductive elimination of *N*-arylcarbazole and the release of the catalytically active species.

Oxidative addition (Δ*G*^‡^ = 12.6 kcal mol^–1^) and reductive elimination (Δ*G*^‡^ = 13.6 kcal mol^–1^ for aniline and 8.1 kcal mol^–1^ for methylamine) steps have low energy barriers comparable to those found for the Pd catalyst systems bearing bulky, electron-rich ligands.^34−39^ For the ligand exchange step, two different pathways are found, which depend on the nucleophilicity and basicity of the amine employed. Due to the less nucleophilic character of aniline, the oxidative addition complex **3** reacts first with the base (^*t*^BuO^–^), leading to a neutral alkoxide intermediate **3-O*****^t^*****Bu**, from which the aryl amido intermediate **8A** is easily obtained. Concurrently, aniline could also be deprotonated without the assistance of the metal center, providing a more direct route to the intermediate **8A**. When a more nucleophilic amine is employed (*e.g*., primary alkylamine), amine coordination to oxidative addition complex **3** and intermolecular deprotonation by the base comprise the lower energy pathway to give **8M**.

Given that energy barriers found for most steps of the catalytic cycles could be surmounted at room temperature, we examined the C–N coupling of chlorobenzene with aniline and with *n*-hexylamine using the precatalyst **P1** at room temperature. While aniline provided quantitative yields of the diphenylamine product, *n*-hexylamine gave around 50% of the corresponding C–N coupling product (Table 3, entries 1 and 6). Moreover, identical results were obtained when the carbazolyl complex **8Cz** was used as the precatalyst (Table 3, entries 2 and 7). However, for the reaction with *n*-hexylamine, a notable improvement in yield was observed when on-cycle intermediates (oxidative addition product **3** and amino adduct **5M-Hex**) were tested as precatalysts (Table 3, entries 8 and 9). We also analyzed the room-temperature C–N couplings in the presence of palladacycle **P1′**, which generated *N*-methylcarbazole upon activation. The reaction with aniline produced identical results to that with palladacycle **P1** (entry 5), but with the alkylamine, the conversion and yield were akin to those obtained with on-cycle intermediates (entry 10). These results show that the carbazolyl species **8Cz** reduces the rate of the coupling reactions with alkylamines at room temperature, but it does not affect the rate of the coupling with aniline (Figure S24). Despite this, when testing the catalytic performance of precatalysts **P1**, **P1′**, and **3** in the thermal reaction between 4-chloroanisole and morpholine, palladacycle **P1** performed significantly better than **P1′** and outperformed **3** under the same reaction conditions (Table 3, entries 11–14). These findings suggest that the carbazolyl species **8Cz** could prevent fast deactivation of the catalytically active species, maintaining most palladium species within the productive part of the catalytic cycle.

To assess our mechanistic proposal and estimate missing energy barriers involving the base, we built a microkinetic model (see the Supporting Information for details). This technique allows one to simulate the evolution of the concentration of each species with time using rate constants provided by DFT calculations and initial concentrations provided by experiments.^93,94^ Microkinetic modeling offers a more realistic description of the catalytic system, and although it is widely used in heterogeneous catalysis, it has been scarcely applied to organometallic catalysis.^95−97^

**Figure 11:**
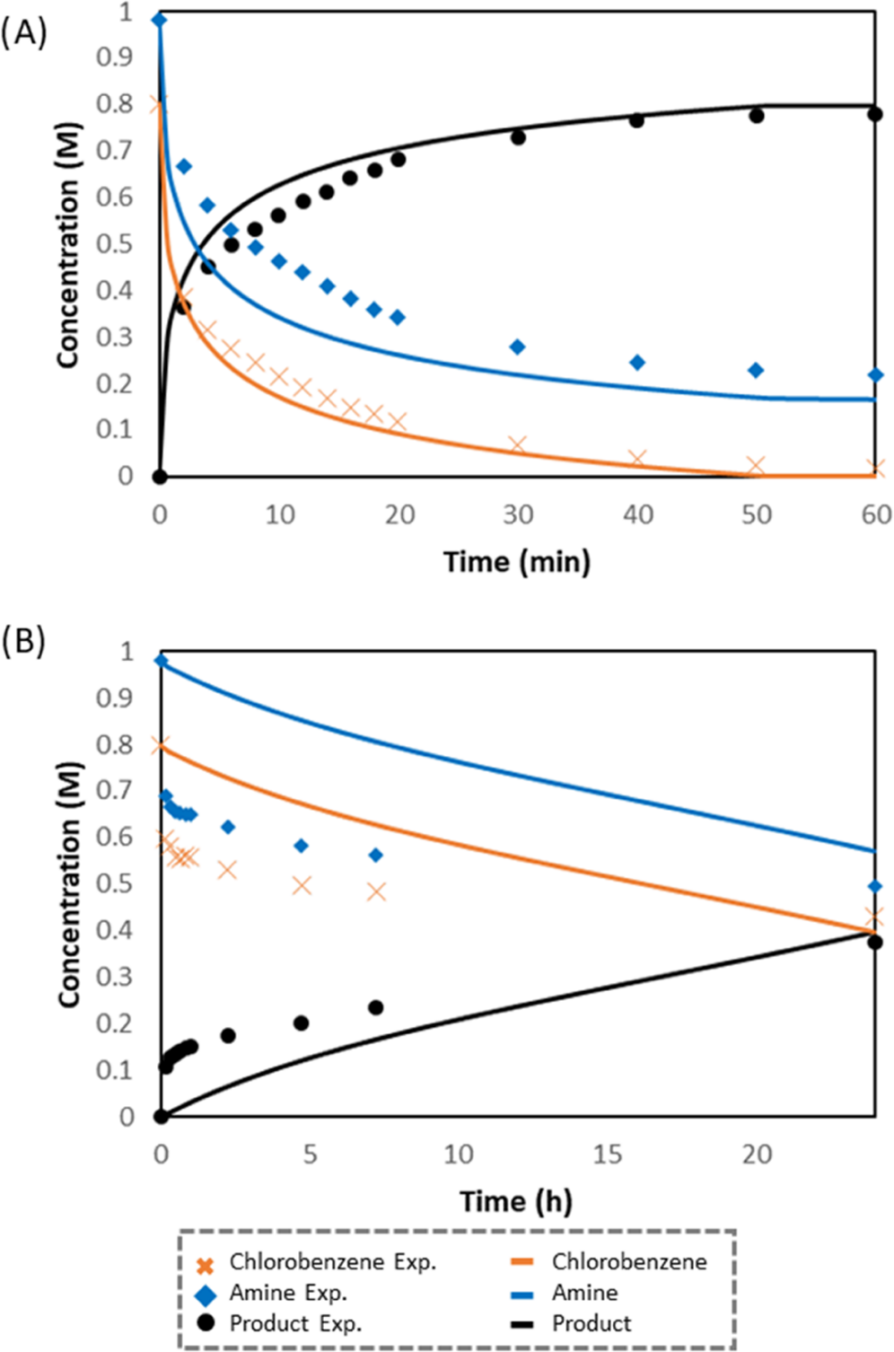
Comparison of experimental and modeled kinetic data for the C–N coupling of chlorobenzene with (A) aniline and (B) primary alkylamine (hexylamine for the experiments and methylamine for the calculations) at room temperature.

For the C–N coupling between chlorobenzene and aniline catalyzed by palladacycle **P1**, the model predicted a fast reaction at room temperature. As shown in Figure 11A, the microkinetic model reproduces satisfactorily the experimental results. The noncomputed barriers were adjusted to fit the shape of the experimental trend (see the Supporting Information for details).

However, when the microkinetic analysis was applied to the C–N coupling between chlorobenzene and methylamine, full conversion to *N*-methylaniline was reached in only 7 min at room temperature. This result clearly contrasts with the slow reaction observed at room temperature (Table 3, entry 6). To reproduce the experimental data, we evaluated the energy barriers for each of the individual steps of the catalytic cycle using the experimental information from the isolated reactions (see the Supporting Information). We found that the computed barrier for the reductive elimination of *N*-arylcarbazole from the intermediate **8Cz** (22.4 kcal mol^–1^) was underestimated by 2.0 kcal mol^–1^. Moreover, the barrier associated with the transition state for the deprotonation of the coordinated methylamine **5M-Base**, which could not be located, has to be adjusted to 21.0 kcal mol^–1^ (see Figure 7). With these optimized values, a good agreement between the model output and experimental kinetic data was obtained (Figure 11B). It is important to note that these fittings do not affect the results of the reaction with aniline. In addition, the microkinetic model predicted full conversions for the arylation of methylamine using complexes **3** and the amine adduct **5M-Hex** as precatalysts, in excellent agreement with those obtained in experiments depicted in Table 3 (entries 8 and 9).

The microkinetic analysis also showed that the carbazolyl complex **8Cz** was the catalyst resting state. Its concentration remains constant during the progress of the reaction with aniline. However, in the reaction with an alkylamine, **5M-base** is formed in the first stage of the reaction at a high concentration of amine. When 18% of the amine has reacted, the complex **8Cz** is the major Pd-containing species, and its concentration decreases during the course of the reaction due to the reductive elimination of *N*-phenylcarbazole. In contrast, complexes **3** and **5M-base** are the resting states of the catalyst in the absence of carbazole, indicating that the reaction with the amine or base is the rate-limiting step.

**Scheme 9:**
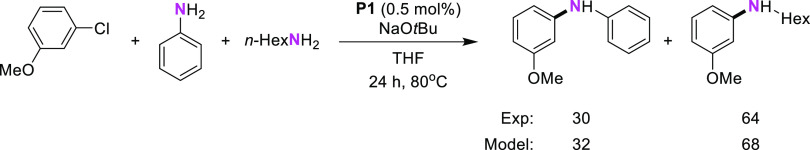
Amine Competition Experiments Using the Precatalyst **P1** Reaction conditions: 3-chloroanisole (0.5 mmol), *N*-nucleophile (0.6 mmol), [**P1**] (0.0025 mmol), NaO^*t*^Bu (0.6 mmol), THF (1 mL), 19 h. Conversions were determined by GC analysis of the reaction mixture using dodecane as the internal standard.

In short, the results provided by the microkinetic model validate the proposed reaction mechanism. Next, we tested the model to reproduce the selectivity observed in competition experiments between aniline and *n*-hexylamine. These experiments were conducted using 3-chloroanisole as the electrophilic coupling partner. Under the standard reaction conditions, there was a clear preference for the *N*-arylation of the primary alkylamine over the aromatic amine (Scheme 9).

To evaluate the selectivity, we prepared a model that combines the two types of mechanisms found for aniline and methylamine. With the adjustments made previously, the model provided an excellent agreement (see Scheme 9). The largest ratio of alkylamine is attributed to the fast accumulation of the **5M-base** intermediate, which is the major Pd-containing species formed in the first stage of the reaction with alkylamine.

## Conclusions

The overall catalytic cycle for the aryl amination reaction catalyzed by 2-aminobiphenyl palladacycle supported by terphenyl phosphine, PCyp_2_Ar^Xyl2^, **P1**, was analyzed in detail by computational and experimental methods. **P1** activation and ArCl oxidative addition, ligand exchange, and reductive elimination steps are all characterized by low activation barriers. However, the NH-carbazole byproduct liberated upon **P1** activation greatly influences the catalyst’s performance by forming a stable aryl carbazolyl Pd(II) intermediate. Such an intermediate serves as the catalyst resting state, releasing catalytically active monoligated LPd(0) species into the cycle upon reductive elimination of *N*-arylcarbazole. With less basic amines like aniline, fast reaction occurs at room temperature. The facile deprotonation of aniline enables an equilibrium between the aryl carbazolyl complex and the on-cycle anilido analogue, thus circumventing the higher activation barrier of *N*-arylcarbazole reductive elimination. Such an equilibrium is precluded with more basic primary alkylamines, which require heating to achieve an efficient transformation. A microkinetic model built with computed barriers and thermodynamics reproduced experimental data and selectivity, validating the proposed catalytic cycles. Furthermore, experimental data allowed estimating barriers that are difficult to calculate.

The results described in this work suggest that the NH-carbazole byproduct formed in reactions catalyzed by 2-aminobiphenyl palladacycles could decrease the reaction rate of some cross-couplings but stabilize the metal center at high temperatures. Furthermore, the stability and ease of tunability of the aryl carbazolyl Pd(II) intermediate open up its use as a precatalyst for cross-coupling reactions.

## Supporting Information Available

Computational methods and results; experimental procedures; NMR data, and crystallographic details (PDF)
**1-dba** (CIF)
**3CN** (CIF)
**8OMeCz** (CIF)
Optimized coordinates for calculated structures (XYZ)

## Supplementary Material

cs3c00075_si_001.pdf

cs3c00075_si_002.cif

cs3c00075_si_003.cif

cs3c00075_si_004.cif

cs3c00075_si_005.xyz

## Accession Codes

CCDC 2221856 (**1-dba**), 2224943 (**3CN**), and 2224944 (**8OMeCz**) contain the supporting crystallographic data for this paper. This data can be obtained free of charge *via*www.ccdc.cam.ac.uk/data_request/cif.
