# Supplementary material for: Important Role of NH-Carbazole in Aryl Amination Reactions Catalyzed by 2-Aminobiphenyl Palladacycles
Source: ACS Catal. 2023 Mar 7;13(6):3934–48. doi: 10.1021/acscatal.3c00075 (PMC10029719; doi:10.1021/acscatal.3c00075)
Supplement: Supplementary file 1 — cs3c00075_si_001.pdf [file cs3c00075_si_001.pdf]

Supporting Information

The Important Role of NH-Carbazole in Aryl Amination Reactions Catalyzed by 2-Aminobiphenyl Palladacycles

Raquel J. Rama,[†,‡] Celia Maya,[§] Francisco Molina,[#] Ainara Nova,[‡,*] M. Carmen Nicasio[†,*]

[†]Departamento de Química Inorgánica, Universidad de Sevilla, Aptdo 1203, 41071 Sevilla, Spain.

[‡]Department of Chemistry, Hylleraas Centre for Quantum Molecular Sciences and Centre for Materials Science and Nanotechnology, University of Oslo, N-0315 Oslo, Norway.

[§]Instituto de Investigaciones Químicas (IIQ), Departamento de Química Inorgánica and Centro de Innovación en Química Avanzada (ORFEO-CINQA), Consejo Superior de Investigaciones Científicas (CSIC) and Universidad de Sevilla, Avenida Américo Vespucio 49, 41092 Sevilla, Spain.

#Laboratorio de Catálisis Homogénea, Unidad Asociada al CSIC, CIQSO-Centro de Investigación en Química Sostenible and Departamento de Química, Universidad de Huelva, 21071 Huelva, Spain.

a.n.flores@kjemi.uio.no; mnicasio@us.es

Table of contents

1. Computational Details

Tetrameric Form of NaOtBu S3

Structure of palladacycle S4

Palladacycle activation S5

Coordination of solvent, amine or free phosphine to monoligated LPd(0) species S6

Oxidative addition S8

Ligand exchange S9

Reductive elimination S13

2. Experimental procedures and characterization data

General considerations S14

Activation of palladacycle with the base. S15

General procedure for the synthesis of [Pd(Ar)(Cl)(PCyp2ArXyl2)] complexes**, 3** S17

General procedure for the synthesis of [Pd(Ar)(carbazolyl)(PCyp2ArXyl2)] complexes, **8Cz** and **8OMeCz** S21

General procedure for the synthesis of [Pd(amine)(C6H5)(Cl)(PCyp2ArXyl2)] complexes S24
NMR study of reductive elimination from [Pd(4-OMe-C6H4)(carbazolyl)(PCyp2ArXyl2)], **8OMeCz** S26
Catalyst resting state S28
General catalytic procedure for testing the catalytic performance of isolated intermediates (Table 3). S29
Catalytic performance of precatalyst **P1** and on-cycle complex **3.** S29
3. Microkinetic modeling. S30
4. NMR spectra of compounds. S33
5. X-ray structural data of new complexes S48
6. References S52

1. Computational Details

DFT calculations were carried out with the Gaussian16 (Revision B.01) software package.[1] M06 functional[2] was used in both the geometry optimization with double-z basis set (def2SVP)[3,4] and energy refinements, with triple-z basis set (def2TZVP).[3] Once converged, the geometry optimizations were complemented with the analytic calculation of the frequencies with the double-z basis set (def2SVP). Geometries were fully optimized without any symmetry or geometry constrain. Vibrational frequencies were used to classify all stationary points as either minima (i.e. reactants, intermediates and products, with only real frequencies) or saddle points (i.e. transition states, with a single imaginary frequency vibrating along the reaction pathway connecting reactants to products). The solvent effects of THF were introduced at both the geometry optimizations and energy refinements using the continuum model SMD (Solvation Model based on Density).[5] The ultrafine pruned (99,590) grid was used in all calculations for higher accuracy. Gibbs energies in THF were calculated at 298K. A correction of +/-1.9 kcal·mol^{-1} was applied to the ΔG of reactions involving a change of molecularity for changing the standard state from the gas phase (1 bar) to solution (1M).[6]

1.1. Tetrameric Form of NaO*t*Bu

Sodium *tert*-butoxide has been widely used in Pd-catalyzed aryl amination reaction as non-nucleophilic base to accomplish the deprotonation step. Recent studies modeled the base NaO*t*Bu as a tetrameric cubane-type cluster (Figure S1) considering this was the most likely structure in a non-polar solvent environment (ε < 20).[7,8] A tetrameric geometry of alkali metal alkoxides was determined previously experimentally[9–11] and computationally.[7,8] Based on these works, it can be assumed that a tetrameric cubane-type cluster would be the most plausible arrangement in THF (ε = 7.58), the solvent employed in the catalytic reactions. Although we run the calculations using both the tetramer ($[NaOtBu]_4$) and the anionic form ($OtBu^-$) of the base, we found that the base modelled as a tetramer provided results more consistent with those obtained from experiments (see section 1.6).

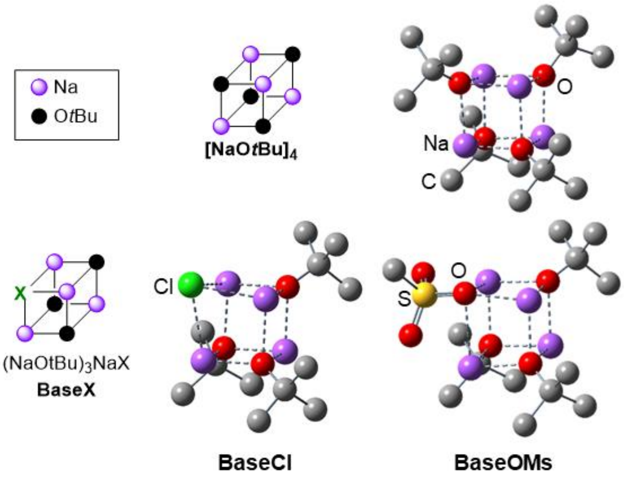

Figure S1. Schematic illustration and optimized structures of the tetrameric forms of [NaO*t*Bu]$_4$ (**Base**), (NaO*t*Bu)$_3$NaCl (**BaseCl**) and (NaO*t*Bu)$_3$NaOMs (**BaseOMs**). Hydrogen atoms have been omitted for clarity.

1.2. Structure of palladacycle

The energy of possible conformations of the precatalyst [Pd(2-aminobiphenyl)(PCyp$_2$Ar^{Xyl2})](OMs) in THF solution have been examined (Figure S2). We observed that the interaction between the coordinated NH group and the counterion, OMs$^-$, stabilized the geometry **P1** with respect to **P1-I** by 5.6 kcal·mol^{-1}. The coordination of OMs$^-$ to the Pd(II) center (**P1-II**) was disfavored by 3.1 kcal·mol^{-1} and the dissociation of the amino group (**P1-III**) by 4.9 kcal·mol^{-1}. The most stable conformation **P1** is in full agreement with the X-ray structure found for this compound.[12]

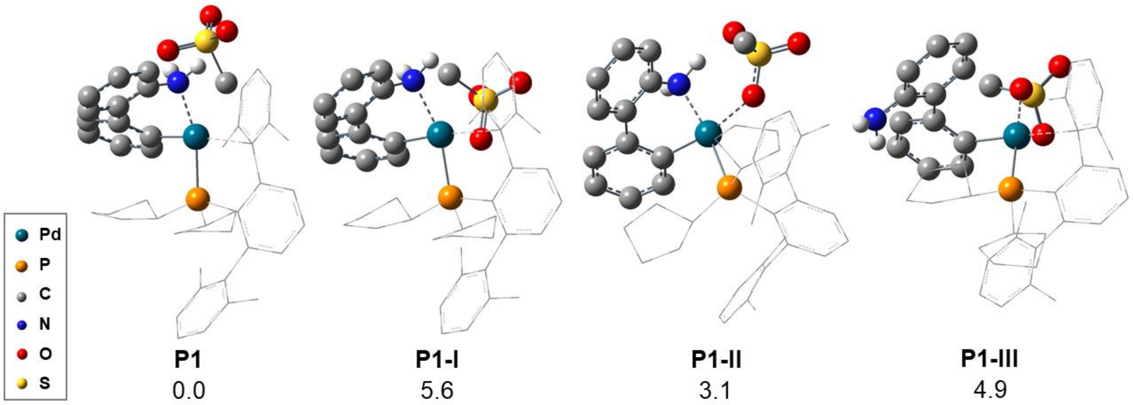

Figure S2. Relative free energies (in kcal·mol^{-1}) and optimized structures of possible conformations of the precatalyst [Pd(2-aminobiphenyl)(PCyp$_2$Ar^{Xyl2})](OMs). Hydrogen atoms (except NH) have been omitted for clarity.

1.3. Palladacycle activation

1.3.1. Base modelled as [NaO*t*Bu]$_4$ cluster

The palladacycle activation followed an associative mechanism involving the coordination of base to the Pd center via rotation of the terphenyl ring of the phosphine, and the formation of a (NaO*t*Bu)$_3$NaOMs cluster, **BaseOMs** (Figure 1). The coordination of the side ring of the phosphine was restored after intramolecular deprotonation and dissociation of *t*BuOH, giving the Pd(II)-amido intermediate **P4** at -12.1 kcal mol^{-1}. The energy barrier for the computed transition state is 10.1 kcal·mol^{-1} (Figure S3). The reductive elimination of carbazole released the monoligated LPd active species, **1**, with an energy barrier for the calculated transition state of 11.3 kcal·mol^{-1} (Figure S3).

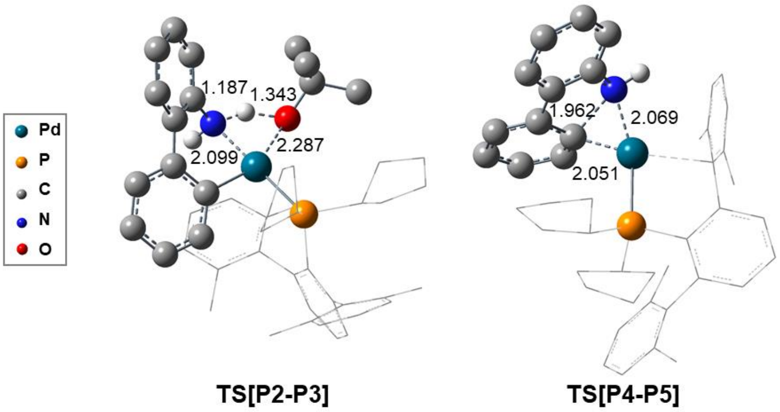

Figure S3. Optimized geometries for the transition states **TS[P2-P3]** (left) and **TS[P4-P5]** (right). Distances are in Å. Hydrogen atoms (except NH) have been omitted for clarity.

1.3.2. Base modelled as <u>*tert*</u>-butoxide anion

The associative mechanism for the palladacycle activation was also studied with the base modelled as <u>*tert*</u>-butoxide anion. In this case, the barriers associated with the computed transition states for the deprotonation of the amino group and the reductive elimination of carbazole were lower than that obtained in the previous case (5.4 and 12.2 kcal mol^{-1}, respectively), consistent with a rapid activation of the palladacycle (Figure S4).

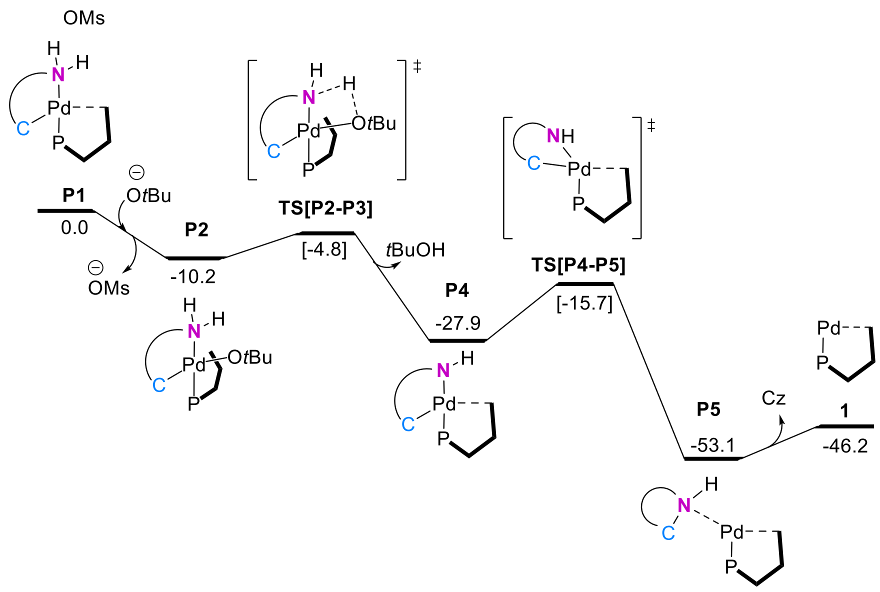

Figure S4. Activation of 2-aminobiphenyl-based palladacycles by the base NaO*t*Bu modelled as *tert*-butoxide anion.

In addition, we studied by DFT the intermolecular deprotonation of the amino group by the *tert*-butoxide anion. However, the transition state could not be located due to its low energy barrier.

In conclusion, computational results using different models for the alkoxide base showed that both intramolecular and intermolecular deprotonation mechanisms could occur simultaneously due to their low energy barriers. This is in agreement with the fast palladacycle activation observed experimentally.

1.4. Coordination of solvent, amine or free phosphine to monoligated LPd(0) species

The monoligated LPd(0) species may interact with the solvent and/or other molecules presented in the reaction mixture, stabilizing the low-coordinate metal center. Therefore, potential Pd(0) intermediates were considered in this study, such as $Pd(PCyp_2Ar^{Xyl2})_2$ (**1-L**), $Pd(PCyp_2Ar^{Xyl2})(THF)$ (**1-THF**) and $Pd(PCyp_2Ar^{Xyl2})(NHPh_2)$ (**8A**) (Figures S5-S6). The coordination of tetrahydrofuran had negligible influence in the stabilization of the monoligated species (3.7 kcal·mol^{-1}). Conversely, complex **1-L** was substantially more stable (-22.4 kcal·mol^{-1}). However, we could not detect the bis-ligated Pd(0) complex during catalysis, despite the presence of free phosphine ligand could be observed. Moreover, all attempts to isolate the bis-phosphine Pd(0) complex with the bulky $PCyp_2Ar^{Xyl2}$ were fruitless.[13]

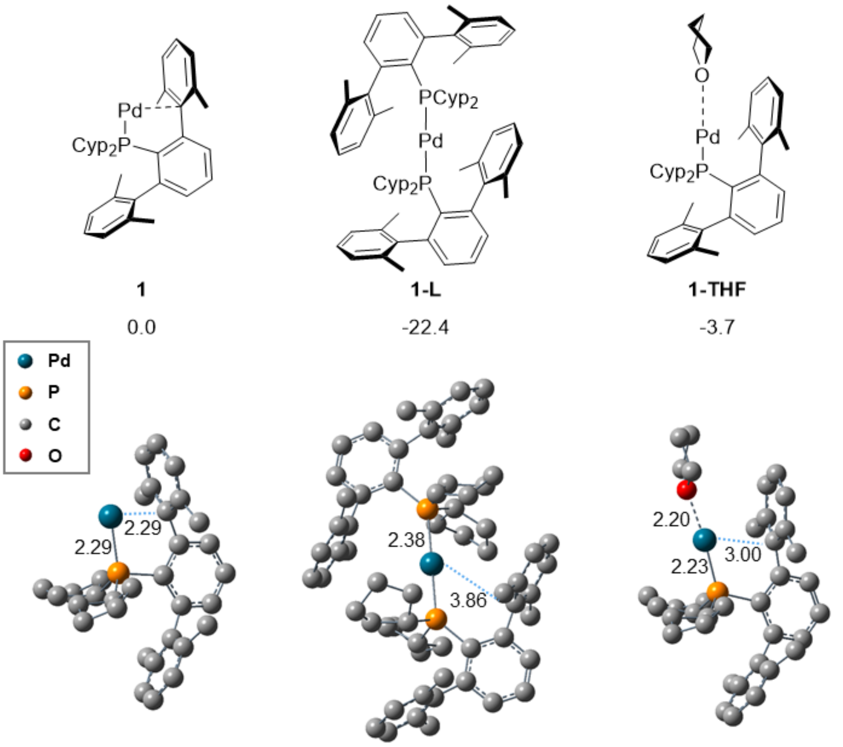

Figure S5. Relative free energies (in kcal·mol^{-1}) and optimized structures of **1**, **1-L** and **1-THF** complexes. Selected bond distances (in Å) are included. Hydrogen atoms have been omitted for clarity.

On the other hand, aryl amine coupling products could also stabilize LPd(0) species. Potential coordination modes of the nitrogen atom and the π-system of diphenylamine were studied. As shown in Figure S6, the species in which the amine is coordinated through the π system, **9A-II**, is nearly 6 kcal·mol^{-1} lower in energy than the complex in which the amine is coordinated through the N atom, **9A**.

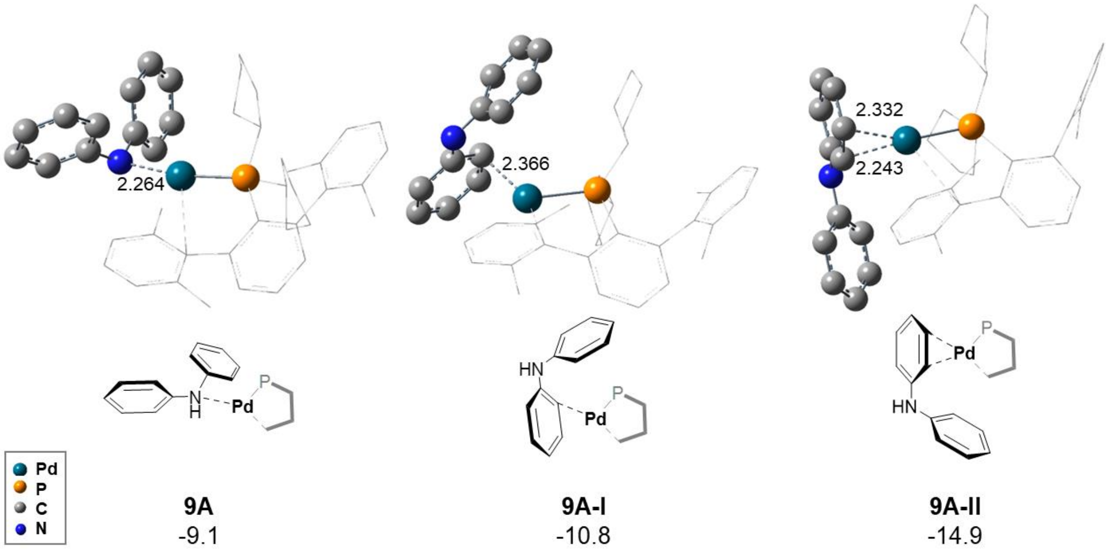

Figure S6. Relative free energies (in kcal·mol^{-1}) and optimized structures of species **9A**, **9A-I** and **9A-II**. Hydrogen atoms have been omitted for clarity.

1.5. Oxidative addition

Π-arene complexes with different *para*-substituted aryl chlorides were studied (R = H, OMe, CHO and CF_3), see Figure S7. The formation of complexes **2** was exothermic in all cases, regardless the coordination type and the substituents in the aryl moiety. As expected, Cl-C1 bonds were activated in conformation **I**, showing larger bond distances (Cl-C1: 1.77-1.78 Å) due to a closer interaction with the metal than in conformation **II** (Cl-C1: 1.75-1.76 Å). In addition, the orientation of the aryl chloride in conformation **I** was suitable for a concerted oxidative addition process. However, complexes containing C_{ortho}-C_{meta} (conformation **II**) interactions were more stable in all cases and, therefore, they were appointed as arene complexes **2**. The oxidative addition step was also studied with all the different substituents (Figure S8).

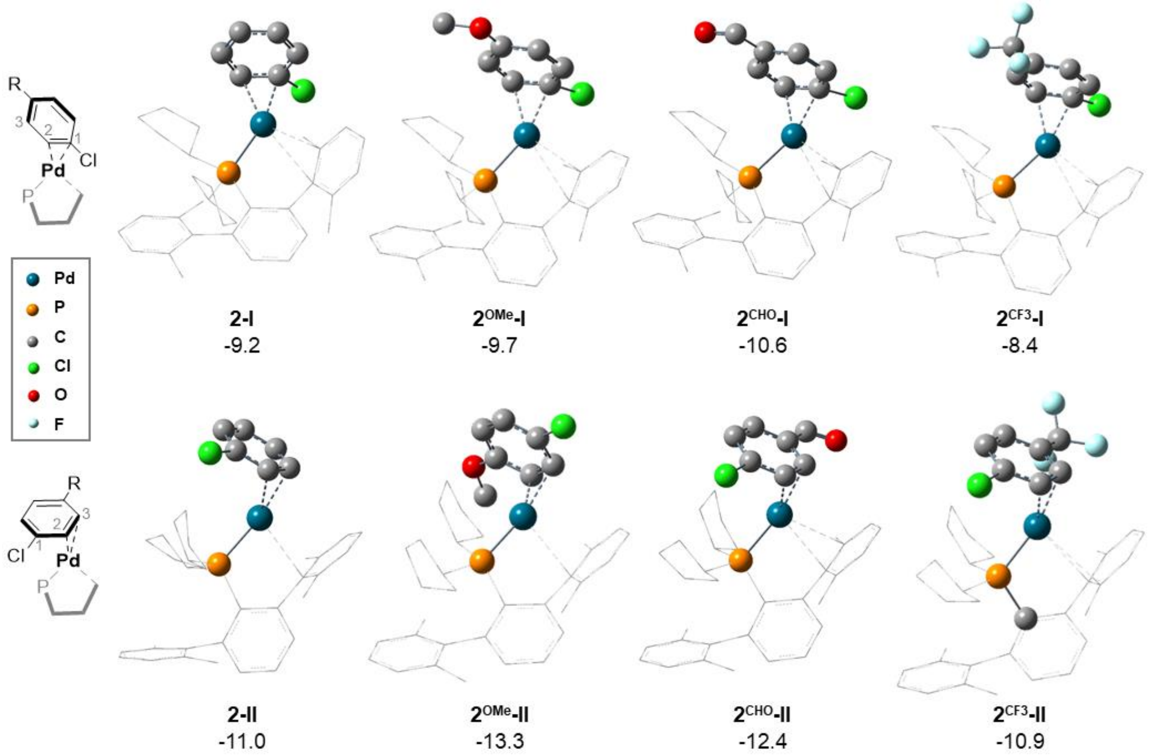

Figure S7. Relative free energies (in kcal·mol^{-1}) and optimized structures of species **2** in conformations **I** and **II**. Hydrogen atoms have been omitted for clarity.

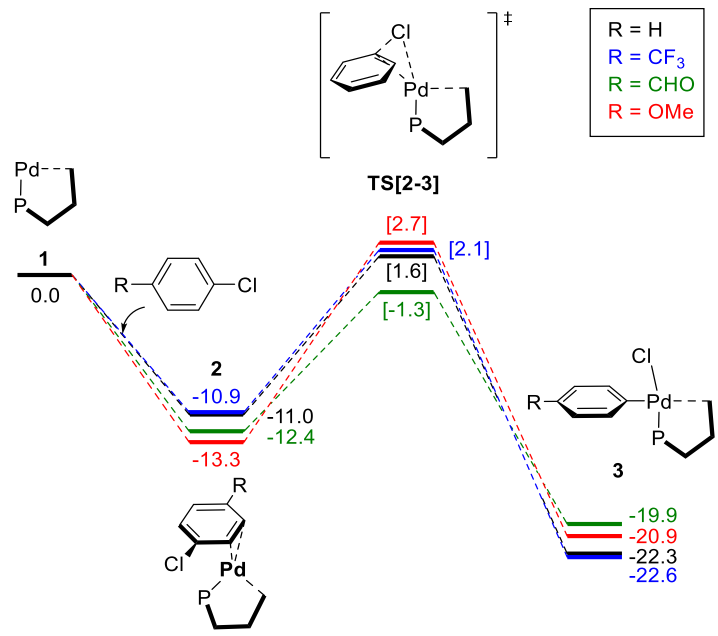

Figure S8. Gibbs energy profile for the oxidative addition step with different substituents in the *para*-position of the aryl chloride. Energies are in kcal·mol^{-1}.

1.6. Ligand exchange

We envisioned three different pathways for the reaction of intermediate **3** with the amine and the base (Scheme S1):

- Pathway I: chloride replacement by the amine forms cationic species **5-I**.

- Pathway II: the dissociation of the Pd-C$_{ipso}$ interaction produces two isomers (**4-II** and **4-II'**) with vacant sites in *cis* or *trans* position relative to the phosphine. Subsequent coordination of the amine originates intermediates **5-II** and **5-II'**, respectively.

- Pathway III: chloride replacement by *t*BuO$^-$ originates intermediates **3-OtBu**. As explained in pathway II, the formation of a vacant site in *cis* or *trans* position relative to the phosphine generates intermediates **4-III** and **4-III'** which, after amine coordination yield intermediates **5-III** and **5-III'**, respectively.

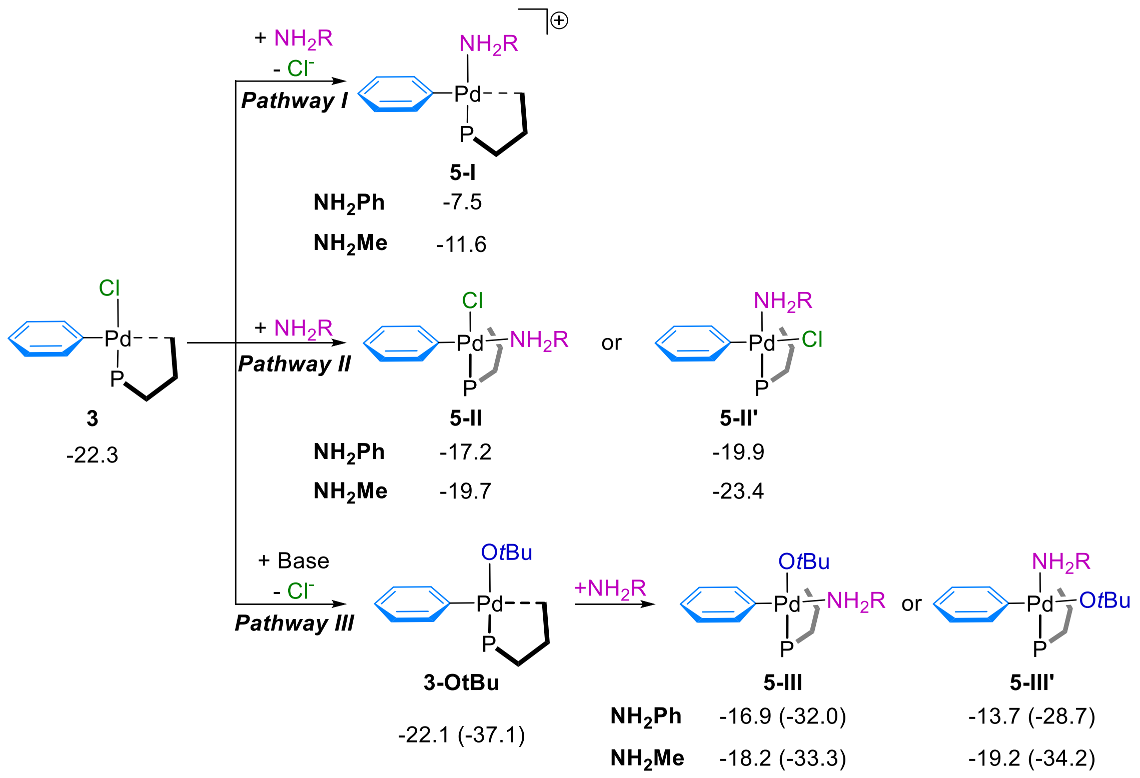

Scheme S1. Pathways for the reaction of intermediate **3** with the amine and the base. Gibbs energies are given in kcal·mol^{-1} (in parenthesis values for the base modelled as <u>*tert*</u>-butoxide anion).

As shown in Scheme S1, pathway I seems to be very unlikely since it requires the formation of a cationic species **5-I** in a non-polar solvent (e.g. THF). Species **5-I** containing aromatic and aliphatic amines are higher in energy than the intermediate **3** by 14.8 and 10.7 kcal·mol^{-1} respectively.

In pathway II, the coordination of the amine *trans* to the phosphine was preferred with both amines. However, the ordering of energies for isomers **5-III** and **5-III'** with aromatic amines was opposed due to the lower *trans* influence of the aromatic amine compared to methylamine and *t*BuO$^-$. Intermediates of pathways II and III were very close in energy, so neither of them could be ruled out from these results. In order to determine the preferred route, intermolecular and intramolecular deprotonations were studied.

Pathway III is highly influenced by whether we use the tetrameric form of NaO*t*Bu or *t*BuO$^-$ anion to model the base. When the base is modelled as *t*BuO$^-$ anion (see Figure S9-10), intermediate **3-OtBu** has a very low energy. In that case, pathway III would be the preferred one in both aromatic and aliphatic amines. Also, using the anionic model, species **3-OtBu** would be 6 kcal·mol^{-1} lower in energy than the carbazolyl complex **8Cz**.* However, **3-OtBu** has never been identified when monitoring the reaction experimentally. In addition, we observe that complex **3** reacts with aliphatic amines

forming complexes **5M-Morph** and **5M-Hex**, supporting a different mechanism than with aromatic amines. These results support the use of the tetrameric NaO*t*Bu cluster to model the base.

*Note: The synthesis of the carbazolyl complex **8Cz** from complex **3** is done in the presence of an excess of base, but we do not observe any other species.

1.6.1. Aromatic amines and the base modelled as <u>*tert*</u>-butoxide anion

To reduce computational costs, reaction pathway **III** (Scheme S1) was initially studied with the base defined as a <u>*tert*</u>-butoxide anion. The association of the base to complex **3** yielded the anionic intermediate **3-ClOtBu**, which upon dissociation of chloride gave complex **3-OtBu** (Figure S9). This process was energetically favorable by 14.8 kcal mol^{-1}. However, subsequent coordination of the amine was endergonic in either *cis* or *trans* positions relative to the phosphine ligand.

As explained above, the computed energies for isomers **5A** and **5A'** were in agreement with the expected *trans* influence, being the most favorable that containing the aniline *cis* to the phosphine (complex **5A**). Moreover, the route to form intermediate **5A** (species **5-II** in Scheme S1) was preferred since the formation of a vacant site through rotation of the phosphine was a barrierless process.

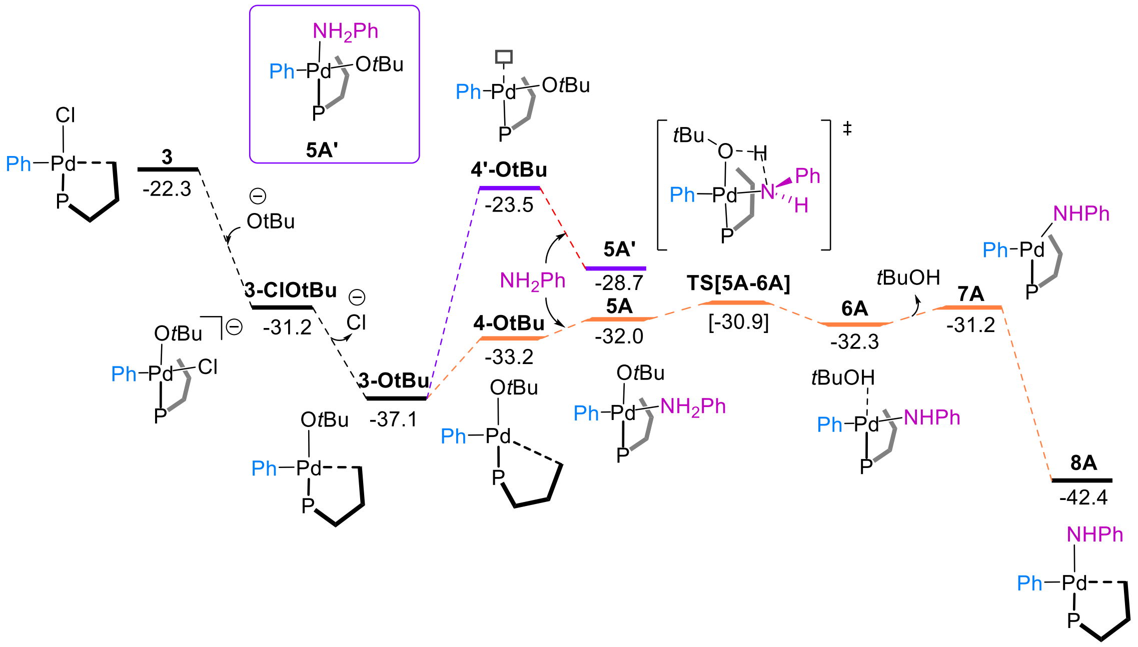

Figure S9. Gibbs energy profile for pathway **III** of the ligand exchange process with aniline and the base modelled in its anionic form. Gibbs energies are in kcal·mol^{-1}.

Deprotonation of coordinated amine and final dissociation of *t*BuOH generated the amido complex **8A** that was found to be 5.3 kcal mol^{-1} more stable than the intermediate **3-OtBu** (Figure S9). The Gibbs energy barrier for the coordination and deprotonation of aniline through pathway **III** was 6.2 kcal mol^{-1}.

1.6.2. Aliphatic amines and the base modelled as *tert*-butoxide anion

The study of reaction pathway **III** (Scheme S1) with methylamine and the base defined as a *tert*-butoxide anion is shown in Figure S10. As noted before, first steps of pathway **III** (formation of species **3-OtBu**, **4-OtBu** and **4'-OtBu**) were the same regardless of the amine used. After coordination of the methylamine in *cis* position relative to the phosphine ligand, intermediate **5M-III** was formed with a relative free energy of -33.3 kcal·mol^{-1}. The geometry of the transition state of the deprotonation in this case could not be obtained, but the product formed, **6M-III** (Figure S11), laid 23.1 kcal·mol^{-1} higher in energy than intermediate **3-OtBu**.

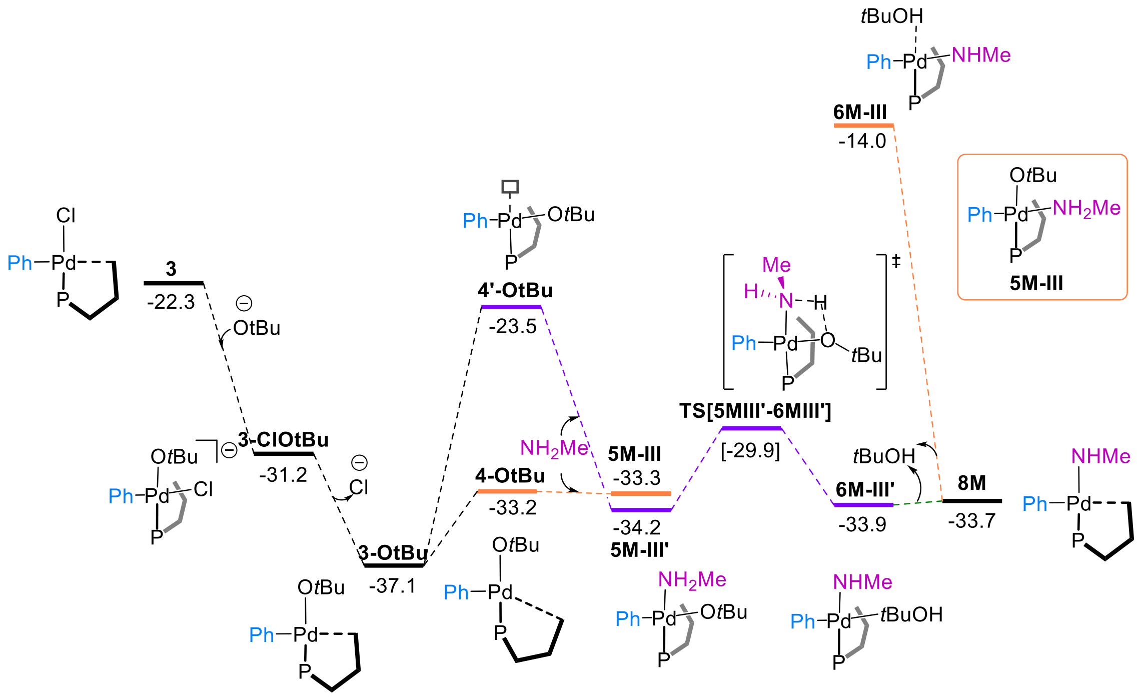

Figure S10. Gibbs energy profile for pathway **III** of the ligand exchange process with methylamine and the base modelled in its anionic form. Gibbs energies are in kcal mol^{-1}.

On the other side, when the methylamine was bonded to palladium in *trans* position relative to the phosphine, the calculated transition state for the deprotonation, **TS[5MIII'-6MIII']**, was 7.2 kcal·mol^{-1} higher in energy than intermediate **3-OtBu** (Figure S11). From those results and despite the formation of a vacancy site *trans* to the phosphine is less favorable, the preferred route of pathway III is that forming intermediate **5M-III'**.

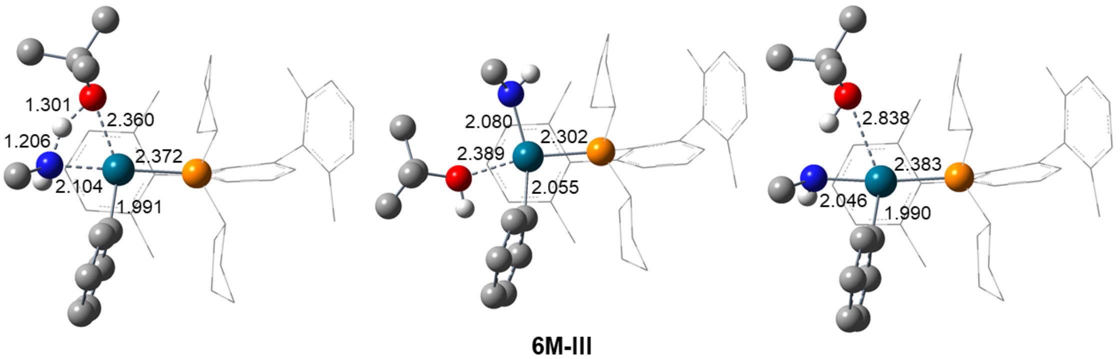

TS[5MIII-6MIII] **6M-III** **6M-III'**

Figure S11. Optimized geometries for the transition states **TS[5MIII-6MIII]** (left) and intermediates **6M-III** (middle) and **6M-III'** (right). Distances are in Å. Hydrogen atoms (except NH and OH) have been omitted for clarity.

1.7. Reductive elimination

1.7.1. Influence of electronic properties of aryl group

The electronic effects exerted by the aryl ligand in the reductive elimination step were evaluated using different *p*-substituted aryl halides (R = H, OMe, CF_3 and CHO) in the reaction with aniline. As shown in Figure S12, the reductive elimination barrier found for **8^{OMe}A** was only 0.7 kcal·mol^{-1} higher in energy than that found for the parent phenyl complex, **8A**. However, electron-withdrawing groups in the *para*-position of the aryl ligand facilitated the reductive elimination, as observed for **8^{CHO}A** and **8^{CF3}A** with barrier 4.1 and 2.2 kcal mol^{-1} lower in energy than the reference complex **8A**.

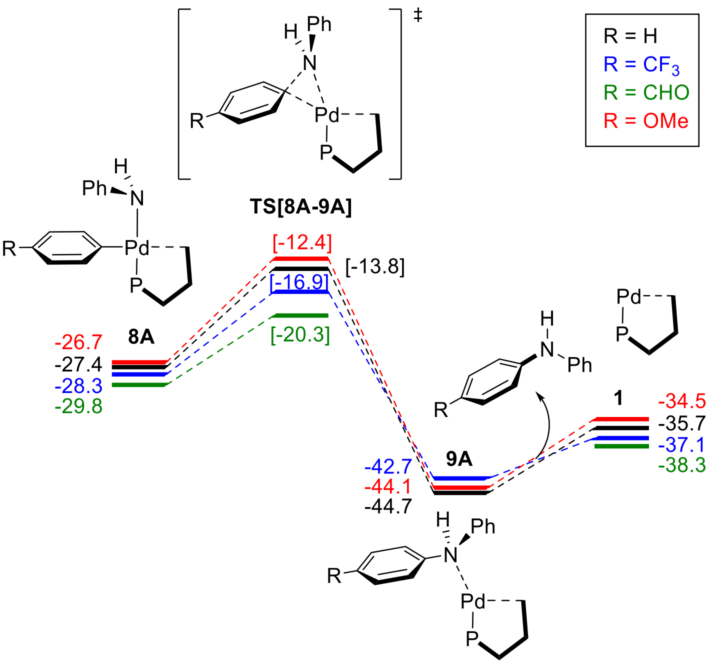

Figure S12. Gibbs energy profile for the reductive elimination step with different aryl ligands. Gibbs energies are in kcal·mol^{-1}.

1.7.2. Influence of electronic properties of amido group

The electronic effects of the amido ligand in the reductive elimination step were evaluated using primary amines (aniline and methylamine), secondary amines (dimethylamine and *N*-methylaniline) and an *N*-heterocycle (carbazole). Results obtained are shown in Figure S13.

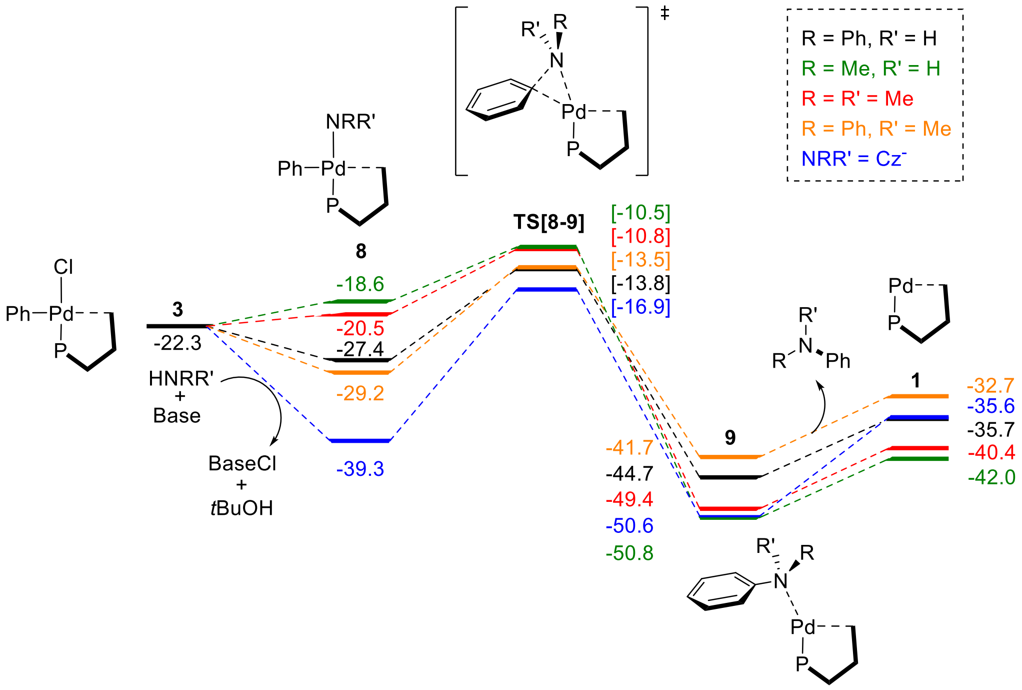

Figure S13. Gibbs energy profile for the reductive elimination step with different amido ligands. Gibbs energies are in kcal·mol^{-1}.

2. Experimental procedures and characterization data

2.1. General considerations

All preparations and manipulations were carried out under oxygen-free nitrogen atmosphere, using conventional Schlenk techniques. Palladacycles **P1**,[12] **P1'**[14] and complexes **3** and **3^{OMe}**[15] were synthesized following previously reported procedures. Reagents were purchased from commercial suppliers and used without further purification. Solvents were dried and degassed before use. Solution NMR spectra were recorded on a Bruker Avance III 500 MHz, Bruker Avance 300 MHz and 400 Ascend/R spectrometers. The ^{1}H and ^{13}C resonances of the solvent were used as the internal standard and the chemical shifts are reported relative to TMS while ^{31}P and ^{19}F were

referenced to external H_3PO_4 and $CFCl_3$, respectively. Elemental analyses were performed by the Servicio de Microanálisis of the Instituto de Investigaciones Químicas (IIQ). X-ray diffraction studies were accomplished at Centro de Investigación Tecnología e Innovación, CITIUS (Universidad de Sevilla), and Centro de Investigación en Química Sostenible, CIQSO (Universidad de Huelva).

2.2. Activation of palladacycle with the base.

Palladacycle **P1** (9 mg, 0.011 mmol) and NaO*t*Bu (2.5 mg, 0.026 mmol) were placed in an NMR tube under a nitrogen atmosphere. Degassed toluene-d_8 (0.5 mL) was added and the evolution of the reaction was monitored by ^{31}P and ^{1}H NMR spectroscopy at room temperature. After 1.5 h of reaction, free phosphine ligand together with phosphine oxide appeared as major species (Figure S14A). The presence of carbazole (Cz) and *t*BuOH was confirmed in the ^{1}H NMR spectrum (Figure S14B).

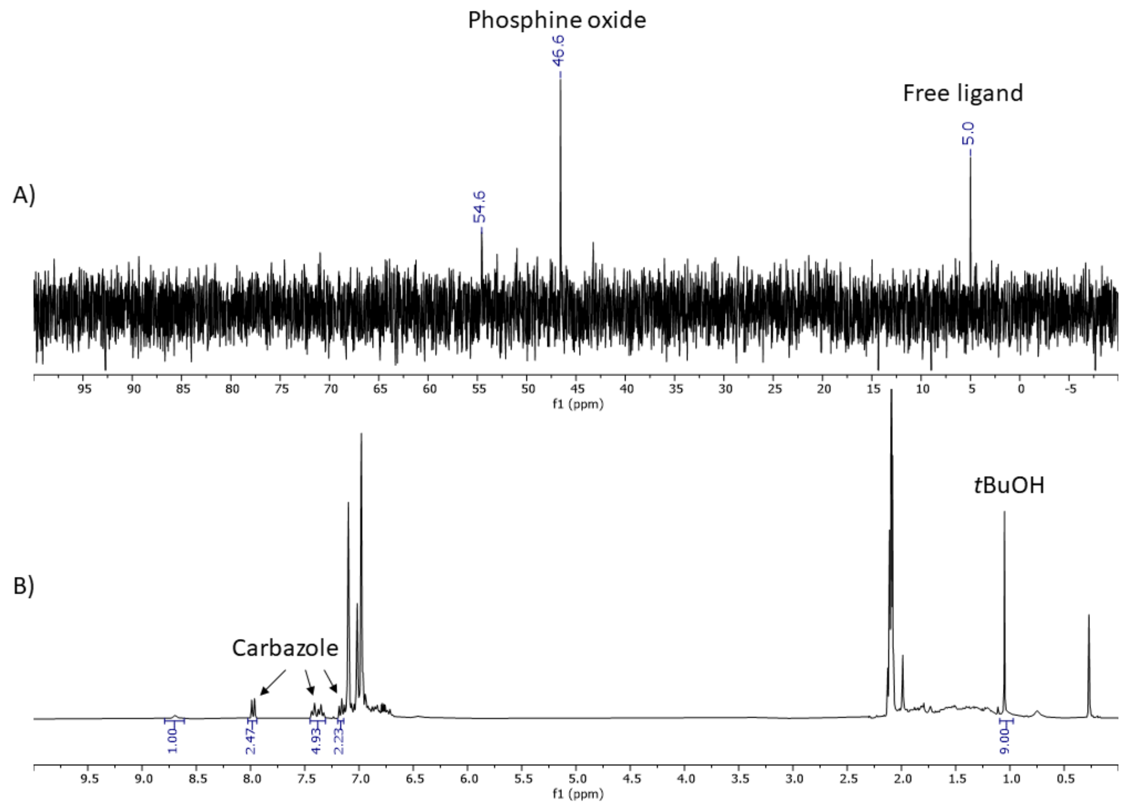

Figure S14. (A) ^{31}P NMR and (B) ^{1}H NMR spectra of the reaction between the **P1** and NaO*t*Bu at room temperature in toluene-d_8 after 1.5 hours.

2.2.1. Activation of palladacycle in the presence of dba. Synthesis of [Pd(η^2-dba)($PCyp_2Ar^{Xyl2}$)], 1-dba.

Activation of P1 in the presence of dba: A Schlenk tube equipped with a magnetic stir bar was charged with **P1** (14.8 mg, 0.018 mmol), dibenzylideneacetone (21.0 mg, 0.090 mmol) and NaO*t*Bu (3.5 mg, 0.036 mmol). Degassed diethyl ether was added (4 mL) and the mixture was stirred at room temperature for 2.5 hours. The reaction was diluted with Et_2O (5 mL) and filtered through a Celite plug. Pure product was obtained after recrystallization from a saturated diethyl ether solution at -20 °C.

General procedure for the synthesis of 1-dba: A solution of dibenzylideneacetone (23.4 mg, 0.1 mmol) and $PCyp_2Ar^{Xyl2}$ (45.5 mg, 0.1 mmol) in Et_2O (5 mL) was added to a solution of $Pd(CH_2SiMe_3)_2$(cod) (38.9 mg, 0.1 mmol) in Et_2O (3 mL) under nitrogen atmosphere. The reaction mixture was stirred for 2h at room temperature. The volatiles were removed under vacuum and the solid residue was dissolved in Et_2O (10 mL), filtered through a Celite plug and recrystallized from Et_2O : petroleum ether (3:1) at -20 °C. The title compound **1-dba** was obtained as orange crystals. Yield: 47.7 mg, 60 %.

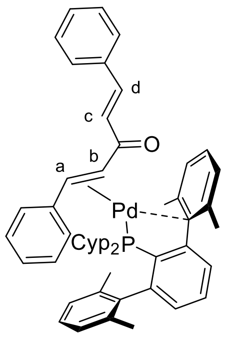

^1H NMR (500 MHz, CD_2Cl_2, 233 K): δ 7.73-6.96 (m, 18H, CH_{ar}), 6.79-6.74 (m, 2H, CH_{ar}), 6.46 (d, 1H, $^3J_{HH}$ = 15.6 Hz, H_c), 4.76 (dd, 1H, $^3J_{HH}$ = 11.1 Hz, $^3J_{HP}$ = 5.4 Hz, H_a), 2.99 (dd, 1H, $^3J_{HH}$ = 11.1 Hz, $^3J_{HP}$ = 6.3 Hz, H_b), 2.08-2.01 (m, 2H, Cyp), 1.99 (s, 3H, CH_3), 1.93 (s, 3H, CH_3), 1.86 (s, 3H, CH_3), 1.87-1.79 (m, 2H, Cyp), 1.74-1.68 (m, 2H, Cyp), 1.63 (s, 3H, CH_3), 1.56-1.46 (m, 4H, Cyp), 1.39-1.29 (m, 2H, Cyp), 1.06-0.95 (m, 2H, Cyp), 0.87-0.82 (m, 2H, Cyp), 0.66-0.59 (m, 1H, Cyp), 0.15-0.09 (m, 1H, Cyp).

$^{13}C\{^1H\}$ NMR (125 MHz, CD_2Cl_2, 233 K): δ 180.4 (d, J_{CP} = 3 Hz, *C*O), 149.5 (d, $^2J_{CP}$ = 33 Hz, *o*-C_6H_3), 146.1 (d, J_{CP} = 2 Hz, C_q), 144.6 (C_q), 140.3 (C_q), 138.1 (d, J_{CP} = 19 Hz, C_q), 137.5 (C_q), 136.5 (C_q), 136.1 (C_q), 135.2 (CH_{ar}), 132.3 (C_q), 131.4 (CH_{ar}), 130.7 (d, J_{CP} = 11 Hz, CH_{ar}), 130.3 (CH_{ar}), 129.0-128.1 (multiple overlapping peaks), 127.5 (CH_{ar}), 127.2 (CH_{ar}), 127.0 (CH_{ar}), 126.9 (CH_{ar}), 126.7 (CH_{ar}), 126.5 (CH_{ar}), 126.0 (d, J_{CP} = 6 Hz, CH_{ar}), 125.0 (CH_{ar}), 124.9 (CH_{ar}), 123.3 (C_c), 121.5 (CH_{ar}), 75.8 (d, $^2J_{CP}$ = 19 Hz, C_b), 66.6 (C_a), 38.0 (d, J_{CP} = 18 Hz, Cyp), 37.4 (d, J_{CP} = 18 Hz, Cyp), 35.9 (d, J_{CP} = 13 Hz, Cyp), 33.9 (d, J_{CP} = 16 Hz, Cyp), 30.6 (d, J_{CP} = 13 Hz, Cyp), 29.2 (d, J_{CP} = 8 Hz, Cyp), 25.8 (d, J_{CP} = 13 Hz, Cyp), 25.5 (d, J_{CP} = 10 Hz, Cyp), 23.8 (d, J_{CP} = 12 Hz, Cyp), 23.6 (d, J_{CP} = 15 Hz, Cyp), 22.9 (CH_3), 21.4 (CH_3), 20.9 (CH_3), 20.6 (CH_3).

$^{31}P\{^1H\}$ NMR (202 MHz, CD_2Cl_2, 233 K): δ 55.6.

Selected NMR data for the minor isomer:

^1H RMN (500 MHz, CD_2Cl_2, 233 K): δ 4.74-4.71 (m, 1H, H_a), 2.45 (dd, 1H, J_{HH} = 11.0 Hz, J_{HP} = 8.0 Hz, H_b).

$^{31}P\{^1H\}$ NMR (202 MHz, CD_2Cl_2, 233 K): δ 54.0.

Anal. Calc. for $C_{49}H_{53}OPPd$: C, 74.00; H, 6.72. Found: C, 74.20; H, 6.68.

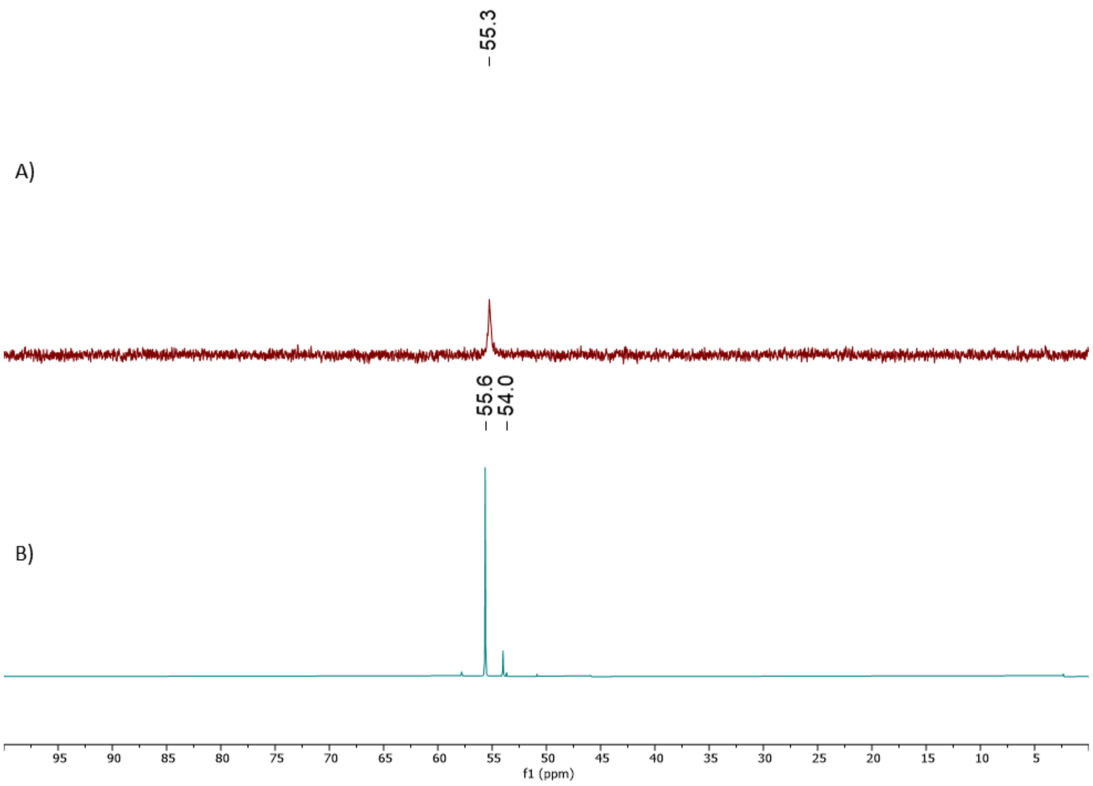

Figure S15. ^{31}P NMR of complex **1-dba** at (A) 298 K and (B) 233 K in CD_2Cl_2.

2.3. General procedure for the synthesis of [Pd(Ar)(Cl)($PCyp_2Ar^{Xyl2}$)] complexes, 3

A Schlenk tube, equipped with a magnetic stir bar, was charged with $PCyp_2Ar^{Xyl2}$ (68.2 mg, 0.15 mmol) and the aryl halide (0.45 mmol). The solids were dissolved in the minimum amount of hexane and then $Pd(CH_2SiMe_3)_2$(cod) (58.4 mg, 0.15 mmol) was added in one portion under a nitrogen flow. The reaction mixture was stirred overnight at room temperature. The solvent was removed under vacuum and the residue was washed

three times with pentane (3 x 4 mL) and dried under vacuum, providing the desired compound as off-white solids.

[Pd(4-CHO-C_6H_4)(Cl)($PCyp_2Ar^{Xyl2}$)], 3^{CHO}

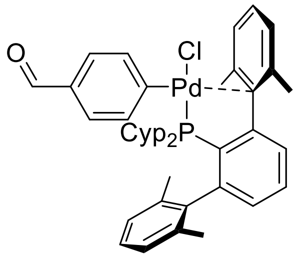

Following the general procedure, a mixture of Pd(CH_2SiMe_3)$_2$(cod) (58.4 mg, 0.15 mmol), $PCyp_2Ar^{Xyl2}$ (68.2 mg, 0.15 mmol) and 4-chlorobenzaldehyde (63.3 mg, 0.45 mmol) in hexane (15 mL) was stirred overnight. Yield: 55.0 mg (52%).

^{1}H NMR (300 MHz, $CDCl_3$, 298 K): δ 9.75 (s, 1H, C*H*O), 7.71 (br, 1H, *p*-Xyl), 7.48 (td, 1H, J_{HH} = 7.6 Hz, J_{HP} = 2.1 Hz, *p*-C_6H_3), 7.28 (d, 4H, J_{HH} = 1.5 Hz, CH_{ar}), 7.24-7.03 (m, 6H, CH_{ar}), 6.68 (br s, 1H, *m*-C_6H_3), 2.70-2.53 (m, 2H, C*H*Cyp), 2.38-2.15 (m, 2H, CH_2Cyp), 2.26 (s, 6H, CH_3), 2.03 (s, 6H, CH_3), 1.96-1.77 (m, 4H, CH_2Cyp), 1.55-1.42 (m, 4H, CH_2Cyp), 1.35-1.27 (m, 2H, CH_2Cyp), 1.06-0.94 (m, 2H, CH_2Cyp), 0.89-0.66 (m, 2H, CH_2Cyp).

^{13}C{^{1}H} NMR (75 MHz, $CDCl_3$, 298 K): δ 192.8 (*C*HO), 148.8 (d, J_{CP} = 1 Hz, C_q), 145.6 (C_q), 140.0 (C_q), 169.6 (C_q), 139.1 (d, J_{CP} = 4 Hz, CH_{ar}), 137.1 (C_q), 136.7 (C_q), 136.3 (C_q), 133.3 (CH_{ar}), 133.0 (CH_{ar}), 132.6 (CH_{ar}), 131.8 (d, J_{CP} = 2 Hz, CH_{ar}), 130.6 (CH_{ar}), 129.6 (C_q), 128.5 (CH_{ar}), 127.5 (CH_{ar}), 126.5 (d, J_{CP} = 2 Hz, CH_{ar}), 38.7 (d, J_{CP} = 27 Hz, *C*HCyp), 34.5 (d, J_{CP} = 9 Hz, *C*H_2Cyp), 29.5 (d, J_{CP} = 1 Hz, *C*H_2Cyp), 26.1 (*C*H_2Cyp), 25.9 (*C*H_2Cyp), 25.9 (*C*H_2Cyp), 25.7 (*C*H_2Cyp), 22.7 (*C*H_3), 21.1 (*C*H_3).

^{31}P{^{1}H} NMR (121 MHz, $CDCl_3$, 298 K): δ 46.4.

Elemental analysis calculated (found) for $C_{39}H_{44}ClOPPd\cdot 1/2\ CH_2Cl_2$: C, 63.76 (63.97); H, 6.10 (6.41).

[Pd(4-CF_3-C_6H_4)(Cl)($PCyp_2Ar^{Xyl2}$)], 3^{CF3}

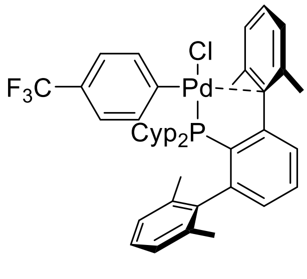

Following the general procedure, a mixture of Pd(CH_2SiMe_3)$_2$(cod) (58.4 mg, 0.15 mmol), $PCyp_2Ar^{Xyl2}$ (68.2 mg, 0.15 mmol) and 4-chlorobenzotrifluoride (60.0 µL, 0.45 mmol) in hexane (7 mL) was stirred overnight. Yield: 90.0 mg (81%).

^{1}H NMR (300 MHz, $CDCl_3$, 298 K): δ 7.70 (br s, 1H, *p*-Xyl), 7.47 (td, 1H, J_{HH} = 7.6 Hz, J_{HP} = 2.0 Hz, *p*-C_6H_3), 7.24-7.02 (m, 10H, CH_{ar}), 6.67 (br s, 1H, *m*-C_6H_3), 2.70-2.53 (m, 2H, Cyp), 2.36-2.18 (m, 2H, Cyp), 2.25 (s, 6H, CH_3), 2.03 (s, 6H, CH_3), 1.97-1.73 (m, 4H, Cyp,), 1.57-1.40 (m, 4H, Cyp), 1.35-1.23 (m, 2H, Cyp), 1.05-0.96 (m, 2H, Cyp), 0.77-0.60 (m, 2H, Cyp).

^{13}C{^{1}H} NMR (75 MHz, $CDCl_3$, 298 K): δ 148.8 (C_q), 145.6 (C_q), 140.9 (C_q), 139.9 (C_q),138.5 (CH_{ar}), 138.4 (CH_{ar}), 137.0 (C_q), 136.8 (C_q), 136.4 (C_q), 133.2 (CH_{ar}), 132.5 (CH_{ar}), 131.7 (d, J_{CP} = 2 Hz, CH_{ar}), 131.4 (CH_{ar}), 131.3 (CH_{ar}), 130.6 (CH_{ar}), 128.8 (C_q) 128.4 (CH_{ar}), 127.5 (CH_{ar}), 127.0 (d, J_{CP} = 8 Hz, C_q), 126.5 (C_q), 126.2 (d, J_{CP} = 32 Hz, C_q), 122.9 (C_q), 122.4 (d, J_{CP} = 2 Hz, CH_{ar}), 122.3 (d, J_{CP} = 2 Hz, CH_{ar}), 38.6 (d, J_{CP} = 27 Hz, *C*HCyp), 34.4 (d, J_{CP} = 9 Hz, *C*H_2Cyp), 29.4 (*C*H_2Cyp), 26.0 (*C*H_2Cyp), 25.8 (*C*H_2Cyp), 25.7 (*C*H_2Cyp), 22.7 (CH_3), 21.1 (CH_3).

^{31}P{^{1}H} NMR (121 MHz, $CDCl_3$, 298 K): δ 46.4.

^{19}F NMR (282 MHz, $CDCl_3$, 298 K): δ -61.9.

Elemental analysis calculated (found) for $C_{39}H_{43}ClF_3PPd$: C, 63.16 (63.20); H, 5.84 (6.08).

[Pd(4-CN-C_6H_4)(Cl)(PCyp$_2$Ar^{Xyl2})], 3^{CN}

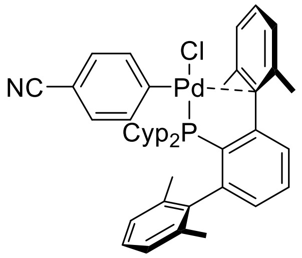

Following the general procedure, a mixture of Pd(CH_2SiMe_3)$_2$(cod) (58.4 mg, 0.15 mmol), PCyp$_2$Ar^{Xyl2} (68.2 mg, 0.15 mmol) and 4-chlorobenzonitrile (61.9 mg, 0.45 mmol) in hexane (10 mL) was stirred overnight. Yield: 75.5 mg (72%).

^{1}H NMR (400 MHz, $CDCl_3$, 263 K): δ 7.75 (t, 1H, $^{3}J_{HH}$ = 7.7 Hz, *p*-Xyl), 7.52 (t, 1H, $^{3}J_{HH}$ = 7.9 Hz, *p*-C_6H_3), 7.25-7.22 (m, 5H, CH_{ar}), 7.14-7.07(m, 5H, CH_{ar}), 6.70 (br d, 1H, $^{3}J_{HH}$ = 7.8 Hz, *m*-C_6H_3), 2.71-2.59 (m, 2H, C*H*Cyp), 2.32-2.17 (m, 2H, CH_2Cyp), 2.26 (br s, 6H, CH_3), 2.03 (br s, 6H, CH_3), 2.00-1.94 (m, 2H, CH_2Cyp), 1.85-1.80 (m, 2H, CH_2Cyp), 1.56-1.42 (m, 4H, CH_2Cyp), 1.39-1.32 (m, 2H, CH_2Cyp), 1.05-0.98 (m, 2H, CH_2Cyp), 0.71-0.61 (m, 2H, CH_2Cyp).

^{13}C{^{1}H} NMR (100 MHz, $CDCl_3$, 263 K): δ 148.4 (d, $^{2}J_{CP}$ = 22 Hz, *o*-C_6H_3), 145.3 (*ipso*-Ar), 145.1 (*o*-C_6H_3), 140.4 (*o*-Xyl), 139.1 (*ipso*-Xyl), 139.0 (*o*-Ar), 136.9 (*o*-Xyl), 136.6 (d, $^{1}J_{CP}$ = 34 Hz, *ipso*-C_6H_3), 133.4 (*m*-C_6H_3), 132.5 (d, $^{4}J_{CP}$ = 5 Hz, *p*-C_6H_3), 131.9 (*p*-Xyl), 130.2 (d, $^{3}J_{CP}$ = 12 Hz, *m*-C_6H_3), 130.4 (*m*-Xyl), 128.6 (*m*-Ar), 128.4 (*p*-Ar), 127.8 (d, $^{3}J_{CP}$

= 4 Hz, *ipso*-Xyl), 127.3 (*m*-Xyl), 120.0 (*C*N), 107.1 (*p*-Ar), 38.3 (d, $^{1}J_{CP}$ = 28 Hz, *C*HCyp), 34.5 (d, J_{CP} = 8 Hz, CH_2Cyp), 29.2 (CH_2Cyp), 26.1 (d, J_{CP} = 15 Hz, CH_2Cyp), 25.7 (d, J_{CP} = 10 Hz, CH_2Cyp), 22.8 (CH_3), 21.1 (CH_3).

$^{31}P\{^{1}H\}$ NMR (121 MHz, $CDCl_3$, 298 K): δ 46.7.

Anal. Calc. for $C_{39}H_{43}ClNPPd$: C, 67.05; H, 6.20; N, 2.00. Found: C, 67.21; H, 6.25; N, 2.12.

2.3.1. Reaction of palladacycle P1’ with the base in the presence of chlorobenzene.

P1’ (15.2 mg, 0.018 mmol), chlorobenzene (2 µL, 0.02 mmol) and NaO*t*Bu (2 mg, 0.02 mmol) were dissolved in C_6D_6 (0.5 mL) in an NMR tube under N_2 atmosphere. The reaction was monitored by ^{31}P and ^{1}H NMR spectroscopy at room temperature. After 30 min, palladacycle **P1’** was consumed and the oxidative addition complex **3** was obtained as a major product (Figure S16).

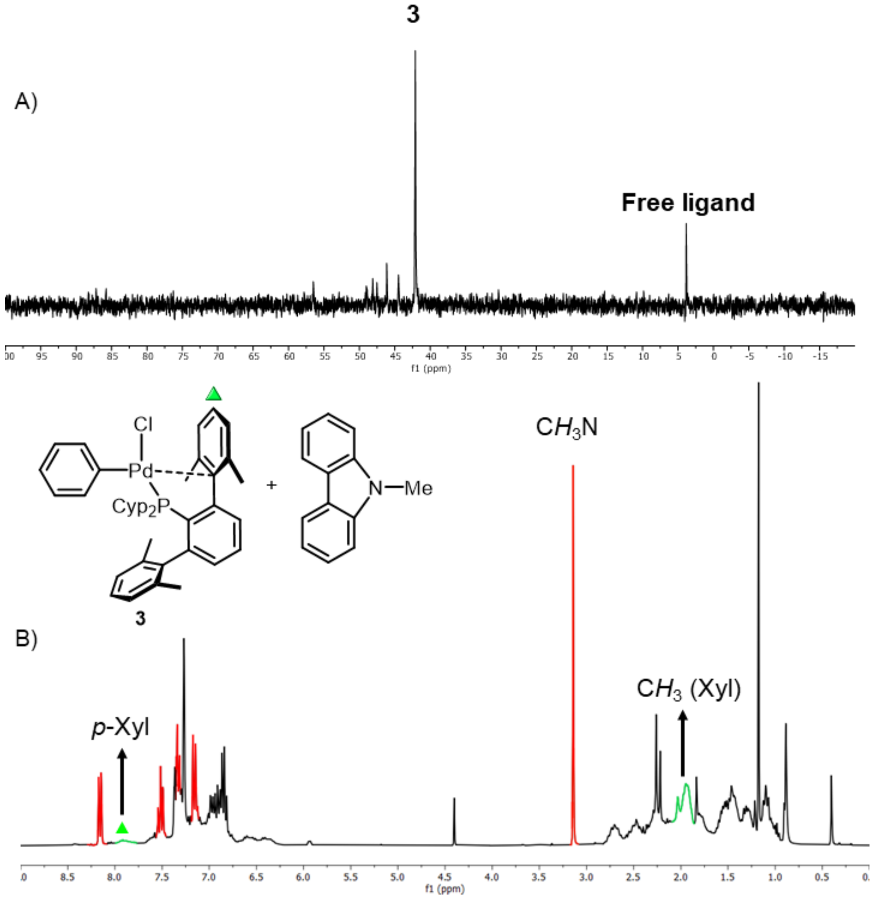

Figure S16. (A) ^{31}P NMR and (B) ^{1}H NMR spectra of the reaction of **P1’** with NaO*t*Bu in C_6D_6 after 30 min at room temperature.

2.3.2. Reaction of complex 3 with NaO*t*Bu

Figure S17 shows the ^{31}P NMR spectrum of the reaction of complex **3** (10 mg, 0.015 mmol) with NaO*t*Bu (14 mg, 0.15 mmol) in THF (1 mL) at room temperature after 30 min.

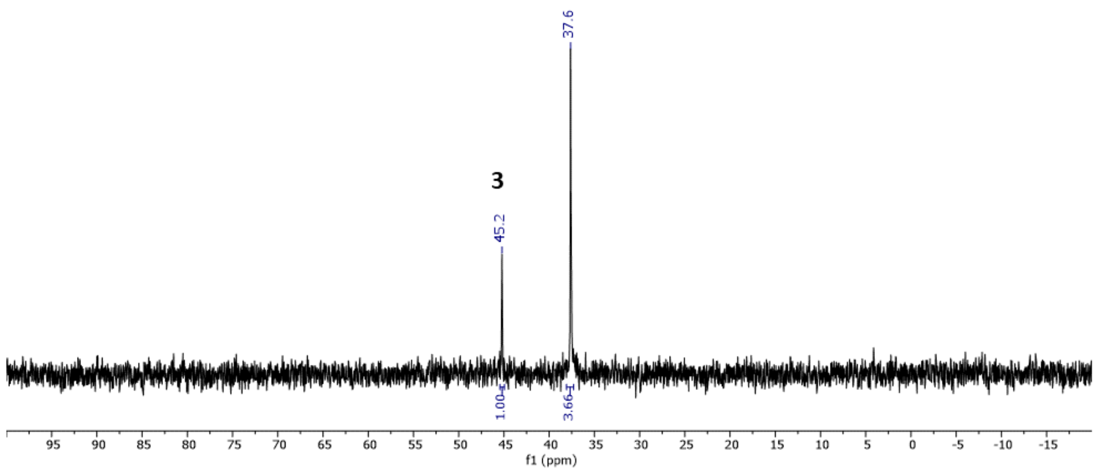

Figure S17. ^{31}P NMR spectrum of the reaction of **3** with NaO*t*Bu in THF after 30 min at room temperature.

2.4. General procedure for the synthesis of [Pd(Ar)(carbazolyl)(PCyp$_2$Ar^{Xyl2})] complexes, 8Cz and 8^{OMe}Cz

To a solution of carbazole (14.0 mg, 0.11 mmol) and NaO*t*Bu (96.1 mg, 1.00 mmol) in toluene (1 mL), a suspension containing [Pd(Ar)Cl(PCyp$_2$Ar^{Xyl2})] (0.10 mmol) in toluene (3 mL) was added. The reaction mixture was stirred for 2 hours at room temperature. The volatiles were removed under vacuum and the resulting solid residue was dissolved in Et_2O and filtered through a Celite plug. Complexes were purified by recrystallization from Et_2O : petroleum ether (3:1) mixtures at -20 °C.

[Pd(C_6H_5)(carbazolyl)(PCyp$_2$Ar^{Xyl2})], 8Cz.

Following the general procedure, the title compound was obtained as an orange solid. Yield: 28.5 mg, 70 %.

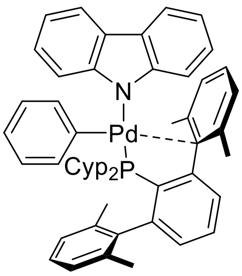

^{1}H NMR (300 MHz, $CDCl_3$, 298 K): δ 7.87 (d, 2H, $^3J_{HH}$ = 7.6 Hz, Cz), 7.48 (td, 1H, $^3J_{HH}$ = 7.5 Hz, $^5J_{HP}$ = 1.9 Hz, *p*-C_6H_3), 7.45 (d, 2H, $^3J_{HH}$ = 8.1 Hz, Cz), 7.33-7.22 (m, 3H, *p*-Xyl and Cz), 7.14 (d, 2H, $^3J_{HH}$ = 7.5 Hz, *m*-Xyl), 7.09 (br d, 1H, $^3J_{HH}$ = 7.5 Hz, *m*-C_6H_3), 7.03-7.00 (br, 2H, *m*-Ar), 6.91 (t, 2H, $^3J_{HH}$ = 7.2 Hz, Cz), 6.70 (br d, 1H, $^3J_{HH}$ = 7.5 Hz, *m*-C_6H_3), 6.57 (d, 2H, $^3J_{HH}$ = 7.7 Hz, *m*-Xyl), 6.38-6.36 (m, 3H, *o*-Ar and *p*-Ar), 5.61 (t, $^3J_{HH}$ = 7.6 Hz, *p*-Ar), 2.72-2.49 (m, 4H, Cyp), 2.46 (s, 6H, C*H*$_3$), 2.11 (s, 6H, C*H*$_3$), 2.05-1.95 (m, 2H, Cyp), 1.65-1.53 (m, 4H, Cyp), 1.42-1.30 (m, 2H, Cyp), 1.11-1.00 (m, 2H, Cyp), 0.94-0.85 (m, 2H, Cyp).

^{13}C{^{1}H} NMR (100 MHz, $CDCl_3$, 298 K): δ 149.0 (*o*-C_6H_3), 145.9 (*o*-C_6H_3), 140.2 (*ipso*-Xyl), 137.2, (C_q), 137.1 (*o*-Xyl), 136.6 (*m*-Ar), 132.4 (*m*-C_6H_3), 131.3 (*m*-C_6H_3),131.2 (*p*-C_6H_3), 131.1 (*p*-Xyl), 130.0 (*m*-Xyl), 128.3 (*p*-Xyl), 127.4 (*m*-Xyl), 125.3 (*p*-Ar), 123.3 (*p*-Ar), 122.5 (*C*HCz), 119.5 (C_q), 119.1 (*C*HCz), 115.4 (*C*HCz), 113.9 (*C*HCz), 38.3 (d, $^1J_{CP}$ = 26 Hz, *C*HCyp), 34.9 (d, J_{CP} = 10 Hz, *C*H$_2$Cyp), 29.3 (*C*H$_2$Cyp), 26.3 (d, J_{CP} = 14 Hz, *C*H$_2$Cyp), 25.6 (d, J_{CP} = 10 Hz, *C*H$_2$Cyp), 23.0 (*C*H$_3$), 21.2 (*C*H$_3$).

^{31}P{^{1}H} NMR (121 MHz, $CDCl_3$, 298 K): δ 42.6.

Anal. Calc. for $C_{50}H_{52}NPPd$: C, 74.66; H, 6.52; N, 1.74. Found: C, 74.50; H, 6.47; N, 1.55.

[Pd(4-OMe-C_6H_4)(carbazolyl)(PCyp$_2$Ar^{Xyl2})], 8^{OMe}Cz.

Following the general procedure, the title compound was obtained as orange solid by recrystallization. Yield: 34.2 mg, 82 %.

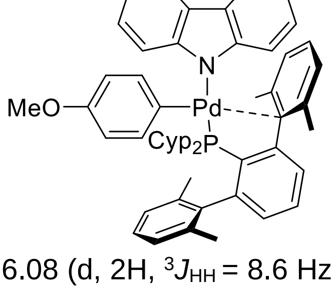

^{1}H NMR (300 MHz, C_6D_6, 298 K): δ 8.18 (d, 2H, $^3J_{HH}$ = 7.7 Hz, Cz), 7.84 (d, 2H, $^3J_{HH}$ = 8.0 Hz, Cz), 7.74-7.68 (m, 2H, Cz), 7.29-7.24 (m, 2H, Cz), 7.19-7.16 (m, 2H, *p*-Xyl and *m*-Ar), 6.97-6.88 (m, 2H, *p*-C_6H_3 and *m*-Ar), 6.83 (d, 2H, $^3J_{HH}$ = 7.5 Hz, *o*-Ar), 6.67 (d, 2H, $^3J_{HH}$ = 7.7 Hz, *m*-Xyl), 6.54 (dt, 1H, $^3J_{HH}$ = 7.4 Hz, $^4J_{HP}$ = 1.3 Hz, *m*-C_6H_3), 6.26-6.22 (m, 1H, *m*-C_6H_3), 6.08 (d, 2H, $^3J_{HH}$ = 8.6 Hz, *m*-Xyl), 5.71 (t, 1H, $^3J_{HH}$ = 7.7 Hz, *p*-Xyl), 2.77 (s, 3H, OC*H*$_3$), 2.71-2.55 (m, 4H, Cyp), 2.38 (s, 6H, C*H*$_3$), 2.10-2.02 (m, 2H, Cyp), 1.9-1.84 (m, 2H, Cyp), 1.87 (s, 6H, C*H*$_3$), 1.59-0.90 (m, 10H, Cyp).

^{13}C{^{1}H} NMR (100 MHz, C_6D_6, 298 K): δ 157.2 (*p*-Ar), 150.1 (*m*-C_6H_3), 149.6 (C_qCz), 145.8 (*m*-C_6H_3), 140.5 (*ipso*-Xyl), 138.5 (C_q), 137.1 (d, $^1J_{CP}$ = 29 Hz, *ipso*-C_6H_3), 137.1 (C_q), 136.8 (C_q), 136.5 (*p*-Xyl), 132.2 (*m*-C_6H_3), 131.8 (*p*-Xyl), 131.4 (d, $^3J_{CP}$ = 11 Hz, *m*-

C_6H_3), 131.4 (*p*-C_6H_3), 130.5 (*m*-Xyl), 130.1 (*ipso*-Ar), 128.0 (*m*-Ar), 127.4 (*o*-Ar), 126.5 (C_qCz), 124.5 (*ipso*-C_6H_3), 123.2 (*C*HCz), 120.3 (*C*HCz), 116.6 (C_q), 115.4 (*C*HCz), 115.2 (*C*HCz), 112.1 (*m*-Xyl), 53.9 (OCH_3), 38.6 (d, $^1J_{CP}$ = 26 Hz, *C*HCyp), 35.2 (d, J_{CP} = 10 Hz, CH_2Cyp), 29.6 (CH_2Cyp), 26.5 (d, J_{CP} = 14 Hz, CH_2Cyp), 25.9 (d, J_{CP} = 10 Hz, CH_2Cyp), 23.0 (CH_3), 21.1 (CH_3).

$^{31}P\{^1H\}$ NMR (121 MHz, C_6D_6, 298 K): δ 42.7.

Anal. Calc. for $C_{51}H_{54}$NOPPd: C, 73.41; H, 6.52; N, 1.68. Found: C, 73.29; H, 6.77; N, 1.50.

2.4.1. Reaction of palladacycle P1 with the base in the presence of 4-chloroanisole.

P1 (33.0 mg, 0.04 mmol), 4-chloroanisole (48.6 µL, 0.4 mmol) and NaO*t*Bu (46.1 mg, 0.48 mmol) were dissolved in toluene (5 mL) in a Schlenk tube under N_2 atmosphere. The reaction was stirred for 2 h at room temperature and monitored by ^{31}P NMR. The volatiles were removed under vacuum and the resulting solid residue was dissolved in Et_2O and filtered through a Celite plug. The complex was purified by recrystallization in a concentrated Et_2O solution. Pure product was obtained as orange crystals.

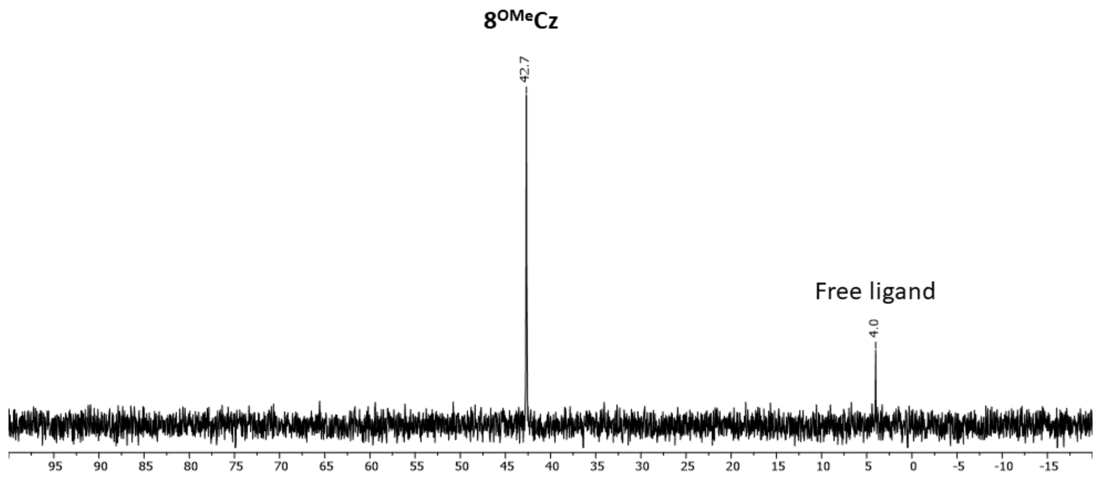

Figure S18. ^{31}P NMR spectrum of the reaction of **P1** with NaO*t*Bu in the presence of 4-chloroanisole after 2h at room temperature in toluene.

2.5. General procedure for the synthesis of [Pd(amine)(C_6H_5)(Cl)($PCyp_2Ar^{Xyl2}$)] complexes 5M

[Pd(Ph)(Cl)($PCyp_2Ar^{Xyl2}$)], **3**, (60 mg, 0.09 mmol), the amine (0.9 mmol) and toluene (4 mL) were added in turn to an Schlenk tube, equipped with a magnetic stir bar. The reaction mixture was stirred at room temperature for 30 minutes and then filtered through a Celite plug. After removing the solvent under vacuum, the crude solid was washed with hexanes (2 x 3 mL) and dried under reduced pressure to providing the desired compound.

[Pd(C_6H_5)(morpholine)(Cl)($PCyp_2Ar^{Xyl2}$)], 5M-Morph

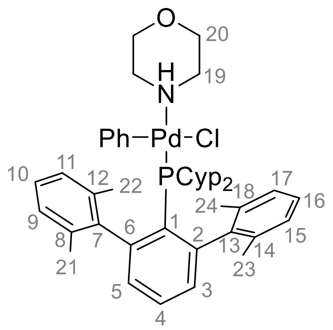

Following the general procedure, the title compound was obtained as a pale yellow solid. Yield: 45 mg (65%).

^1H NMR (500 MHz, tol-d_8, 243 K): δ 7.44 (d, 1H, J_{HH} = 5.5 Hz, *o*-Ph), 7.34 (t, 1H, J_{HH} = 7.5 Hz, H^{10}), 7.25 (d, 1H, J_{HH} = 7.6 Hz, H^9), 7.15 (d, 1H, J_{HH} = 7.7 Hz, H^{11}), 7.04 (t, 1H, J_{HH} = 7.5 Hz, H^{16}), 6.99-6.90 (m, 5H, H^4, H^{17}, *m*-Ph and *p*-Ph), 6.88 (d, 1H, J_{HH} = 7.6 Hz, H^{15}), 6.63 (ddd, 1H, J_{HH} = 7.6, 1.7 Hz, J_{HP} = 3.5 Hz, H^5), 6.49 (dt, 1H, J_{HH} = 7.6, 1.8 Hz, J_{HP} = 1.8 Hz, H^3), 5.31 (d, 1H, J_{HH} = 7.2 Hz, *o*-Ph), 3.73-3.67 (m, 1H, CH_2Cyp), 3.21 (d, 1H, J_{HH} = 11.0 Hz, H^{20}), 3.13 (br t, 1H, N*H*), 3.06-3.02 (m, 2H, H^{20} and C*H*Cyp), 3.01 (s, 3H, CH_3), 2.96 (d, 1H, J_{HH} = 13.0 Hz, H^{19}), 2.90 (s, 3H, CH_3), 2.89-2.85 (m, 1H, H^{19}), 2.84-2.71 (m, 2H, H^{20}), 2.50-2.40 (m, 1H, CH_2Cyp), 2.35 (d, 1H, J_{HH} = 13.0 Hz, H^{19}), 2.31-2.26 (m, 1H, C*H*Cyp), 2.13-2.10 (m, 1H, H^{19}), 2.06 (s, 3H, CH_3), 1.93 (s, 3H, CH_3), 1.76-0.93 (m, 12H, CH_2Cyp), 0.41-0.36 (m, 1H, CH_2Cyp), -0.16-(-0.24) (m, 1H, CH_2Cyp).

$^{13}C\{^1H\}$ NMR (125 MHz, tol-d_8, 243 K): δ 155.8 (d, J_{CP} = 2 Hz, C_q), 147.8 (d, J_{CP} = 17 Hz, C_q), 145.4 (C_q), 145.0 (d, J_{CP} = 5 Hz, C_q), 143.7 (C_q), 140.2 (C_q), 139.0 (C_q), 137.8 (C_q), 137.0 (C_q), 136.3 (d, J_{CP} = 5 Hz, *o*-Ph), 135.3 (*o*-Ph), 134.6 (d, J_{CP} = 31 Hz, C^1), 132.6 (d, J_{CP} = 6 Hz, C^3), 132.4 (d, J_{CP} = 9 Hz, C^5), 129.4 (CH_{ar}), 129.1 (CH_{ar}), 128.3 (C^{11}), 128.2 (C^{10}), 128.0 (C^9), 127.9 (CH_{ar}), 127.5 (C^{16}), 127.2 (CH_{ar}), 126.4 (CH_{ar}), 123.0 (CH_{ar}), 67.3 (C^{20}), 67.1 (C^{20}), 48.3 (C^{19}), 46.9 (C^{19}), 41.1 (d, J_{CP} = 22 Hz, *C*HCyp), 39.1 (d, J_{CP} = 15 Hz, CH_2Cyp), 38.6 (d, J_{CP} = 25 Hz, *C*HCyp), 33.1 (d, J_{CP} = 7 Hz, CH_2Cyp), 29.3 (d, J_{CP} = 4 Hz, CH_2Cyp), 27.6 (d, J_{CP} = 9 Hz, CH_2Cyp), 26.8 (d, J_{CP} = 9 Hz, CH_2Cyp), 26.4 (CH_2Cyp), 26.1 (d, J_{CP} = 8 Hz, CH_2Cyp), 25.7 (C^{22}), 24.3 (C^{24}), 23.7 (d, J_{CP} = 17 Hz, CH_2Cyp), 22.1 (C^{23}), 21.0 (C^{21}).

$^{31}P\{^1H\}$ NMR (202 MHz, tol-d$_8$, 243 K): δ 28.7.

Elemental analysis calculated (found) for $C_{42}H_{53}ClNOPPd$: C, 66.31 (66.61); H, 7.02 (6.96); N 1.84 (1.99).

[Pd(C$_6$H$_5$)(*n*-Hex)(Cl)(PCyp$_2$Ar^{Xyl2})], 5M-Hex

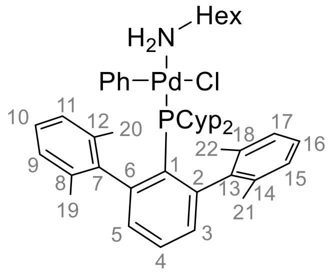

Following the general procedure, the titled compound was obtained as a white solid. Yield: 45 mg (65%).

^1H NMR (500 MHz, tol-d$_8$, 243 K): δ 7.48-7.35 (m, 4H, CH_{ar}), 7.07-6.97 (m, 7H, CH_{ar}), 6.73 (br d, 1H, H^5), 6.61 (d, 1H, J_{HH} = 7.1 Hz, H^3), 5.50 (s, 1H, *o*-Ph), 3.91 (s, 1H, CH_2Cyp), 3.15 (s, 3H, CH_3), 3.14-3.07 (m, 1H, Cyp/Hex), 3.01 (s, 3H, CH_3), 2.71-2.35 (m, 6H, Cyp and Hex), 2.23 (s, 3H, CH_3), 2.08 (s, 3H, CH_3), 1.84-1.31 (m, 15H, Cyp and Hex), 1.14-1.06 (m, 4H, Cyp and Hex), 0.79-0.65 (m, 5H, Cyp and Hex), -0.05 (br s, 1H, CH_2Cyp).

^{13}C NMR spectrum could not be recorded due to solubility problems together with the existence of the equilibrium between the amine complex and **3**, even a low temperature (see below).

$^{31}P\{^1H\}$ NMR (202 MHz, tol-d$_8$, 243 K): δ 27.4.

Elemental analysis calculated (found) for $C_{44}H_{59}ClNPPd$: C, 68.21 (68.12); H, 7.68 (7.52); N 1.81 (2.04).

Determination of the stereochemistry of [Pd(C$_6$H$_5$)(morpholine)(Cl)(PCyp$_2$Ar^{Xyl2})]

Figure S19 shows the NOESY experiment of a selected region of the complex **5M-Morph** at 243 K. NOE cross-peaks were found between an *ortho*-proton of the phenyl ligand and a morpholine proton (H^{19}) and between the first and a proton of the cyclopentyl group of the phosphine. This observation indicated that the phenyl ring is located close in space to both the phosphine and the morpholine ligands. From this experiment, it could be drawn that the amine is coordinated in *trans*-position relative to the phosphine.

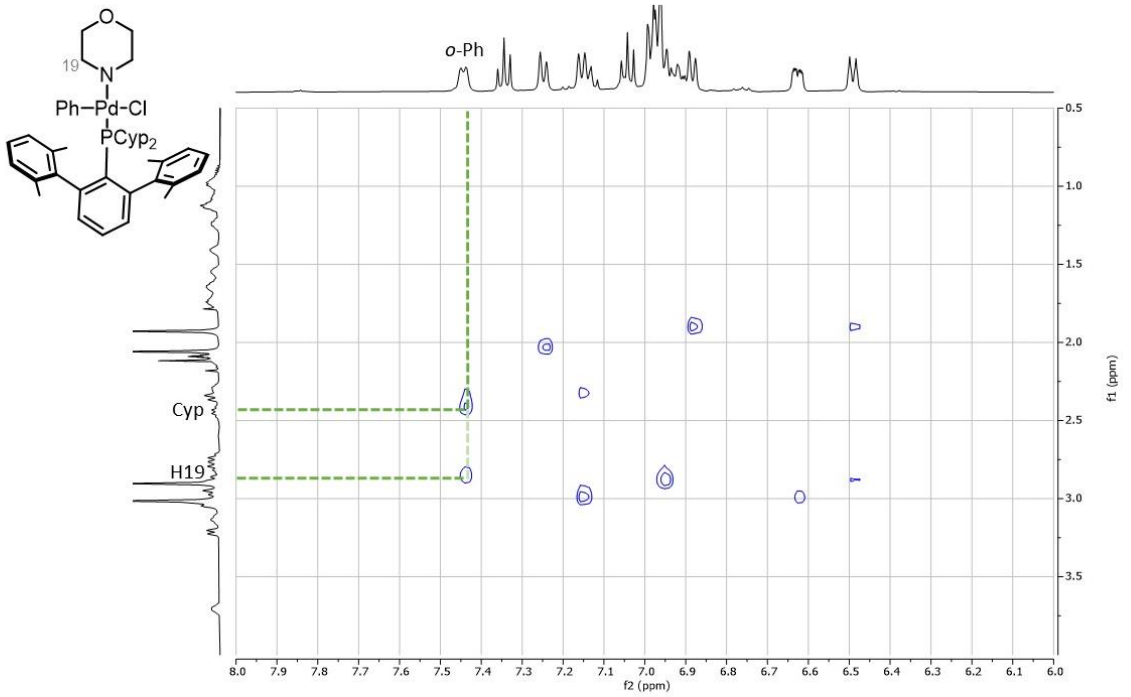

Figure S19. Selected region of a NOESY experiment of the complex **5M-Morph** in toluene-d_8 at 243 K.

2.6. NMR study of reductive elimination from [Pd(4-OMe-C_6H_4)(carbazolyl)(PCyp$_2$Ar^{Xyl2})], $\mathbf{8^{OMe}Cz}$

A solution of complex $\mathbf{8^{OMe}Cz}$ (5.0 mg, 0.006 mmol) in C_6D_6 (0.5 mL) was heated at 80 °C in an NMR tube prepared under nitrogen atmosphere. The reaction progress was monitored by ^{31}P NMR (Figure S20).

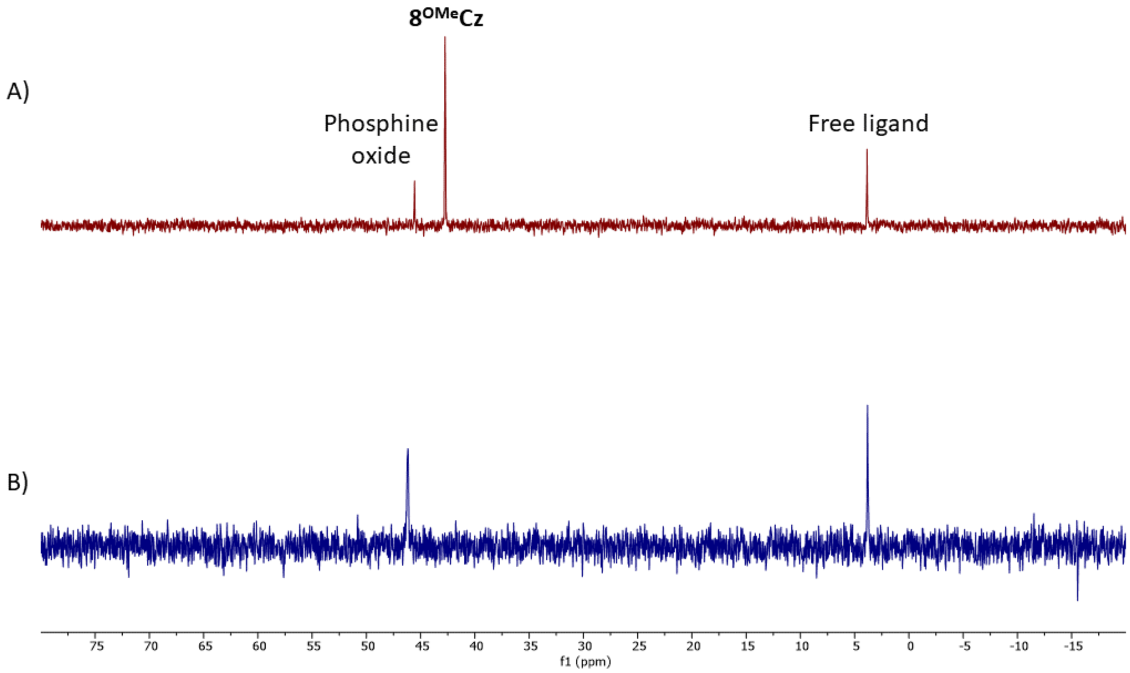

Figure S20. ^{31}P NMR spectrum of complex **8^OMe^Cz** after heating in C_6D_6 at 80 °C for (A) 30 min and (B) 2 h.

Figure S21 shows the ^{31}P NMR spectrum after 1.5 h of heating at 80 °C the solution of complex **8^OMe^Cz** (5.0 mg, 0.006 mmol) and dibenzylideneacetone (2.5 mg, 0.01 mmol) in C_6D_6 (0.5 mL).

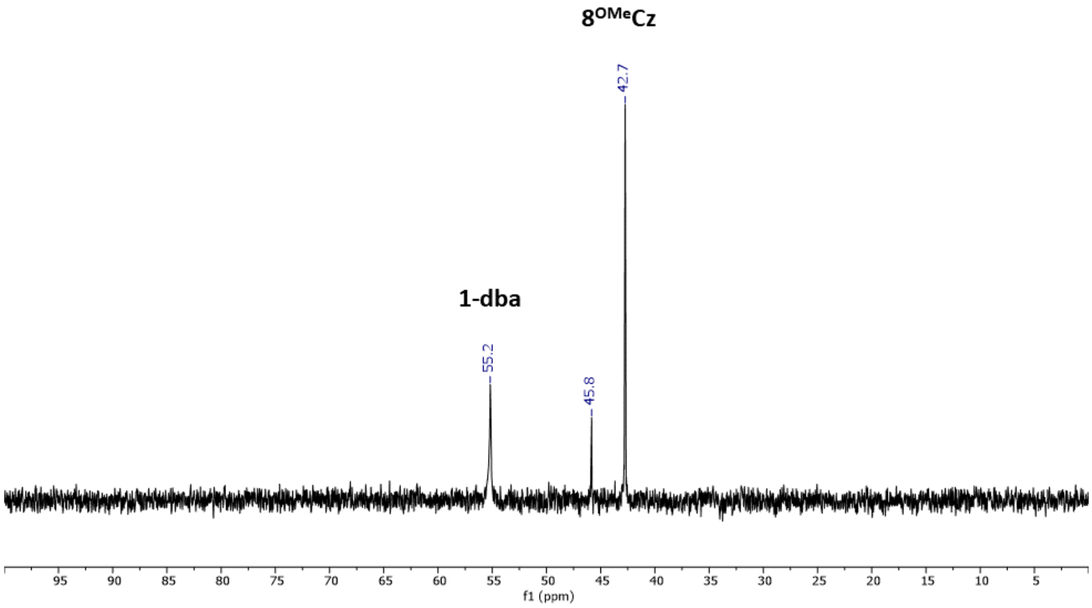

Figure S21. ^{31}P NMR spectrum of complex **8^OMe^Cz** after heating in C_6D_6 at 80 °C for 1.5 h in the presence of dba.

2.7. Catalyst resting state

Palladacycle **P1** (8.2 mg, 0.01 mmol), chlorobenzene (51 µL, 0.5 mmol), the amine (54.8 µL of aniline or 79.4 µL of hexylamine, 0.6 mmol), NaO*t*Bu (57.6 mg, 0.6 mmol) and C_6D_6 (0.5 mL) were added in turn into an NMR tube under nitrogen atmosphere. The reaction progress was monitored by ^{31}P NMR spectroscopy at room temperature (Figures S22-S23). The reaction crude was analyzed by GC and purified by flash chromatography.

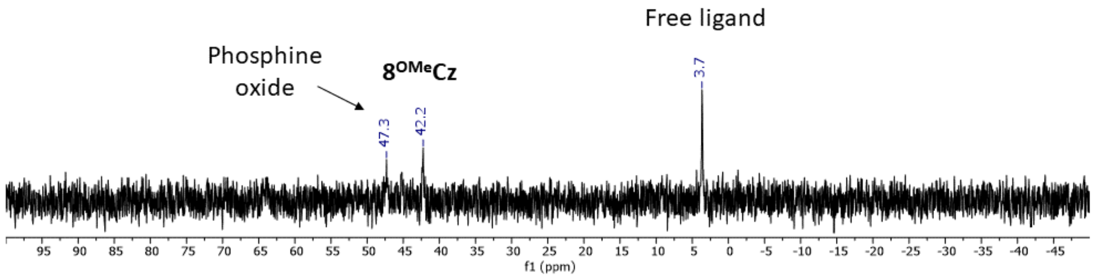

Figure S22. ^{31}P NMR spectrum of the reaction of aniline and chlorobenzene catalyzed by palladacycle **P1** after 15 min at room temperature.

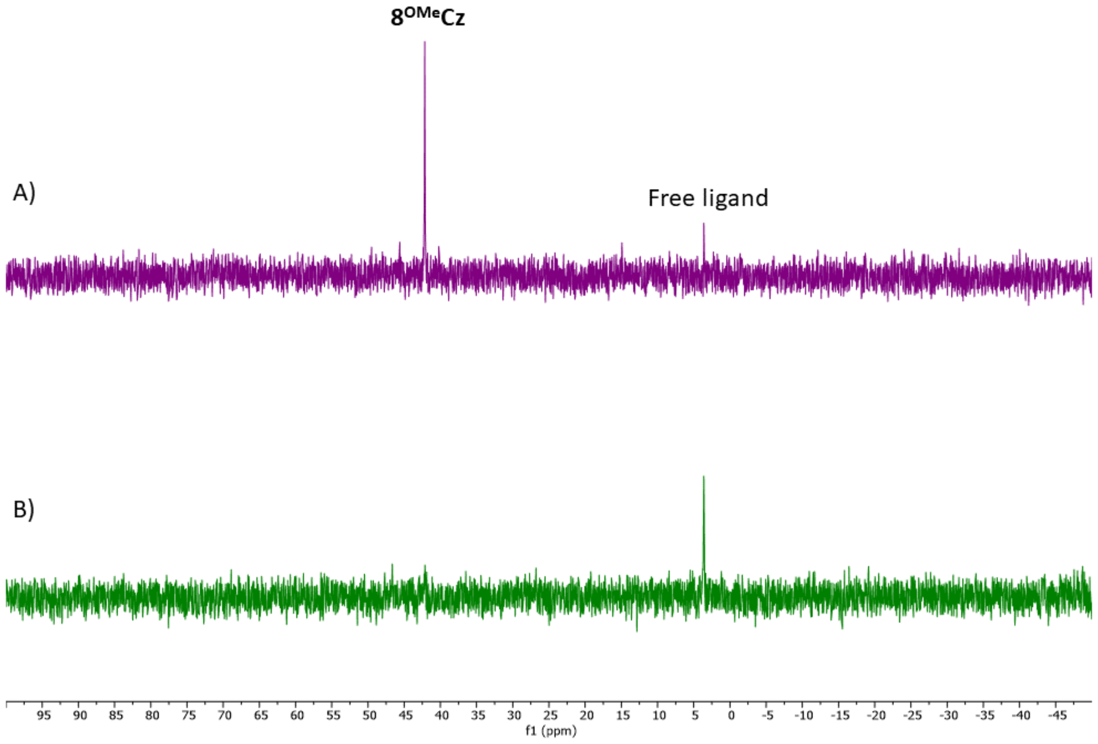

Figure S23. ^{31}P NMR spectrum of the reaction of hexylamine and chlorobenzene catalyzed by palladacycle **P1** after (A) 20 min and (B) 5 days at room temperature.

2.8. General catalytic procedure for testing the catalytic performance of isolated intermediates (Table 3).

The precatalyst (0.5 mol%) and the base NaO*t*Bu (115.3 mg, 1.2 mmol) were placed into a tube equipped with a J Young tap containing a magnetic bar. The amine (1.2 mmol), chlorobenzene (101.8 µL, 1 mmol) and the THF (1 mL) were added in turn, under a nitrogen atmosphere. The reaction mixture was stirred at room temperature for 24 h. The reaction mixture was diluted with ethyl acetate (10 mL) and filtered through a Celite plug. The crude product was purified by column chromatography

Yields for diphenylamine: 169.0 mg, 99% (**P1**); 154.1 mg, 91% (**3**); 152.2 mg, 90% (**5M-Hex**); 168.8 mg, 99% (**8Cz**); 159.8 mg, 95% (**P1'**).

Yields for *N*-hexylaniline: 82.5 mg, 47% (**P1**); 155.4 mg, 88% (**3**); 132.5 mg, 75% (**5M-Hex**); 84.0 mg, 47% (**8Cz**); 125.2 mg, 71% (**P1'**).

2.9. Catalytic performance of precatalyst P1 and on-cycle complex 3.

In order to prove the effect of carbazole in the reaction media, turnover numbers (TONs) were calculated by monitoring the reaction evolution of chlorobenzene with aniline or hexylamine using precatalyst **P1** and the on-cycle complex **3** as catalysts. As shown in Figure S24, the reaction with alkylamines is inhibited in the presence of carbazole (turnover frequency of 3.0 min^{-1} for **3** and 1.6 min^{-1} for **P1**). However, in the reaction of aniline precatalyst **P1** performs slightly better than complex **3** (turnover frequency of 25.2 min^{-1} for **3** and 33.7 min^{-1} for **P1**).

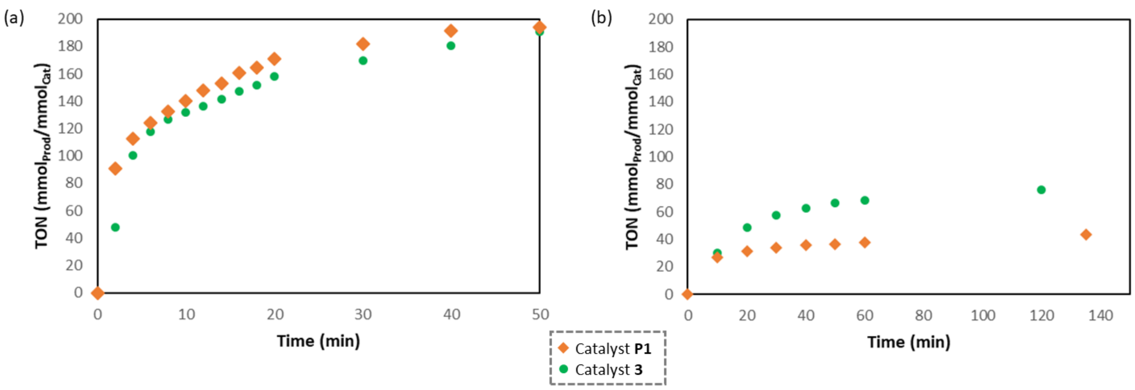

Figure S24. Comparison of the catalytic performance of precatalyst **P1** and complex **3** for the C-N coupling of chlorobenzene with (a) aniline and (b) hexylamine at room temperature.

3. Microkinetic modeling.

The complex reaction mechanism inferred from the calculations was interpreted by means of quantitative microkinetic models. Microkinetic models were constructed with the COPASI software[16] (version 4.35). Association, dissociation and ligand substitution reactions were assumed to have low Gibbs energy barriers ($\Delta G^{‡} \leq 3$ kcal·mol^{-1}) thus having no impact on the global kinetics of the reaction. Estimated transition states of reactions 8, 14, 18, 19, 22 and 23 as well as calculated **[TS8Cz-9Cz]** and **BaseCl** were fitted based on experimental data (*vide infra*). The initial concentrations used in the simulation were those reported in the experiments[12] (*i.e.* 0.80 M of aryl chloride, 0.98 M of amine, 0.98 M of NaO*t*Bu, 4.0 mM of catalyst). Following the experiments, simulations were carried out for a total time of 24 hours at T = 298 K or T = 355 K. The models were based on deterministic time course simulations with the LSODA algorithm.[17] The elementary steps of the mechanism underlying the microkinetic models are given in Figure S25, together with the $\Delta G^{‡}$ values derived from the DFT calculations and modifications by fitting experimental results.

reaction 1/ **P1** + Base ⇌ **P2** + BaseOMs

reaction 2/ **P2** ⇌ **P3**

reaction 3/ **P3** ⇌ **P4** + *t*BuOH

reaction 4/ **P4** ⇌ **P5**

reaction 5/ **P5** ⇌ **1** + Cz

reaction 6/ **1** + ClPh ⇌ **2**

reaction 7/ **2** ⇌ **3**

reaction 8/ **3** + Base ⇌ **3-OtBu** + BaseCl

reaction 9/ **3-OtBu** + NH_2Ph ⇌ **8A** + *t*BuOH

reaction 10/ **8A** ⇌ **9A**

reaction 11/ **9A** ⇌ **1** + $NHPh_2$

reaction 12/ **3** + NH_2Me ⇌ **5M**

reaction 13/ **5M** + Base ⇌ **5M-Base**

reaction 14/ **5M-Base** ⇌ **8M** + *t*BuOH + BaseCl

reaction 15/ **8M** ⇌ **9M**

reaction 16/ **9M** ⇌ **1** + NHMePh

reaction 17/ Cz + Base ⇌ **BaseCz** + *t*BuOH

reaction 18/ **BaseCz** + **3** ⇌ **8Cz** + BaseCl

reaction 19/ **8Cz** ⇌ **9Cz**

reaction 20/ **9Cz** ⇌ **1** + CzPh

reaction 21/ NH_2Ph + Base ⇌ **BaseNHPh** + *t*BuOH

reaction 22/ **BaseNHPh** + **8Cz** ⇌ **8A** + **BaseCz**

reaction 23/ **BaseNHPh** + **3** ⇌ **8A** + BaseCl

reaction 24/ NH_2Me + Base ⇌ **BaseNHMe** + *t*BuOH

reaction 25/ **BaseNHMe** + **8Cz** ⇌ **8M** + **10Cz**

reaction 26/ **BaseNHMe** + **3** ⇌ **8M** + BaseCl

ClPh = Chlorobenzene
CzPh = *N*-Phenylcarbazole
NHMePh = *N*-methylaniline

	ΔG‡ Forward (kcal·mol^{-1})	ΔG‡ Backwards (kcal·mol^{-1})
reaction 1	9.6	5.0
reaction 2	5.5	13.2
reaction 3	3.0	12.9
reaction 4	12.2	37.4
reaction 5	7.9	1.1
reaction 6	1.0	12.0
reaction 7	12.6	23.9
reaction 8[a]	19.7	19.0
reaction 9	6.2	11.5
reaction 10	13.6	30.9
reaction 11	12.1	3.0
reaction 12	0.1	1.3
reaction 13	1.0	5.3
reaction 14[a]	21.0	11.4
reaction 15	8.2	40.3
reaction 16	11.8	3.0
reaction 17	3.0	7.0
reaction 18[a]	14.7	27.2
reaction 19[b]	24.4	35.8
reaction 20	16.0	1.0
reaction 21	17.0	4.0
reaction 22[a]	6.2	11.3
reaction 23[a]	4.2	21.8
reaction 24	27.8	3.0
reaction 25	3.0	11.2
reaction 26	1.0	21.6

Figure S25. Reactions and corresponding Gibbs energies (kcal·mol^{-1}) used in the microkinetic model.[a] Transition state value fitted to match experimental yields. [b] Transition state value fitted using experimental data of the synthesis of **8Cz** from **P1** in the presence of chlorobenzene and NaO*t*Bu.

As noted before, some assigned or calculated transition state energies were estimated by fitting experimental results, due to the complexity of the system (in particular the NaO*t*Bu cluster model used for the base) and convergence problems during the calculations. Specifically, in the formation of diphenylamine, transition states of reactions 8, 18, 22 and 23 were fitted.

On the other hand, when the microkinetic analysis was applied to the formation of *N*-methylaniline, full conversion was obtained in only 7 min at room temperature, which does not match with experiments. We realized that the reductive elimination of complex **8Cz** proceed very fast at room temperature. Using experimental concentrations for the formation of carbazole from the precatalyst in the presence of the aryl halide and the base, we found out that the barrier of the reductive elimination of the *N*-arylated carbazole was underestimated by 2 kcal·mol^{-1}. The energy of the transition state of the deprotonation of the coordinated methylamine and the energy of the species **BaseCl** were fitted to match the yield obtained experimentally after 24h. This model also reproduces the equilibrium between **3** and **5M** in the reaction of **3** with an excess of the alkylamine at room temperature.

After the energy correction of **BaseCl**, the previously estimated transition states of reactions 8, 18, 22 and 23 were re-evaluated in reaction of aniline and chlorobenzene. With the new fitting, the reaction evolution curve matched the experimental results even better. Furthermore, the model reproduces selectivity experiments in the reaction of chlorobenzene with two nucleophiles (aniline and methylamine) at 80 ºC after 19 h, giving rise to 62% of *N*-methylaniline and 38% of diphenylamine (Scheme 9 and Figure S26).

When performing variations of the transition states we found that the rate limiting steps are reactions 8, 14, 18, 19, 22 and 23. Interestingly, these steps involve the base, either to replace the chloride anion or to deprotonate the amine.

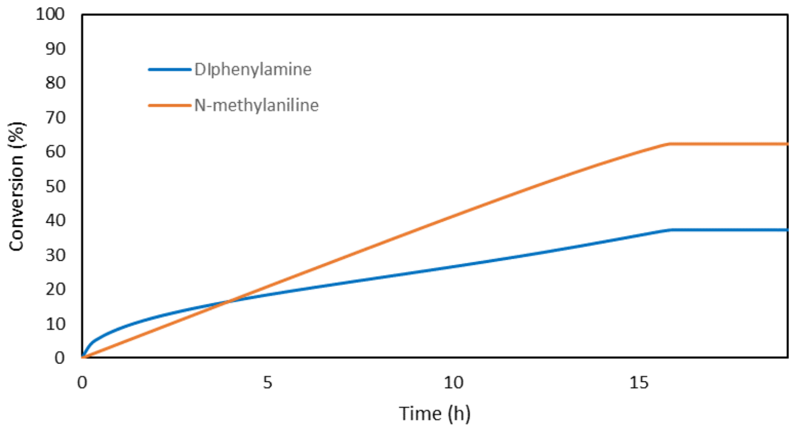

Figure S26. Modelled kinetic data of the reaction of chlorobenzene with aniline and methylamine catalyzed by **P1** at 80 °C for 19 h.

4. NMR spectra of compounds.

^{1}H NMR spectrum of [Pd(η^2-dba)(PCyp$_2$Ar^{Xyl2})], **1-dba**, (500 MHz, CD_2Cl_2, 233 K)

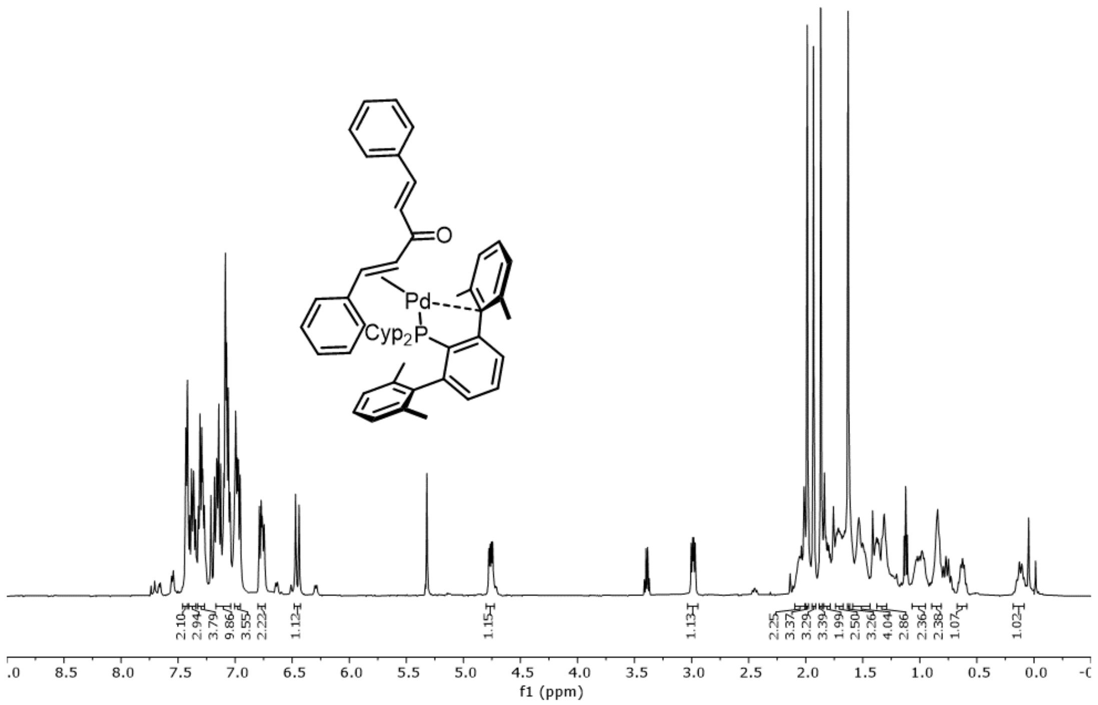

^{13}C{^{1}H} NMR spectrum of [Pd(η^2-dba)(PCyp$_2$Ar^{Xyl2})], **1-dba**, (125 MHz, CD_2Cl_2, 233 K)

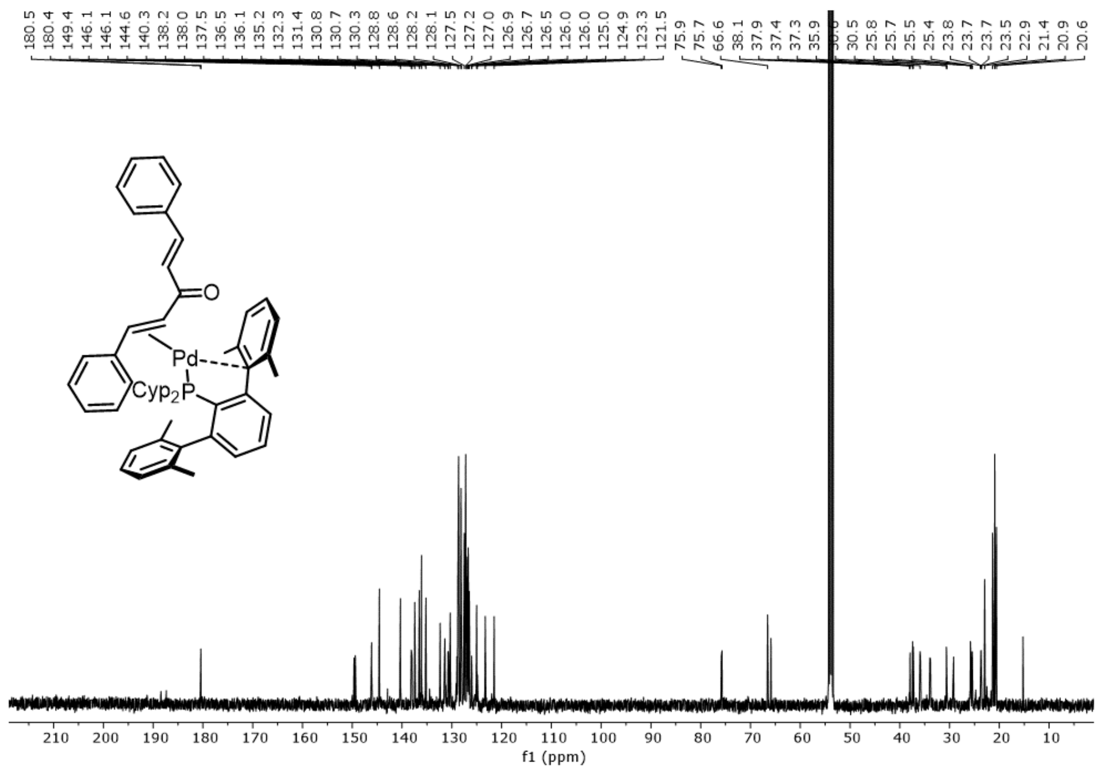

$^{31}P\{^{1}H\}$ NMR spectrum of [Pd(η^2-dba)($PCyp_2Ar^{Xyl2}$)], **1-dba**, (202 MHz, CD_2Cl_2, 233 K)

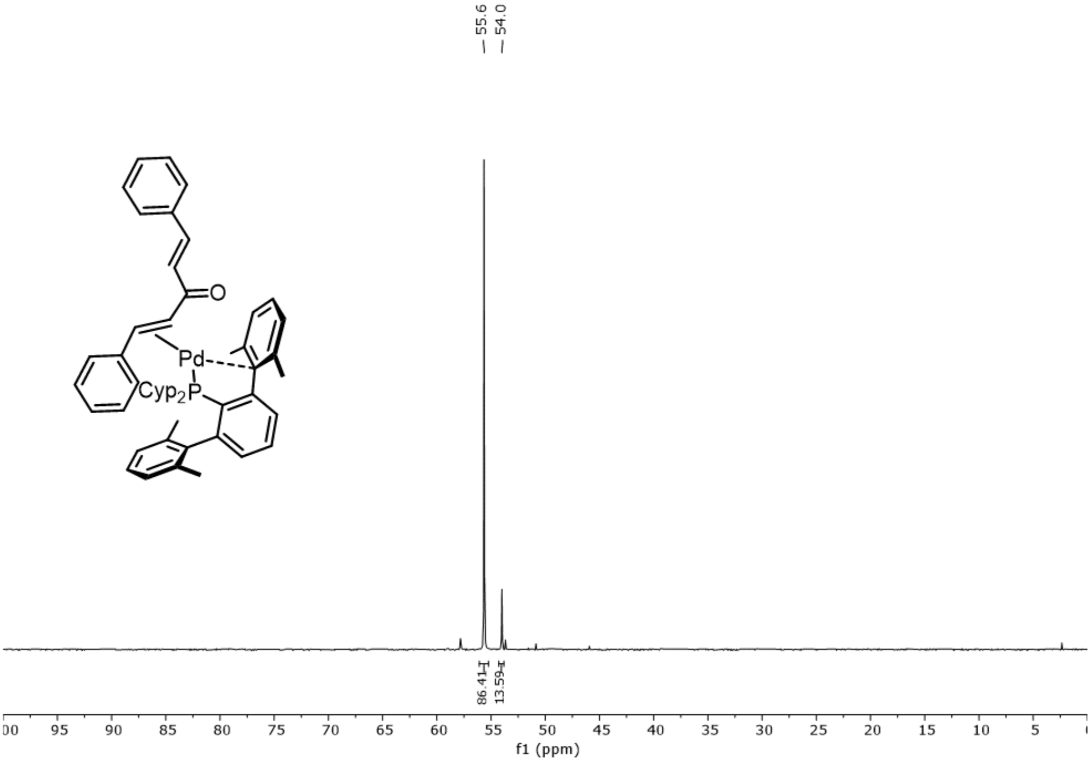

^{1}H NMR spectrum of [Pd(4-CHO-C_6H_4)(Cl)($PCyp_2Ar^{Xyl2}$)], **3^CHO** (300 MHz, $CDCl_3$, 298 K)

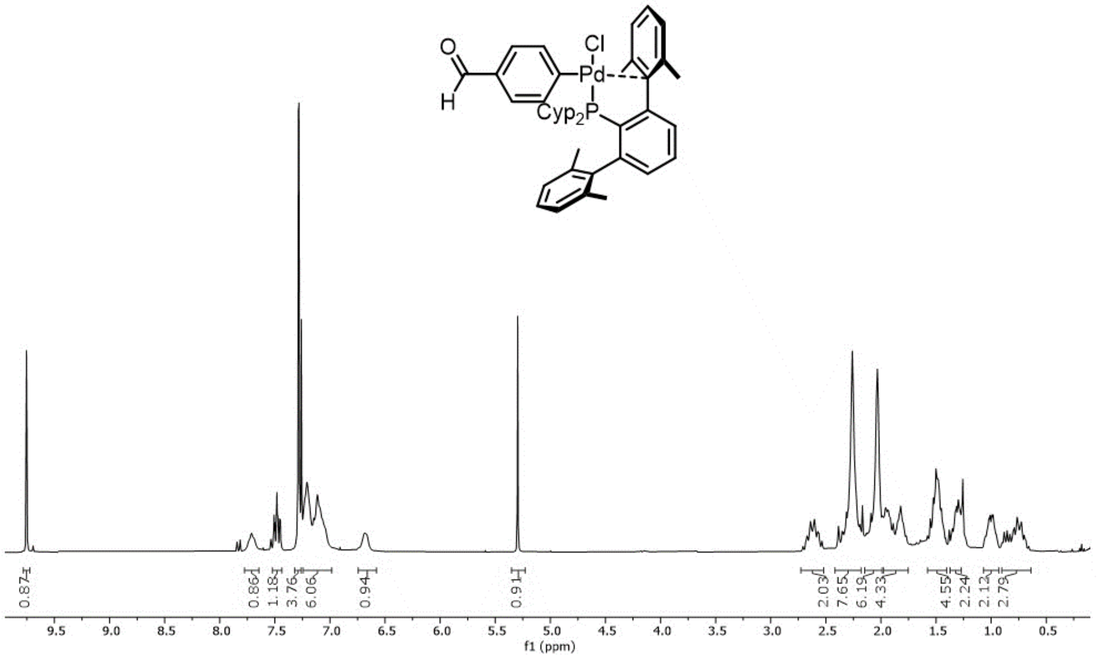

$^{13}C\{^{1}H\}$ NMR spectrum of [Pd(4-CHO-C_6H_4)(Cl)($PCyp_2Ar^{Xyl2}$)], **3^CHO** (75 MHz, $CDCl_3$, 298 K)

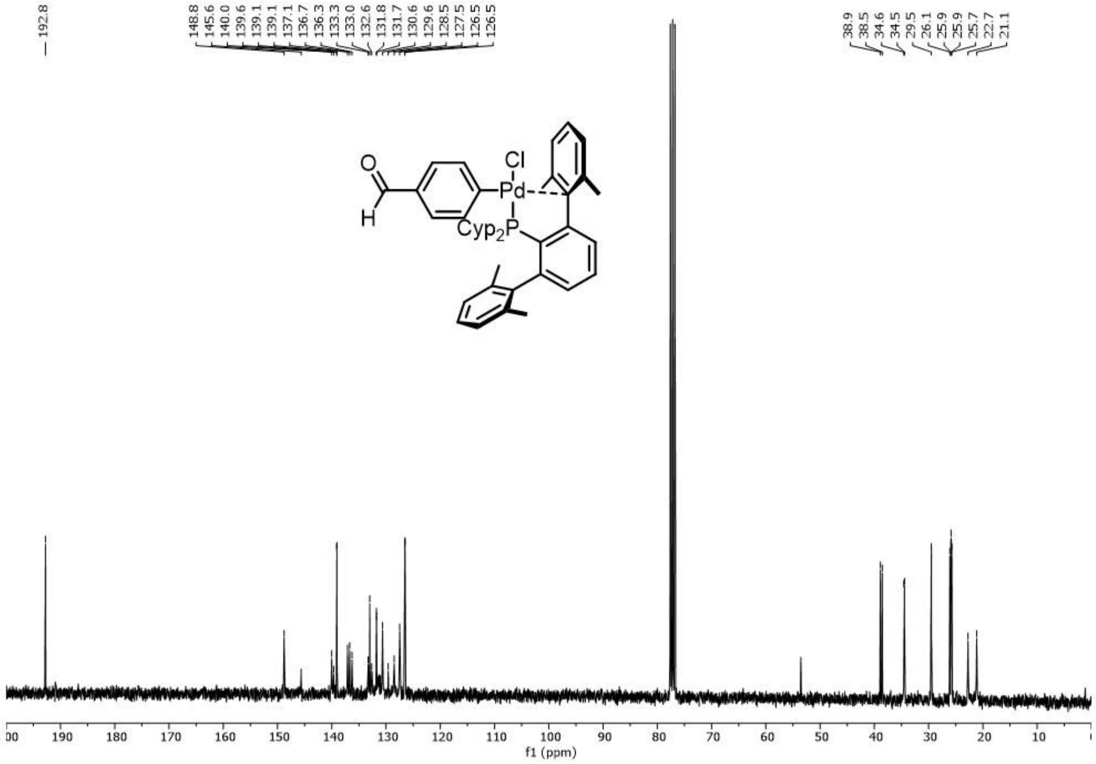

$^{31}P\{^1H\}$ NMR spectrum of [Pd(4-CHO-C_6H_4)(Cl)(PCyp$_2$Ar^{Xyl2})], **3^{CHO}** (121 MHz, $CDCl_3$, 298 K)

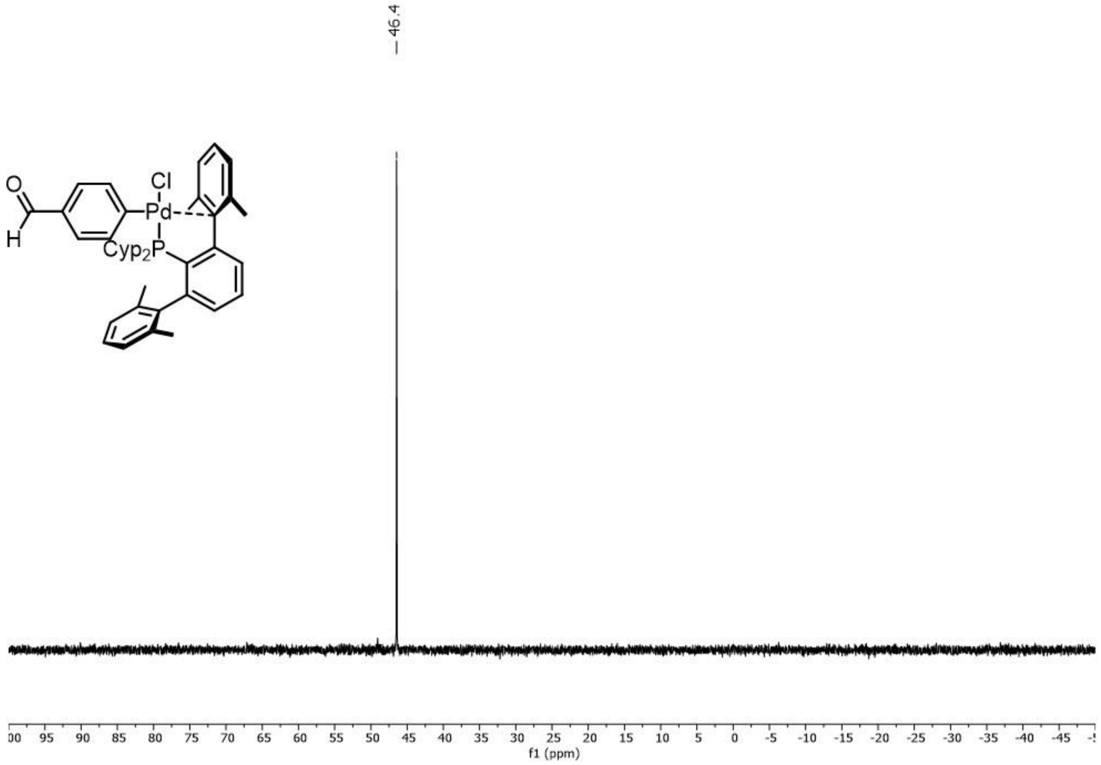

^1H NMR spectrum of [Pd(4-CF_3-C_6H_4)(Cl)($PCyp_2Ar^{Xyl2}$)], **3^{CF3}** (300 MHz, $CDCl_3$, 298 K)

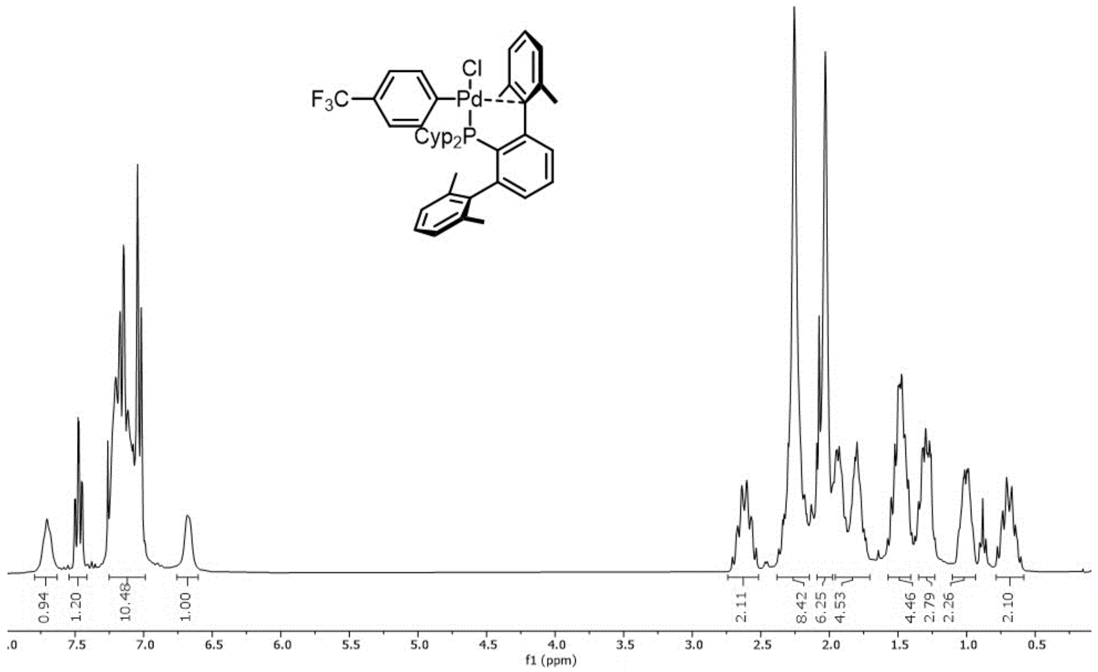

$^{13}C\{^1H\}$ NMR spectrum of [Pd(4-CF_3-C_6H_4)(Cl)($PCyp_2Ar^{Xyl2}$)], **3^{CF3}** (75 MHz, $CDCl_3$, 298 K)

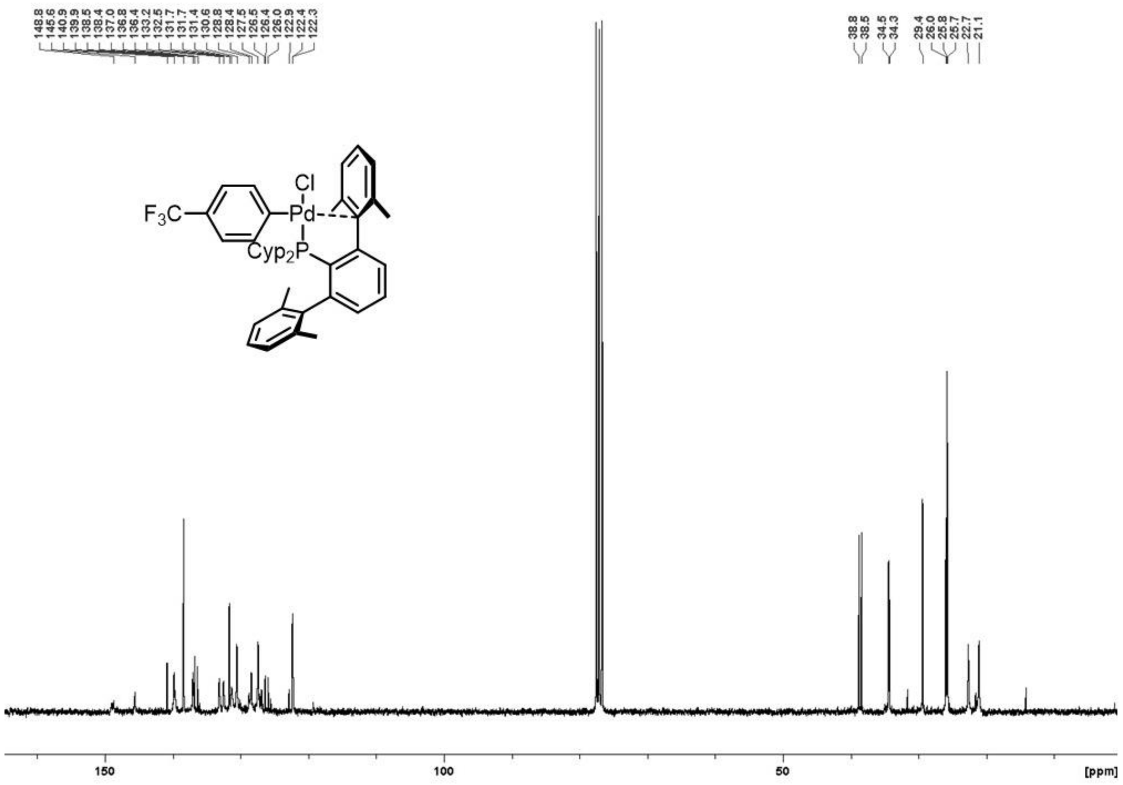

$^{31}P\{^{1}H\}$ NMR spectrum of [Pd(4-CF_3-C_6H_4)(Cl)($PCyp_2Ar^{Xyl2}$)], $\mathbf{3^{CF3}}$ (121 MHz, $CDCl_3$, 298 K)

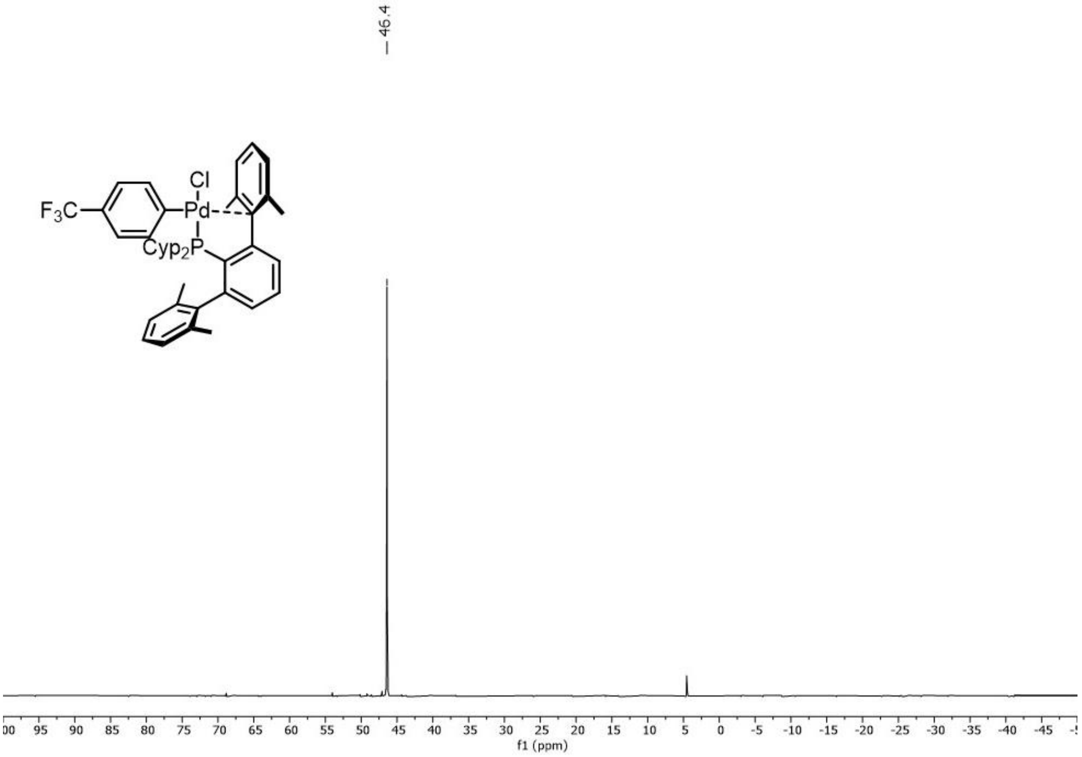

^{19}F NMR spectrum of [Pd(4-CF_3-C_6H_4)(Cl)($PCyp_2Ar^{Xyl2}$)], $\mathbf{3^{CF3}}$ (282 MHz, $CDCl_3$, 298 K)

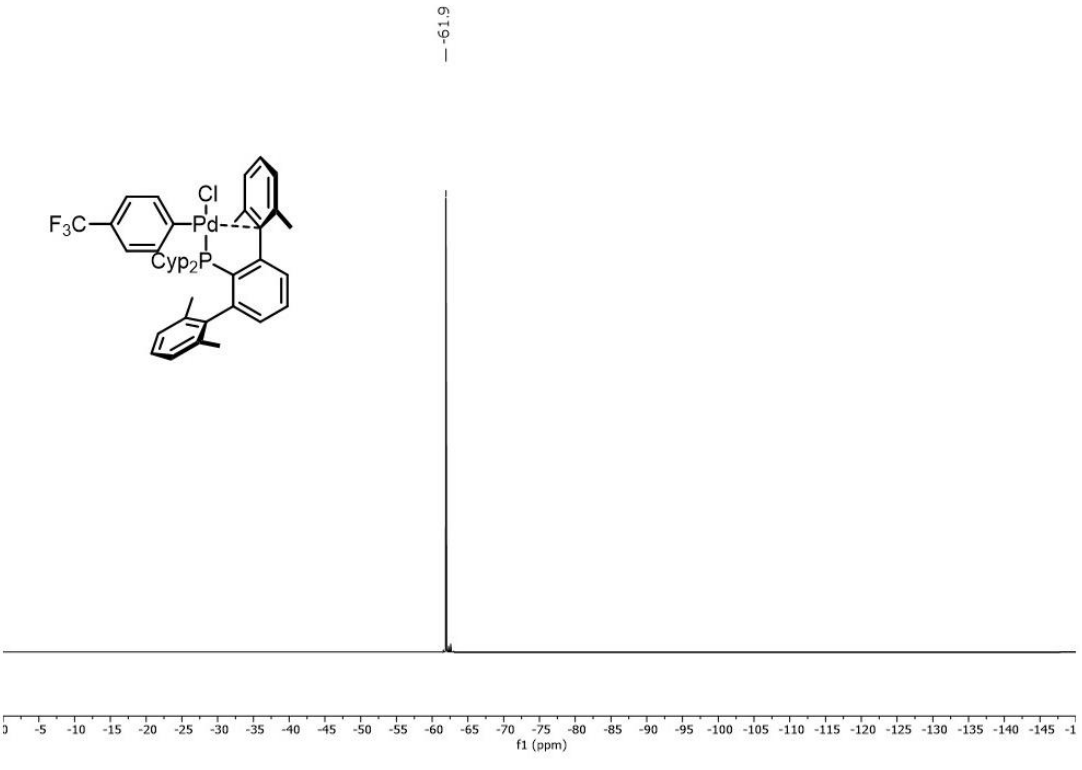

^{1}H NMR spectrum of [Pd(4-CN-C_6H_4)(Cl)(PCyp$_2$Ar^{Xyl2})], **3^{CN}** (400 MHz, $CDCl_3$, 263 K)

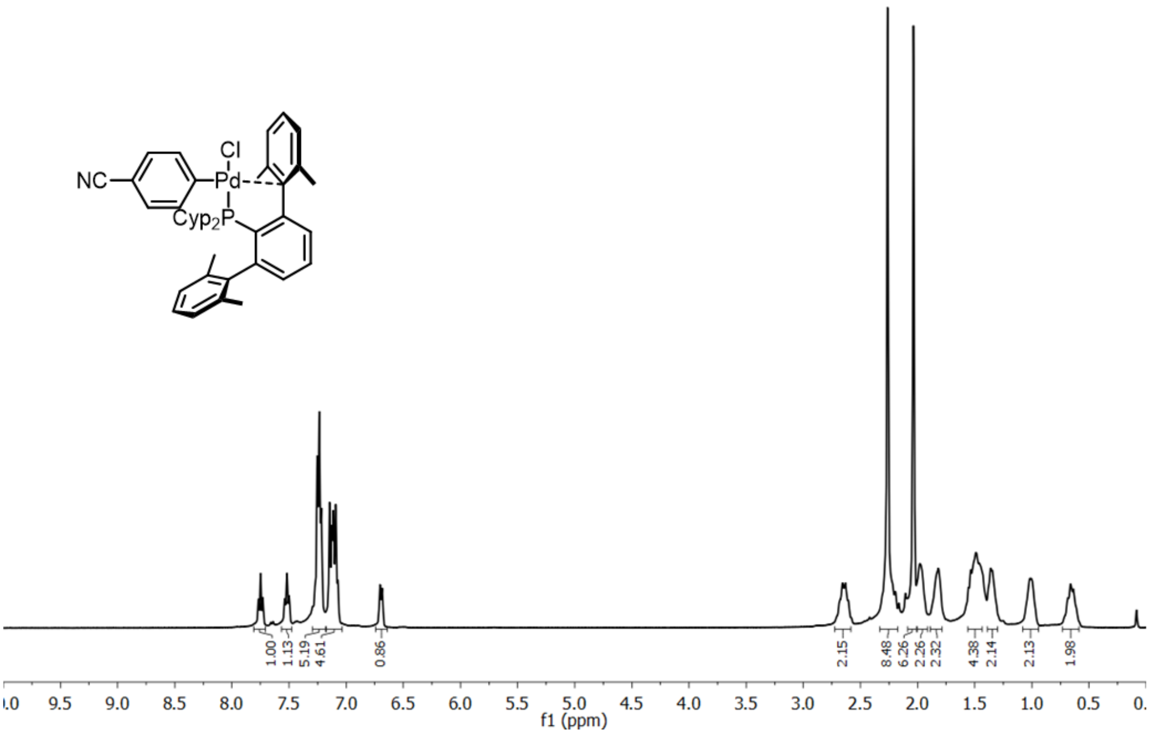

$^{13}C\{^{1}H\}$ NMR spectrum of [Pd(4-CN-C_6H_4)(Cl)(PCyp$_2$Ar^{Xyl2})], **3^{CN}** (100 MHz, $CDCl_3$, 263 K)

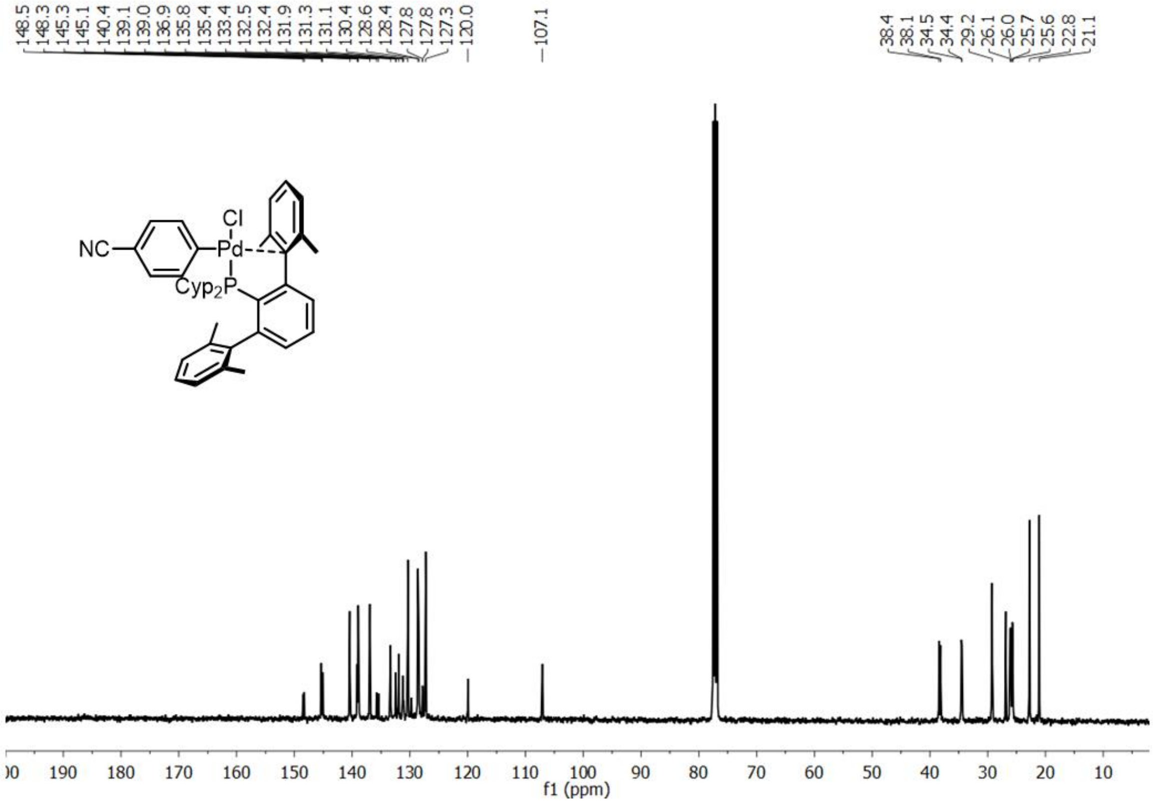

$^{31}P\{^{1}H\}$ NMR spectrum of [Pd(4-CN-C_6H_4)(Cl)($PCyp_2Ar^{Xyl2}$)], **3^CN** (121 MHz, $CDCl_3$, 298 K)

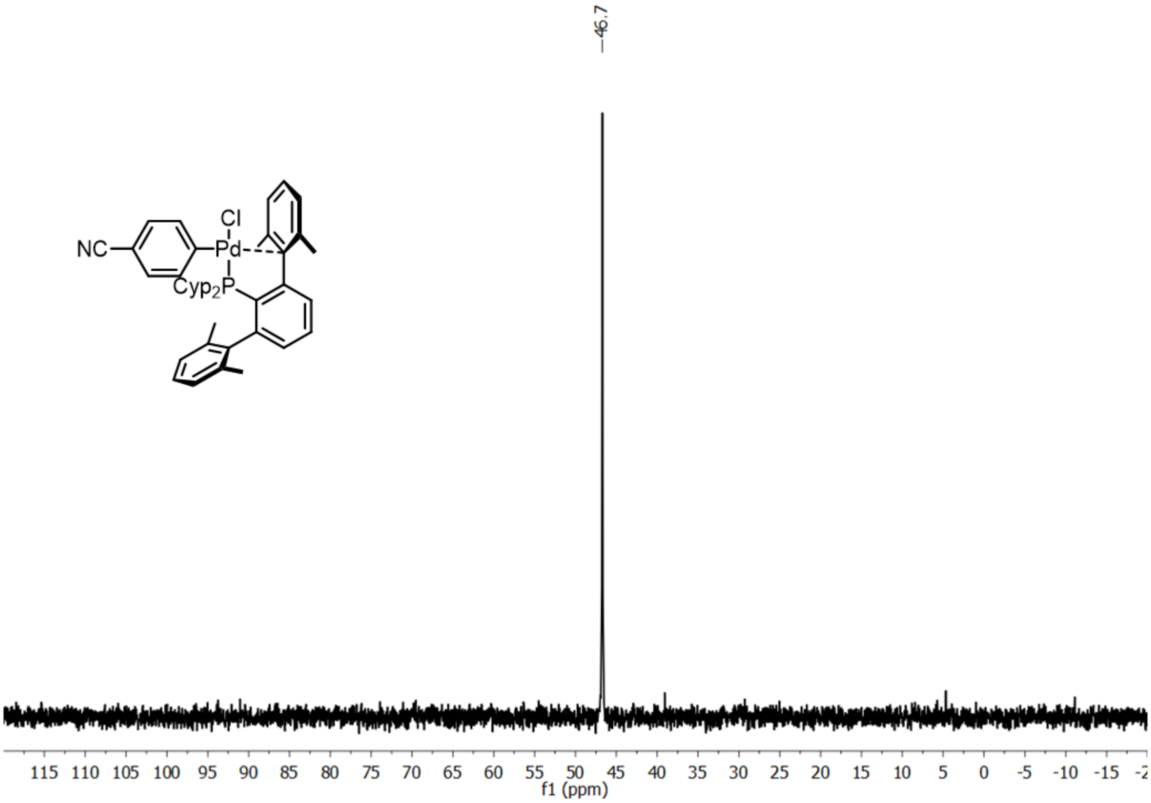

^{1}H NMR spectrum of [Pd(C_6H_5)(carbazolyl)(PCyp$_2$Ar^{Xyl2})], **8Cz** (300 MHz, $CDCl_3$, 289 K)

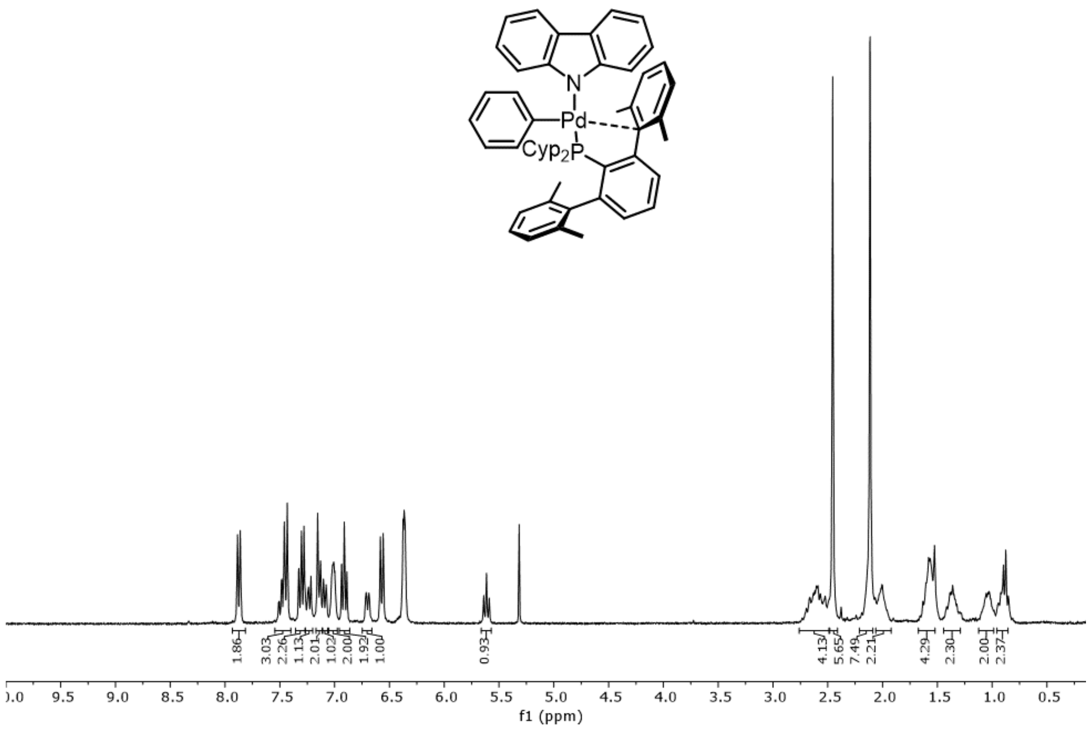

$^{13}C\{^{1}H\}$ NMR spectrum of [Pd(C_6H_5)(carbazolyl)(PCyp$_2$Ar^{Xyl2})], **8Cz** (100 MHz, $CDCl_3$, 298 K)

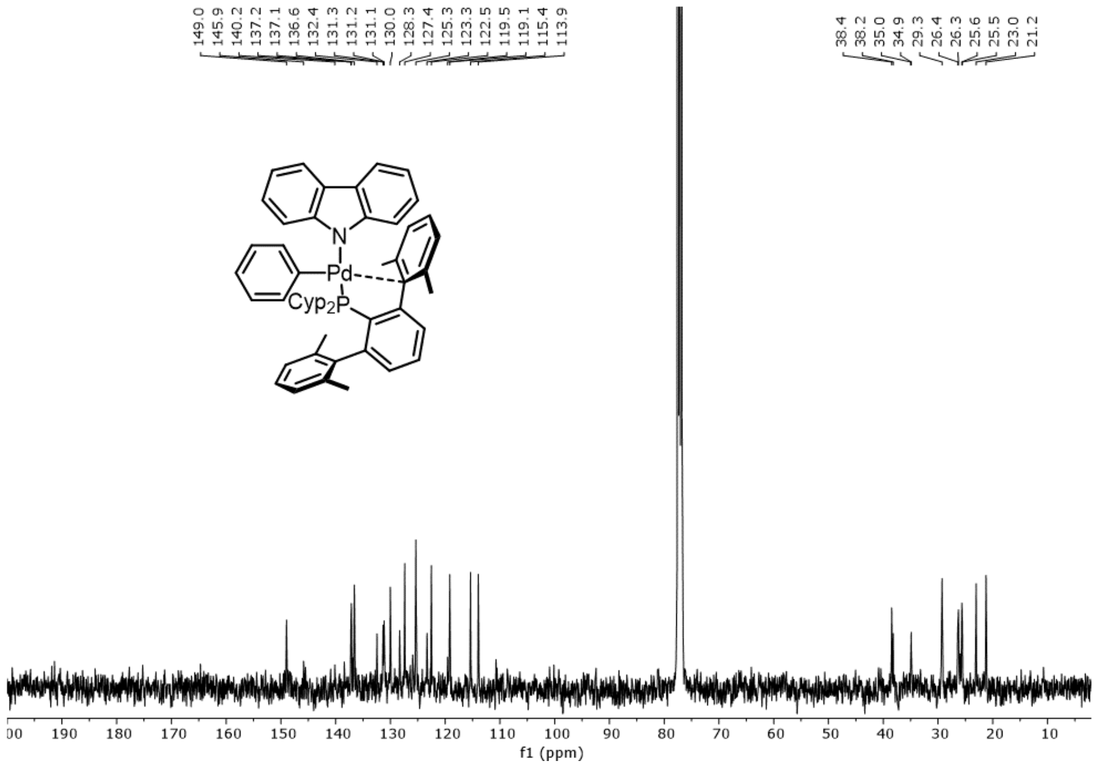

$^{31}P\{^{1}H\}$ NMR spectrum of [Pd(C_6H_5)(carbazolyl)(PCyp$_2$Ar^{Xyl2})], **8Cz** (121 MHz, $CDCl_3$, 298 K)

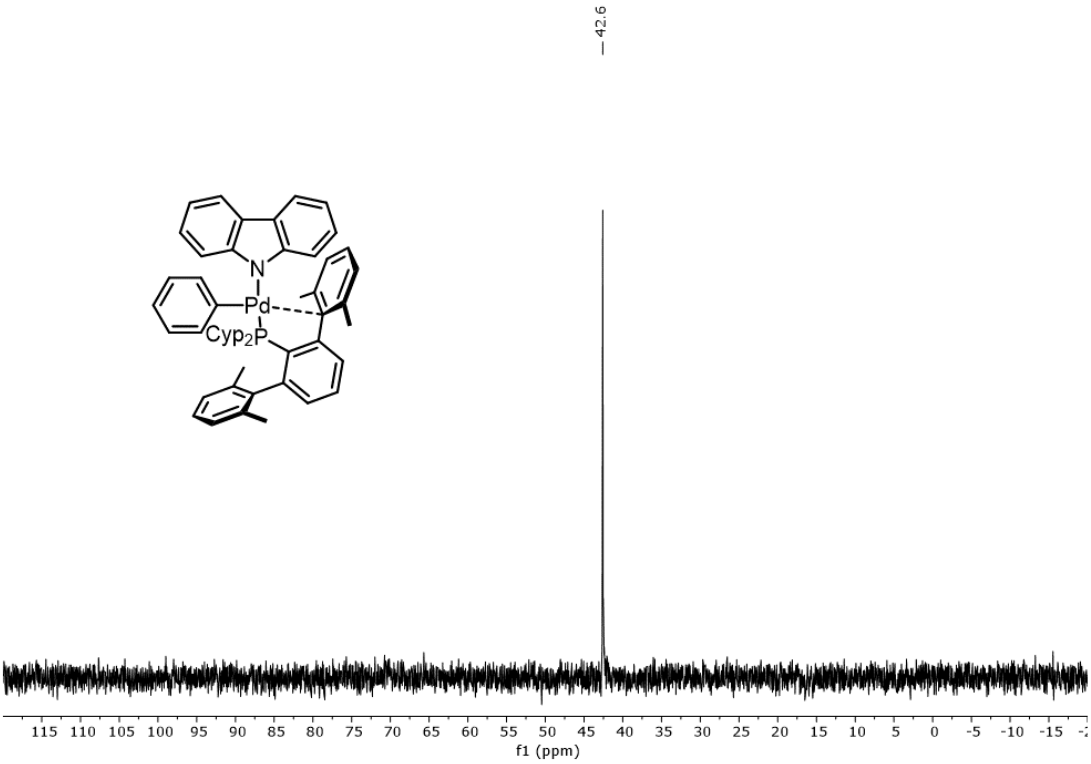
42.6
N
Pd
Cyp2P
115 110 105 100 95 90 85 80 75 70 65 60 55 50 45 40 35 30 25 20 15 10 5 0 -5 -10 -15
f1 (ppm)

^{1}H NMR spectrum of [Pd(4-OMe-C_6H_4)(carbazolyl)($PCyp_2Ar^{Xyl2}$)], **8^{OMe}Cz** (300 MHz, C_6D_6, 298 K)

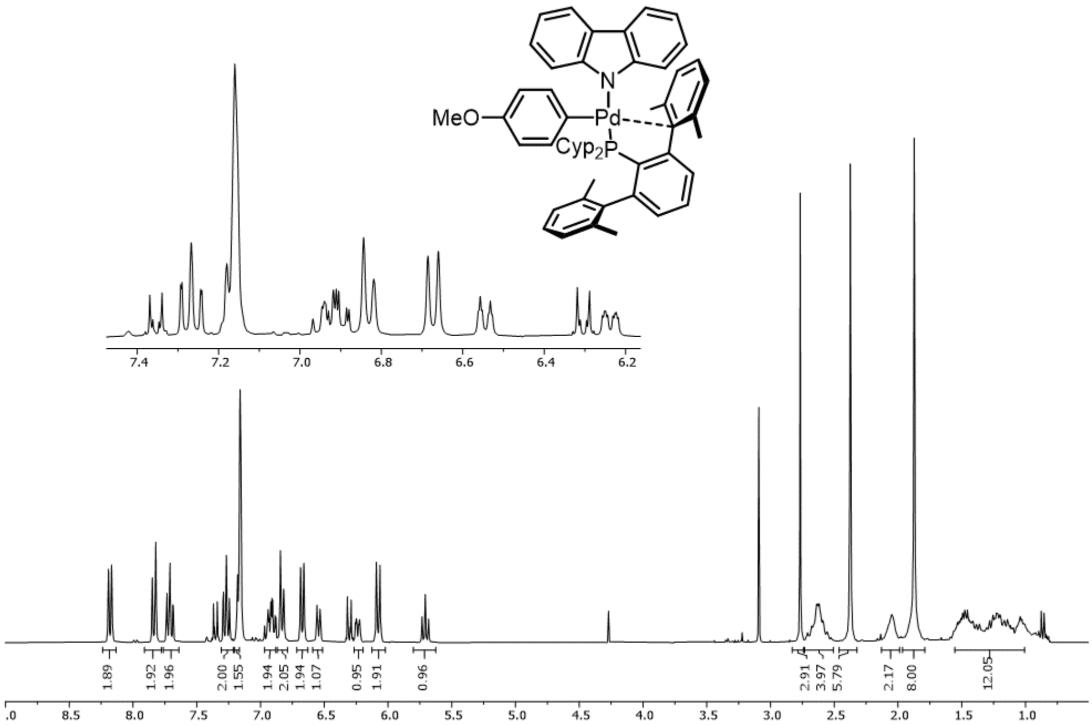

^{13}C{^{1}H} NMR spectrum of [Pd(4-OMe-C_6H_4)(carbazolyl)($PCyp_2Ar^{Xyl2}$)], **8^{OMe}Cz** (100 MHz, C_6D_6, 298 K)

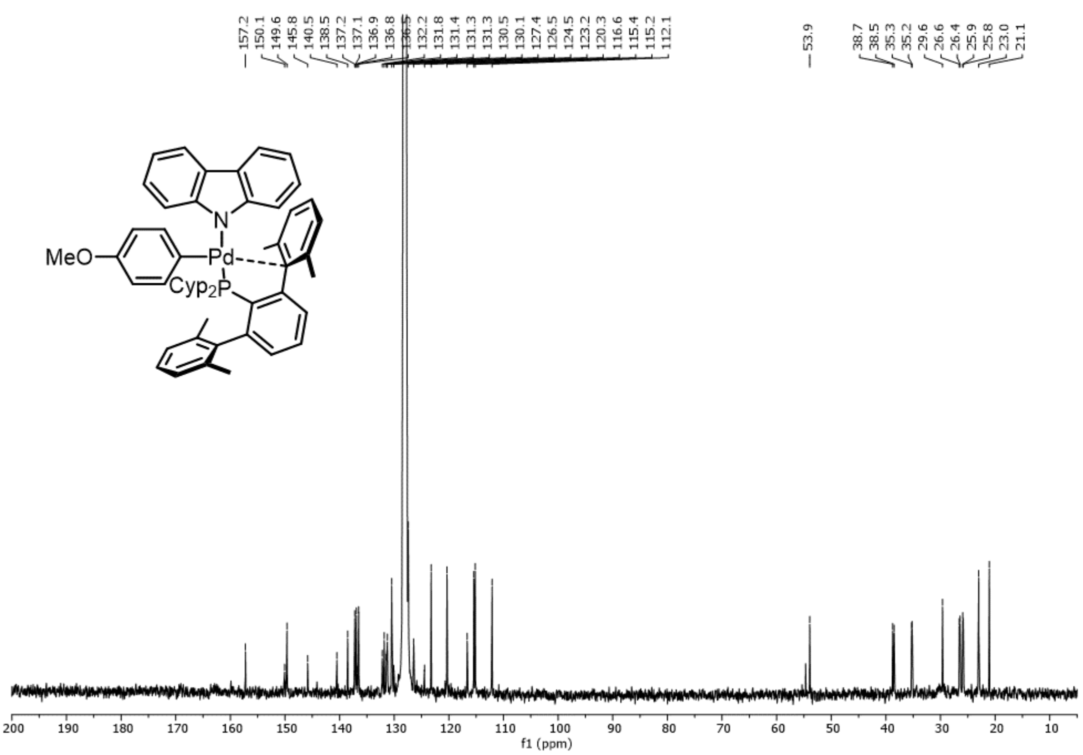

$^{31}P\{^{1}H\}$ NMR spectrum of [Pd(4-OMe-C_6H_4)(carbazolyl)($PCyp_2Ar^{Xyl2}$)], **8^{OMe}Cz** (121 MHz, C_6D_6, 298 K)

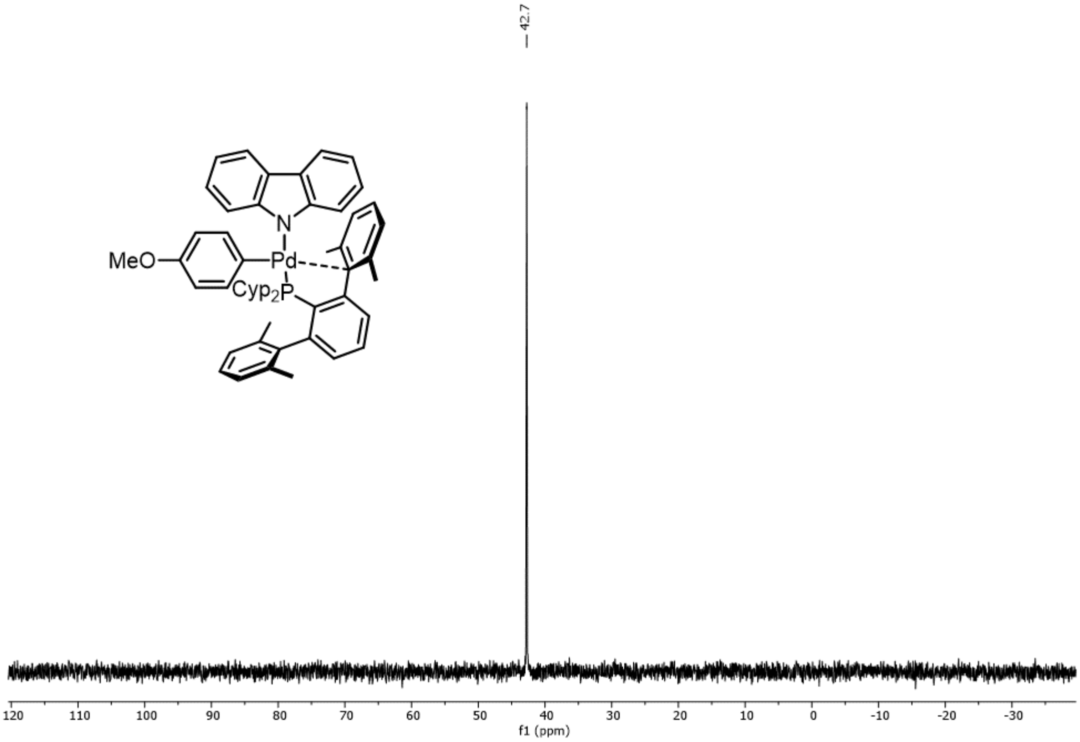

^{1}H NMR spectrum of $[Pd(C_6H_5)(morpholine)(Cl)(PCyp_2Ar^{Xyl2})]$, **5M-Morph** (500 MHz, toluene-d_8, 243 K)

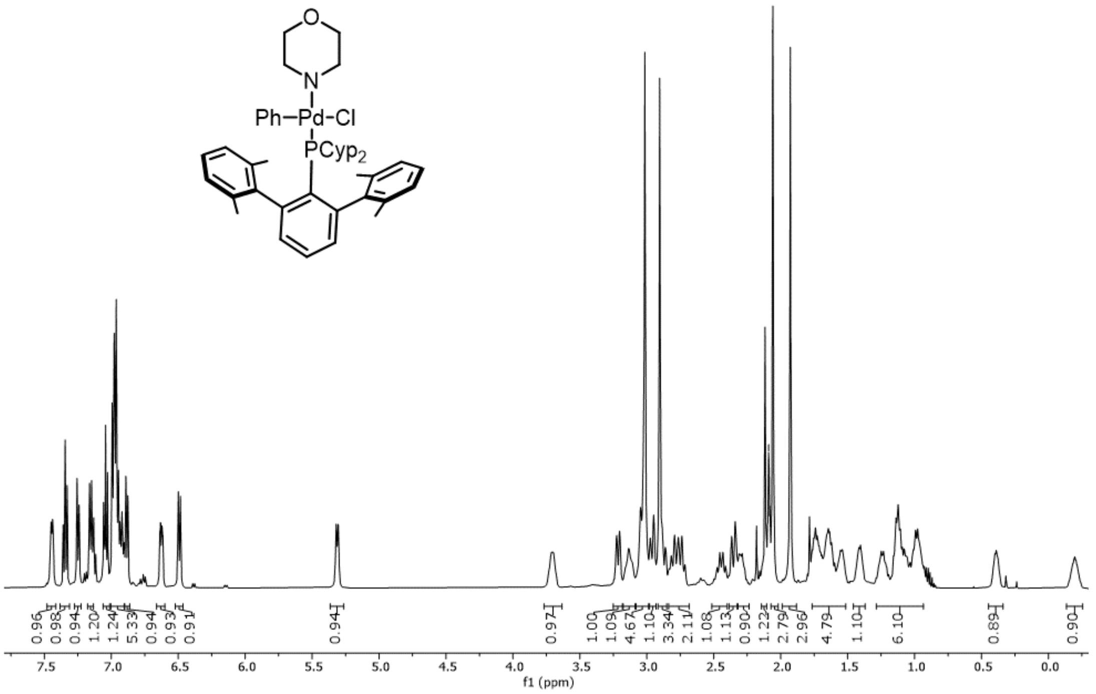

DEPTQ-$^{13}C\{^{1}H\}$ NMR spectrum of $[Pd(C_6H_5)(morpholine)(Cl)(PCyp_2Ar^{Xyl2})]$, **5M-Morph** (125 MHz, toluene-d_8, 243 K)

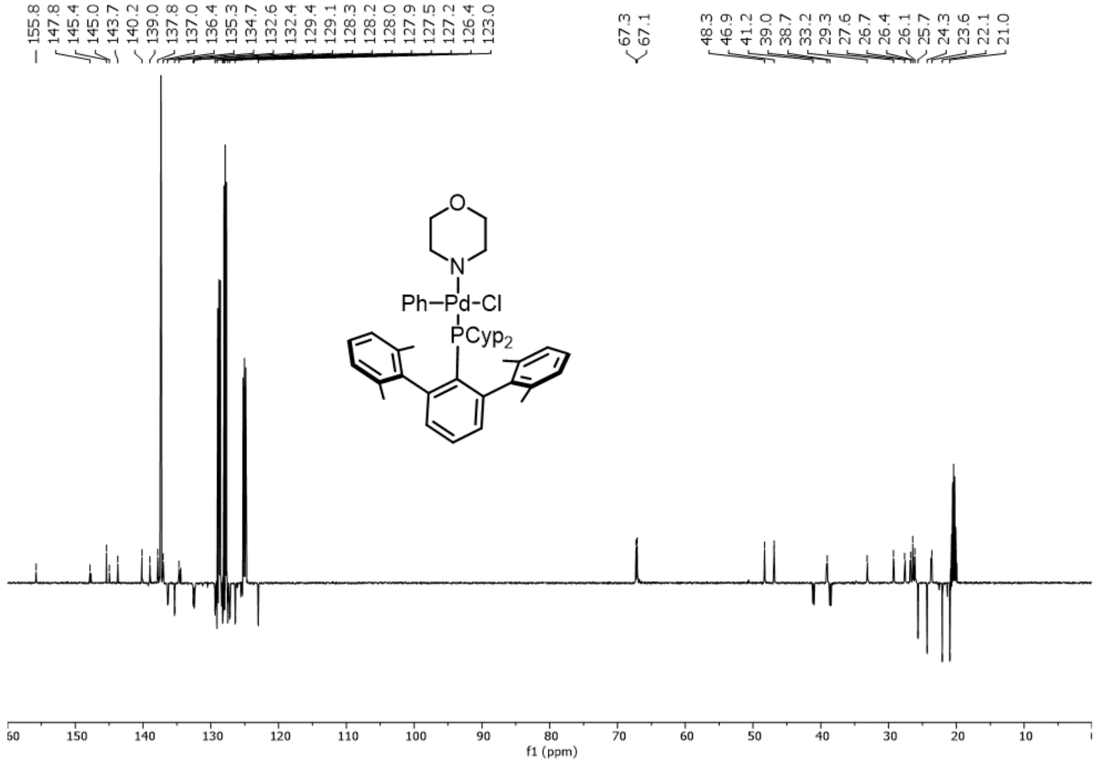

$^{31}P\{^1H\}$ NMR spectrum of $[Pd(C_6H_5)(morpholine)(Cl)(PCyp_2Ar^{Xyl2})]$, **5M-Morph** (202 MHz, toluene-d_8, 243 K)

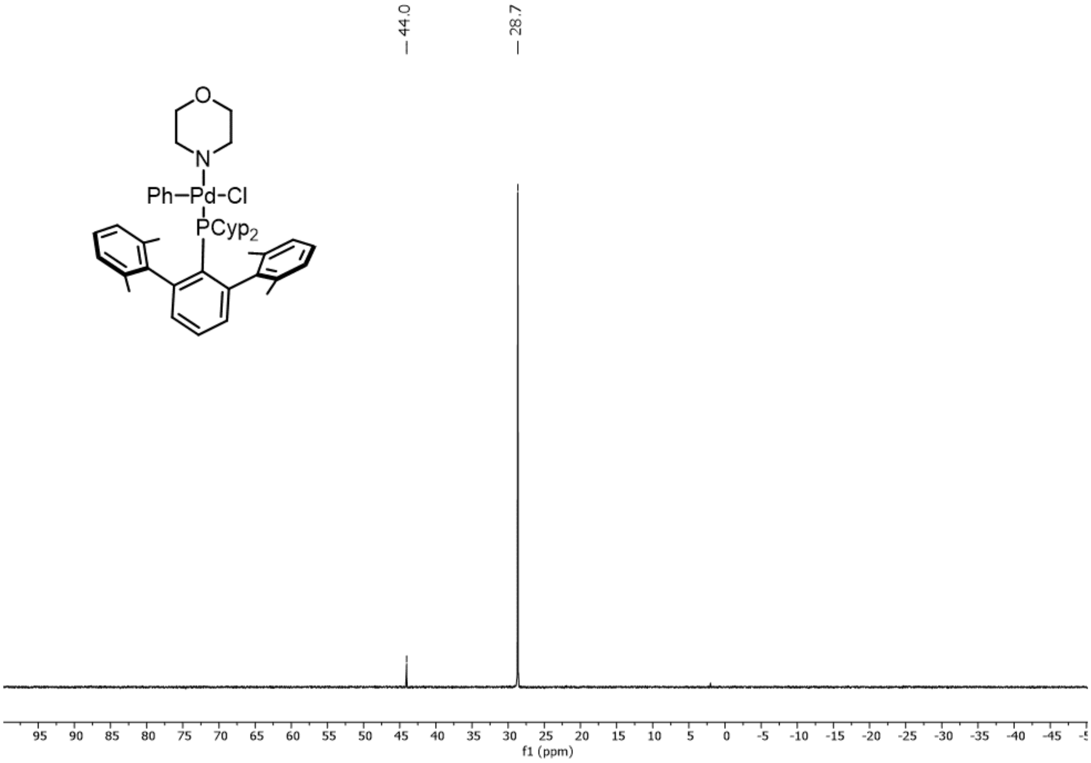

^{1}H NMR spectrum of $[Pd(C_6H_5)(Hex)(Cl)(PCyp_2Ar^{Xyl2})]$, **5M-Hex** (500 MHz, toluene-d_8, 243 K)

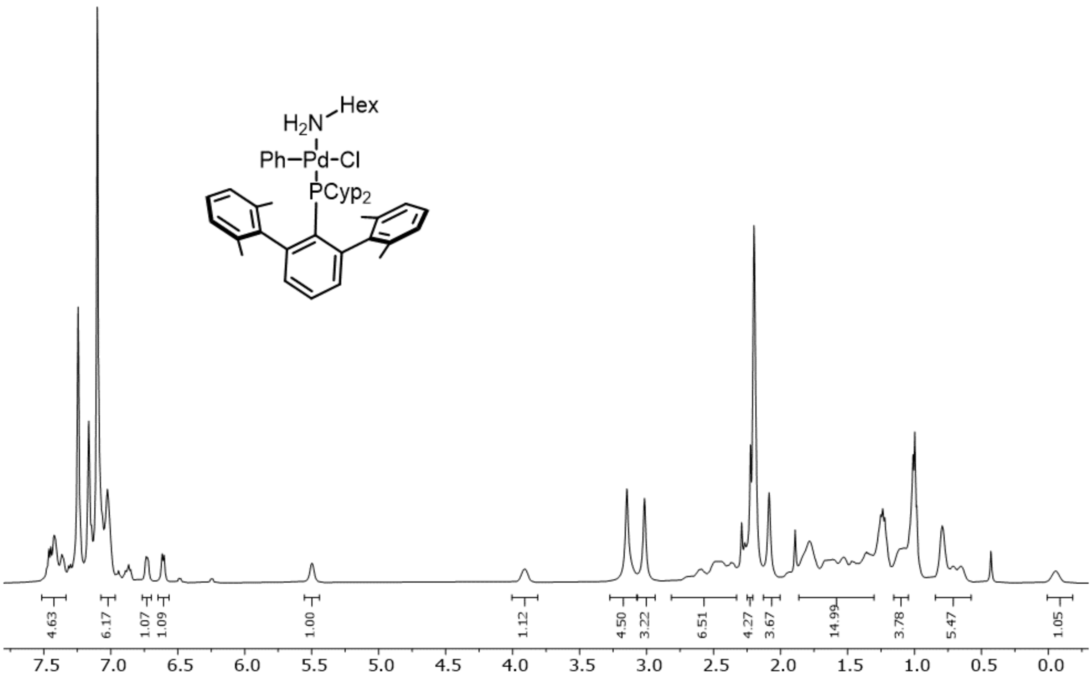

$^{31}P\{^{1}H\}$ NMR spectrum of $[Pd(C_6H_5)(Hex)(Cl)(PCyp_2Ar^{Xyl2})]$, **5M-Hex** (202 MHz, toluene-d_8, 243 K)

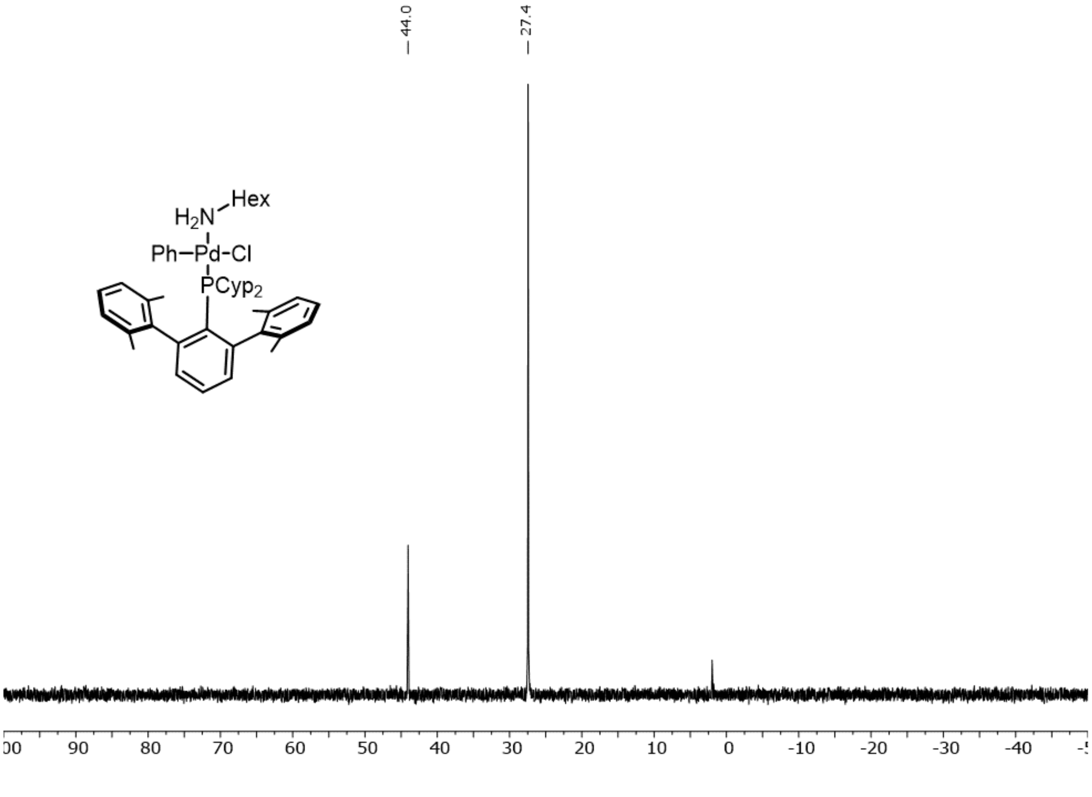

5. X-ray structural data of new complexes

Single crystals of suitable size for X-ray diffraction analysis of each compound were selected and coated with FOMBLIN oil, mounted on a loop and fixed in a cold nitrogen stream (T = 100 K) to the goniometer head. Data collection have been performed on two diffractometers:

- A Bruker Chi-Fixed QUEST diffractometer equipped with a Photon II CMOS detector, using MoKα1 (λ=0.71073 Å, microfocus sealed x-ray tube) and a Oxford Cryosystems low-temperature device (Cryostream 800), (used with **1-dba**).

- A Bruker-AXSX8Kappa diffractometer with an APEX-II CCD area detector, using a graphite monochromator Ag Kα1 (λ=0.56086 Å) and a Bruker Cryo-Flex low-temperature device (used with **3^{CN}** and **8^{OMe}Cz**), and

Reflections were merged and corrected for Lorenz and polarization effects using SAINT and absorption corrections were performed using SADABS[18] of APEX II software.[19] Using Olex2,[20] the structures were solved with SHELXS and were refined against F^2 on all data by full-matrix least squares with SHELXL.[21] All non-hydrogen atoms were refined anisotropically. Hydrogen atoms were included in the model at geometrically calculated positions and refined using a riding model. The isotropic displacement parameters of all hydrogen atoms were fixed to 1.2 times the U value of the atoms to which they are linked (1.5 times for methyl groups).

A summary of the fundamental crystal and refinement data are given in the Table S1. Atomic coordinates, anisotropic displacement parameters and bond lengths and angles can be found in the cif files which have been deposited in the Cambridge Crystallographic Data Centre with no. 2221856 (**1-dba**), 2224943 (**3^{CN}**) and 2224944 (**8^{OMe}Cz**). These data can be obtained free for charge from The Cambridge Crystallographic Data Centre via www.ccdc.cam.ac.uk/data_request/cif.

Table S1. *Crystal data and structure refinement for **1-dba**, **3^{CN}** and **8^{OMe}Cz***

Identification code	**1-dba**	**3^{CN}**	**8^{OMe}Cz**
Empirical formula	$C_{51}H_{58}O_{1.50}PPd$	$C_{39}H_{43}ClNPPd$	$C_{108}H_{112}IN_2O_2P_2Pd_2$
Formula weight	832.34	698.56	1871.63
Temperature	100.0 K	100.0 K	100.0 K
Wavelength	0.71073 Å	0.56086 Å	0.56086 Å

Crystal system	Triclinic	Orthorhombic	Monoclinic
Space group	P-1	$P2_12_12_1$	$P2_1/c$
Unit cell dimensions	a = 12.0606(9) Å<br>b = 12.8472(10) Å<br>c = 15.8516(12) Å<br>α = 67.006(2)°<br>β = 75.214(2)°<br>γ = 71.299(2)°	a = 9.4797(3) Å<br>b = 14.5710(5) Å<br>c = 24.2860(9)Å<br>α = 90°<br>β = 90°<br>γ = 90°	a = 15.6964(14) Å<br>b = 12.0459(10) Å<br>c = 24.0221(18) Å<br>α = 90°<br>β = 104.496(3)°<br>γ = 90°
Volume	2117.7(3) $Å^3$	3354.6(2) $Å^3$	4397.4(6) $Å^3$
Z	2	4	2
Density (calculated)	1.305 Mg/m^3	1.383 Mg/m^3	1.414 Mg/m^3
Absorption coefficient	0.514 mm^{-1}	0.374 mm^{-1}	0.450 mm^{-1}
F(000)	874	1448	1930
Crystal size	0.2 x 0.13 x 0.045 mm^3	0.21 × 0.16 × 0.13 mm^3	0.22 × 0.18 × 0.15 mm^3
Theta range for data collection	2.118 to 25.413°	3.446 to 47.372°	2.764 to 44.218°
Index ranges	-14<=h<=14,<br>-15<=k<=15,<br>-19<=l<=19	-13<=h<=12,<br>-20<=k<=20,<br>-33<=l<=34	-21<=h<=21,<br>-16<=k<=16,<br>-32<=l<=27
Reflections collected	50188	56063	47628
Independent reflections	7784<br>[R(int) = 0.0647]	10324<br>[R(int) = 0.0487]	11070<br>[R(int) = 0.0423]
Completeness to theta = 22.00°	99.7 %	99.9 %	99.9 %
Absorption correction	Semi-empirical from equivalents	Semi-empirical from equivalents	Semi-empirical from equivalents
Max. and min. transmission	0.0916 and 0.0636	0.66 and 0.75	0.59 and 0.75
Refinement method	Full-matrix least-squares on F^2	Full-matrix least-squares on F^2	Full-matrix least-squares on F^2
Data / restraints / parameters	7784 / 363 / 577	10324 / 0 / 393	11070 / 6 / 563
Goodness-of-fit on F^2	1.131	1.030	1.064
Final R indices [I>2sigma(I)]	R1 = 0.0852,<br>wR2 = 0.2513	R1 = 0.0294,<br>wR2 = 0.0596	R1 = 0.0444,<br>wR2 = 0.1044
R indices (all data)	R1 = 0.0984,<br>wR2 = 0.2606	R1 = 0.0356,<br>wR2 = 0.0622	R1 = 0.0552,<br>wR2 = 0.1109
Largest diff. peak and hole	2.426 and -1.364 e.$Å^{-3}$	0.35 and -0.53 e.$Å^{-3}$	2.28 and -1.26 e.$Å^{-3}$

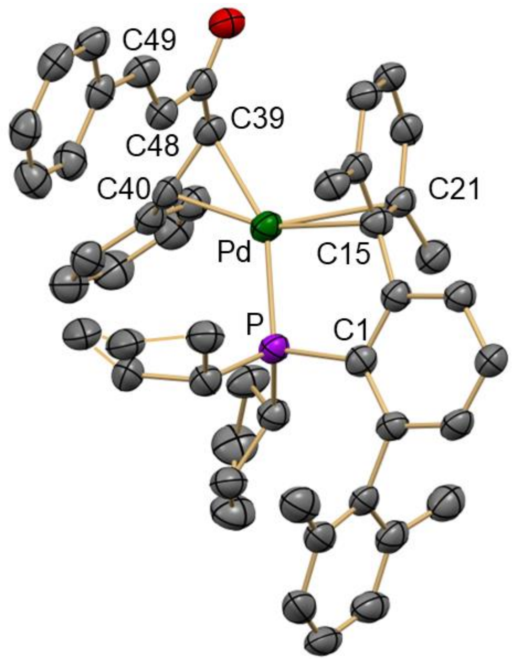

Figure S26. Molecular structure of complex **1-dba**. Hydrogen atoms are omitted for clarity and thermal ellipsoids are set at 50% level probability. Selected distances [Å] and angles [°]:Pd-P 2.313(2), Pd-C39 2.162(8), Pd-C40 2.114(7), Pd-C15 2.329(8), Pd-C21 2.533(8), C39-C40 1.423(11), C48-C49 1.325(12); dbaCentroid-Pd-P 134.65, AreneCentroid-Pd-P 90.84, P-C1-C2 113.5(5), P-C1-C6 127.2(6).

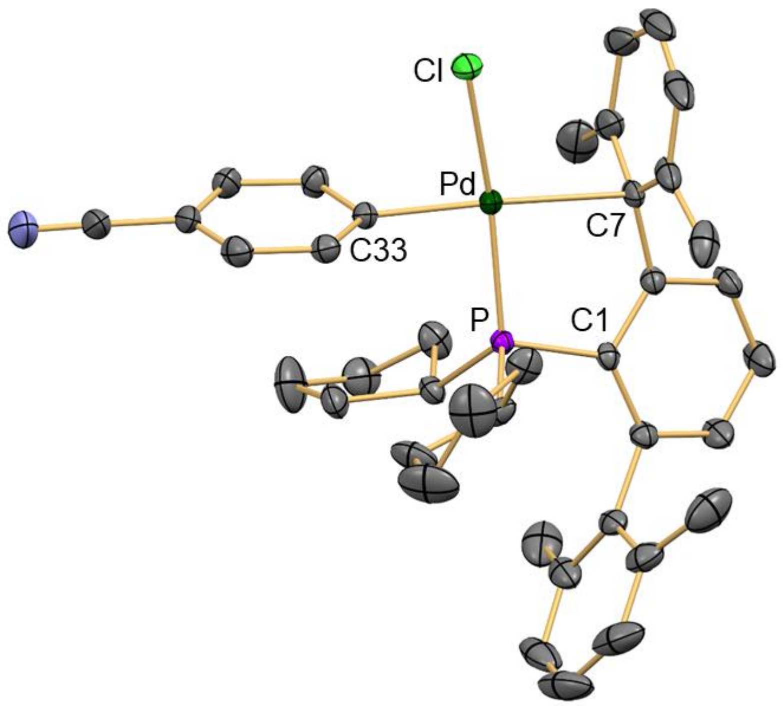

Figure S27. Molecular structure of complex $\mathbf{3^{CN}}$. Hydrogen atoms are omitted for clarity and thermal ellipsoids are set at 50% level probability. Selected distances [Å] and angles [°]: Pd-P

2.2617(7), Pd-Cl 2.3471(7), Pd-C7 2.457(2), Pd-C33 1.992(3), P-C1 1.851(2); Cl-Pd-C33 83.68(7), P-Pd-C7 83.06(7), P-Pd-Cl 169.32(3), C7-Pd-C33 162.17(10).

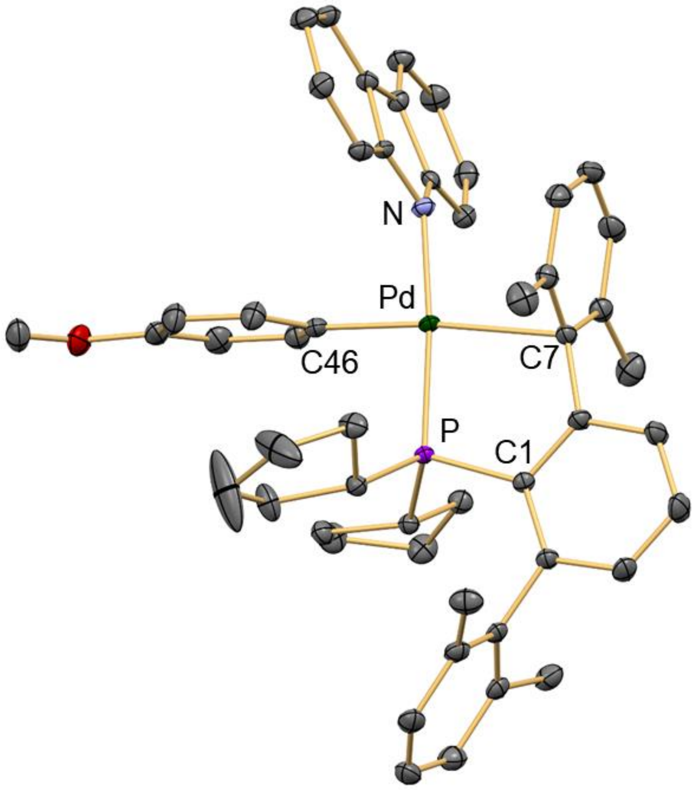

Figure S28. Molecular structure of complex **8^{OMe}Cz**. Hydrogen atoms are omitted for clarity and thermal ellipsoids are set at 50% level probability. Selected distances [Å] and angles [°]: Pd-P 2.2755(7), Pd-N 2.054(2), Pd-C7 2.456(3), Pd-C46 2.004(3), P-C1 1.853(3); N-Pd-C46 83.62(10), P-Pd-C7 82.92(6), C7-Pd-C33 162.17(10).

6. References

(1) Frisch, M. J.; Trucks, G. W.; Schlegel, H. B.; Scuseria, G. E.; Robb, M. A.; Cheeseman, J. R.; Scalmani, G.; Barone, V.; Petersson, G. A.; Nakatsuji, H.; Li, X.; Caricato, M.; Marenich, A. V.; Bloino, J.; Janesko, B. G.; Gomperts, R.; Mennucci, B.; Hratch, D. J. Gaussian 16, Revision B.01. Gaussian, Inc., Wallingford CT, 2016.

(2) Zhao, Y.; Truhlar, D. G. The M06 Suite of Density Functionals for Main Group Thermochemistry, Thermochemical Kinetics, Noncovalent Interactions, Excited States, and Transition Elements: Two New Functionals and Systematic Testing of Four M06-Class Functionals and 12 Other Function. *Theor. Chem. Acc.* **2008**, *120*, 215–241.

(3) Weigend, F.; Ahlrichs, R. Balanced Basis Sets of Split Valence, Triple Zeta Valence and Quadruple Zeta Valence Quality for H to Rn: Design and Assessment of Accuracy. *Phys. Chem. Chem. Phys.* **2005**, *7* , 3297.

(4) Weigend, F. Accurate Coulomb-Fitting Basis Sets for H to Rn. *Phys. Chem. Chem. Phys.* **2006**, *8*, 1057.

(5) Marenich, A. V.; Cramer, C. J.; Truhlar, D. G. Universal Solvation Model Based on Solute Electron Density and on a Continuum Model of the Solvent Defined by the Bulk Dielectric Constant and Atomic Surface Tensions. *J. Phys. Chem. B* **2009**, *113*, 6378–6396.

(6) Jensen, J. H. Predicting Accurate Absolute Binding Energies in Aqueous Solution: Thermodynamic Considerations for Electronic Structure Methods. *Phys. Chem. Chem. Phys.* **2015**, *17*, 12441–12451.

(7) Kim, S. T.; Kim, S.; Baik, M. H. How Bulky Ligands Control the Chemoselectivity of Pd-Catalyzed N-Arylation of Ammonia. *Chem. Sci.* **2020**, *11*, 1017–1025.

(8) Gómez-Orellana, P.; Lledós, A.; Ujaque, G. Computational Analysis on the Pd-Catalyzed C–N Coupling of Ammonia with Aryl Bromides Using a Chelate Phosphine Ligand. *J. Org. Chem.* **2021**, *86*, 4007–4017.

(9) Halaška, V.; Lochmann, L.; Lím, D. Association Degree of T-Butoxides of Alkali Metals in Aprotic Solvents. *Collect. Czechoslov. Chem. Commun.* **1968**, *33*, 3245–3253.

(10) Kissling, R. M.; Gagné, M. R. Structure and Reactivity of Mixed Alkali Metal Alkoxide/Aryloxide Catalysts. *J. Org. Chem.* **2001**, *66*, 9005–9010.

(11) Liu, W. B.; Schuman, D. P.; Yang, Y. F.; Toutov, A. A.; Liang, Y.; Klare, H. F. T.; Nesnas, N.; Oestreich, M.; Blackmond, D. G.; Virgil, S. C.; Banerjee, S.; Zare, R. N.; Grubbs, R. H.; Houk, K. N.; Stoltz, B. M. Potassium Tert-Butoxide-Catalyzed Dehydrogenative C-H Silylation of Heteroaromatics: A Combined Experimental and Computational Mechanistic Study. *J. Am. Chem. Soc.* **2017**, *139*, 6867–6879.

(12) Rama, R. J.; Maya, C.; Nicasio, M. C. Dialkylterphenyl Phosphine- Based Palladium Precatalysts for Efficient Aryl Amination of N - Nucleophiles. *Chem. – A Eur. J.* **2020**, *26*, 1064–1073.

(13) Martín, M. T.; Marín, M.; Rama, R. J.; Álvarez, E.; Maya, C.; Molina, F.; Nicasio, M. C. Zero-Valent ML 2 Complexes of Group 10 Metals Supported by Terphenyl Phosphanes. *Chem. Commun.* **2021**, *57*, 3083–3086.

(14) Monti, A.; Rama, R. J.; Gómez, B.; Maya, C.; Álvarez, E.; Carmona, E.; Nicasio, M. C. N-Substituted Aminobiphenyl Palladacycles Stabilized by Dialkylterphenyl

Phosphanes: Preparation and Applications in C N Cross-Coupling Reactions. *Inorganica Chim. Acta* **2021**, *518*, 120214.

(15) Rama, R. J.; Maya, C.; Nicasio, M. C. Palladium-Mediated Intramolecular Dearomatization of Ligated Dialkylterphenyl Phosphines. *Dalt. Trans.* **2019**, *48*, 14575–14579.

(16) Hoops, S.; Sahle, S.; Gauges, R.; Lee, C.; Pahle, J.; Simus, N.; Singhal, M.; Xu, L.; Mendes, P.; Kummer, U. COPASI--a COmplex PAthway SImulator. *Bioinformatics* **2006**, *22*, 3067–3074.

(17) Petzpold, L. Automatic Selection of Methods for Solving Stiff and Nonstiff Systems of Ordinary Differential Equations. *SIAM J. Sci. Stat. Comput.* **1983**, *4*, 136–148.

(18) Sheldrick, G. M. SADABS, Program for Empirical Absorption Correction of Area Detector Data. Göttingen: University of Göttingen, Germany 1996.

(19) APEX Suite of Crystallographic Software. APEX 2 Version 2008.4. Bruker AXS Inc. Madison, Wisconsin, USA 2008.

(20) Dolomanov, O. V.; Bourhis, L. J.; Gildea, R. J.; Howard, J. A. K.; Puschmann, H. OLEX2 : A Complete Structure Solution, Refinement and Analysis Program. *J. Appl. Crystallogr.* **2009**, *42*, 339–341.

(21) Sheldrick, G. M. Crystal Structure Refinement with SHELXL. *Acta Crystallogr. Sect. C Struct. Chem.* **2015**, *71*, 3–8.
